# High responsivity IR sensing based on reflectometric RF MEMS

**DOI:** 10.1038/s41467-026-74152-3

**Published:** 2026-06-16

**Authors:** Melisa E. Gülseren, Matthew Benson, Zhixing Lin, Tianyou Li, William P. Putnam, J. Sebastian Gomez-Diaz

**Affiliations:** Department of Electrical and Computer Engineering, University of California, Davis, Davis, CA USA

**Keywords:** Optical sensors, Microresonators, Mid-infrared photonics

## Abstract

Radiofrequency microelectromechanical systems (RF MEMS) integrated with metasurfaces are promising platforms for spectrally selective infrared (IR) sensing. Conventional devices detect IR radiation by tracking resonance frequency shifts. Here, we introduce a reflectometric approach that instead monitors changes in the RF MEMS input impedance and analytically links them to sensing metrics. By coupling a reconfigurable matching network to an RF MEMS resonator, the detector achieves IR responsivities governed by its phase-slope quality factor and tunable to exceptionally high values. Using contour-mode resonators at ambient conditions, we demonstrate responsivities exceeding 11,400 V/W in a 50-Ω readout (>200 A/W), spectral selectivity with a full-width at half-maximum (FWHM) of 0.54 μm at 5.94 μm, noise-equivalent power (NEP) of $\sim 450\ {{{\rm{pW}}}}/\sqrt{{{{\rm{Hz}}}}}$, a ~ 552-μs time constant, and resolve IR power levels down to ~ 740 pW. Reflectometric RF MEMS detectors provide a reconfigurable, spectrally selective, and scalable platform for high-performance on-chip IR spectroscopy and sensing.

## Abstract (web summary)

Researchers used RF reflectometry to achieve record infrared responsivity and low noise in RF-MEMS sensors. This high-performance platform enables scalable on-chip spectroscopy, paving the way for advanced and portable sensing applications.

## Introduction

IR sensing^1,2^ is critical in a broad range of applications, including spectroscopy, environmental monitoring, chemical analysis, and medical diagnostics, with ongoing efforts focused on improving responsivity, spectral selectivity, and noise performance at ambient conditions. Conventional photodetectors based on narrow-bandgap semiconductors, such as InSb, InGaAs, and HgCdTe^2–6^, offer fast response times, noise equivalent power (NEP) values down to ~${{{\rm{fW}}}}/\sqrt{{{{\rm{Hz}}}}}$ levels, and broadband responsivity reaching peak values up to 5 A/W in the mid-IR band. However, they typically require cryogenic cooling and rely on expensive and complex fabrication processes. Emerging two-dimensional (2D) materials promise room-temperature operation and tunable band gaps, with reported responsivities exceeding 10^3 ^A/W^7–11^ offset by highly variable NEP values. Despite outstanding metrics, large-scale integration of 2D material detectors remains hindered by device-to-device variability and susceptibility to local defects, particularly in photogating-based architectures^8,12^. Thermal detectors, such as bolometers^13^, achieve IR detection through thermally induced resistance changes and provide NEP values in the ${{{\rm{pW}}}}/\sqrt{{{{\rm{Hz}}}}}$ range. Although these sensors exhibit high voltage responsivities^2,14,15^ up to 10^5 ^V/W, their large output resistance (often >100 kΩ) results in poor impedance matching and current responsivities (A/W) comparable to or below those of semiconductor photodetectors^2^. Ultimately, translating IR sensing technologies into scalable, cost-effective, and on-chip IR spectroscopy and sensing systems^1,2,16^ requires transitioning from discrete, broadband devices to integrated, spectrally-selective detectors. A high responsivity is essential to overcome the noise floor of the interfacing electronics and enable operation near the detector’s intrinsic NEP.

In this context, thermal IR sensing based on RF MEMS^17–20^ loaded with ultrathin metasurfaces has attracted significant attention^21–31^. These devices embed IR spectral selectivity at the sensor level and avoid bulky alternatives like interferometry or optical filters/gratings that might also be difficult to align^32^. To date, the operation principle of RF MEMS-based IR sensors has relied on monitoring frequency shifts of specific mechanical modes upon IR illumination^33^, using either optical^26–31^ or electrical readouts^21–25^, yielding NEP values down to ~${{{\rm{pW}}}}/\sqrt{{{{\rm{Hz}}}}}$ levels and frequency stability limited by thermal fluctuation noise^21–23,34,35^. In these devices, IR responsivity and thermal NEP scale directly with and inversely with the square root of thermal resistance, respectively, whereas the device size determines speed and bandwidth. Despite promising performance, several challenges have hindered broader adoption of this technology. First, integrating readout mechanisms on-chip remains a challenge: optical schemes require bulky interferometric setups incompatible with chip-scale platforms, and electrical readouts must resolve small RF shifts. This latter challenge imposes a performance trade-off in this technology. Devices operating at low RF frequencies usually offer limited bandwidth and IR responsivity but achieve ultralow NEP values by using precise frequency tracking based on phase-locked loop (PLL) circuits^33^. High-frequency RF MEMS, operating at GHz or 100s of MHz, provide larger responsivity and faster response. Nevertheless, PLL tracking becomes constrained at these frequencies by an elevated phase noise that increases the NEP. Second, many studies have emphasised the development of key device innovations instead of focusing on systematic sensing characterisation procedures^5^. Figures of merit, such as responsivity and NEP, are often presented without considering their strong dependence on IR wavelength, power, or modulation frequency, leading to performance metrics that may not reflect realistic operating conditions. As a result, the potential performance of RF MEMS-based IR sensors for chip-integrated IR spectroscopy has yet to be fully explored in practice.

Here, we introduce a paradigm for high-responsivity IR sensing based on tracking the variations of the RF MEMS input impedance at a single RF frequency and mapping them into sensing performance metrics. To this purpose, we merge RF MEMS-based IR sensors with RF reflectometry^36–39^, a technique that enables highly sensitive detection of reactance variations by monitoring the phase shift of a reflected RF tone, and that can operate up to RF frequencies well into the GHz regime. In our approach, the IR detector is composed of an electrically tunable matching network coupled to the MEMS resonator. The device is excited at resonance with an RF tone, and the phase delay of the reflected signal is measured in a differential configuration as a function of the incident IR radiation. The output voltage is delivered in a 50-Ω circuit, ensuring compatibility with standard readout electronics. We show both theoretically and experimentally that responsivity scales linearly with the device’s phase-slope quality factor, thus enabling reconfigurable and high-performing IR sensing. For example, by tuning the matching network, the dynamic range of the detector can be adjusted; a well-matched device can provide high responsivity for low-power signals, while a detuned device can detect higher-power signals without saturation. We also show that flicker 1/f noise dominates the platform’s response and that it is inversely proportional to the device reflection coefficient. As proof of concept, we demonstrate reflectometric 202-MHz lateral CMR MEMS detectors operating in the mid-IR and characterise them using frequency, wavelength, and power-dependent approaches^5^. These devices are compatible with standard fabrication processes and achieve a responsivity over 11,400 V/W in a 50-Ω readout system (> 200 A/W) centred at 5.94 μm with a full-width half maximum (FWHM) of 0.54 μm, NEP down to $\sim 450\ {{{\rm{pW}}}}/\sqrt{{{{\rm{Hz}}}}}$, and a time constant of ~ 552 μs. We show that this scalable and electrically tunable platform enables room-temperature IR detection of power levels as low as ~ 740 pW, offering high-performing solutions for integrated IR spectroscopy and sensing applications.

## Results

### Operation principle

**Fig. 1:**
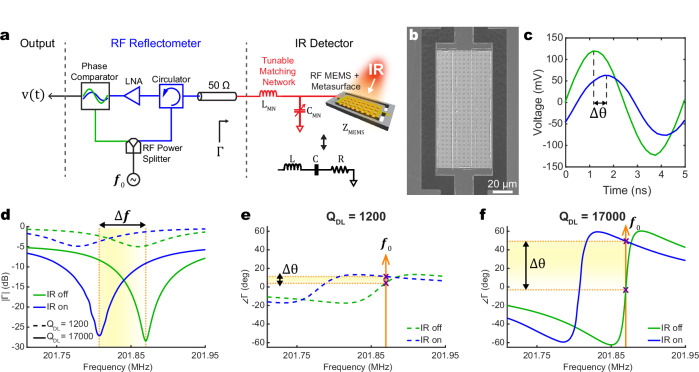
Operation principle of reflectometric RF MEMS-based IR detectors. **a** Schematic. The detector is composed of an AlN CMR RF MEMS resonator (decorated with an IR absorptive metasurface) that is coupled to a tunable LC matching network. The MEMS is characterized with an impedance *Z*_*MEMS*_ via a Modified Butterworth-Van Dyke (MBVD) network. Around resonance, the detector is modeled using an equivalent RLC resonator network that possesses a phase-slope quality factor Q_DL_. The reflectometric readout is composed of a generator that supplies an RF signal at *f*_*0*_ that is split into two paths: one feeds the IR detector and is reflected back through a circulator (blue line) and the other serves as a reference (green line). An RF phase comparator generates a voltage signal v(t) that is proportional to the phase difference between the two RF tones. The detector is illuminated with a coherent IR beam that is tunable in terms of wavelength and power (see Supplementary Information). **b** Scanning electron microscope image of the RF MEMS. The sensing area is 140 μm x 60 μm. **c** Time-domain measurement of the RF tones serving as a reference (green line) and reflected from the MEMS (blue line). The phase-slope quality factor of the device is ${{{{\rm{Q}}}}}_{{{{\rm{DL}}}}}=1200$, the frequency and power of the RF tone are set to 201.86 MHz and – 7 dBm, respectively, and the incoming IR beam at ${\lambda }_{0}=5.94\,{{{\rm{\mu }}}}{{{\rm{m}}}}$ has a total optical power ${{{{\rm{P}}}}}_{{{{\rm{IR}}}}}=750\,{{{\rm{\mu }}}}{{{\rm{W}}}}$ on the sensing area. **d**, **f** Reflection coefficient Γ in magnitude (**d**) and phase (**e**, **f**). Results are plotted when the device’s phase-slope quality factor is set to ${{{{\rm{Q}}}}}_{{{{\rm{DL}}}}}=1200$ (dashed lines) and ${{{{\rm{Q}}}}}_{{{{\rm{DL}}}}}=17,000$ (solid lines) considering no IR light (green lines) and a focused and coherent IR beam as described in panel **c** (blue lines). Circuit parameters are ${{{\rm{R}}}}=186.5\,\Omega$, ${{{\rm{L}}}}=297.3\,{{{\rm{\mu }}}}{{{\rm{H}}}}$, ${{{\rm{C}}}}=2.09\ {{{\rm{fF}}}}$ for ${{{{\rm{Q}}}}}_{{{{\rm{L}}}}}=$1200, and ${{{\rm{R}}}}={{\mathrm{56.89.8}}}\,\Omega$, ${{{\rm{L}}}}=97.635\,{{{\rm{\mu }}}}{{{\rm{H}}}}$, ${{{\rm{C}}}}=6.383\,{{{\rm{fF}}}}$ for ${{{{\rm{Q}}}}}_{{{{\rm{DL}}}}}={{\mathrm{17,000}}}$.

The operation principle of reflectometric RF MEMS-based IR detectors is illustrated in Fig. 1. The RF MEMS resonator considered here is shown in Fig. 1b and consists of a 1 μm-thick aluminum nitride (AlN) CMR, with resonance frequency ${{{{\rm{f}}}}}_{0}=201.86\,{{{\rm{MHz}}}}$ and an unloaded quality factor ${{{{\rm{Q}}}}}_{{{{\rm{MU}}}}}=2200$, integrated with a metasurface targeting IR absorption at ${\lambda }_{0}=5.94\,{{{\rm{\mu }}}}{{{\rm{m}}}}$ (Methods). The reflectometric detector is constructed by coupling a tunable matching network to the RF MEMS, as shown in Fig. 1a. Near its resonance, the detector response can be accurately described by an equivalent RLC circuit, characterised by a phase-slope quality factor Q_DL_ that is tunable via the matching network. Fig. 1d–f shows the frequency-dependent magnitude and phase of the detector reflection coefficient for two representative values of Q_DL_ (green curves). In our quality-factor notation, subscripts M, D, L, and U correspond to MEMS resonator, detector, loaded (phase-slope), and unloaded (energy-storage), respectively.

Let us consider that an incident IR beam at *λ*_0_ = 5.94 μm impinges onto the device. The MEMS resonance frequency undergoes a frequency shift $\Delta {{{\rm{f}}}}\left({{{\rm{\omega }}}},{{{\rm{\lambda }}}}\right)={{{{\rm{f}}}}}_{0}\cdot {{{\rm{TCF}}}}\cdot \Delta {{{\rm{T}}}}\left({{{\rm{\omega }}}},{{{\rm{\lambda }}}}\right)$, where TCF = −22.73 ppm/K is the temperature coefficient of frequency (TCF) measured in the device, and the temperature increase is given by $\Delta {{{\rm{T}}}}\left({{{\rm{\omega }}}},{{{\rm{\lambda }}}}\right)={{{\rm{\eta }}}}\left({{{\rm{\lambda }}}}\right)\cdot {{{\rm{\chi }}}}\left({{{\rm{\omega }}}}\right)\cdot {{{{\rm{P}}}}}_{{{{\rm{IR}}}}}\left({{{\rm{\lambda }}}}\right)\cdot {{{{\rm{R}}}}}_{{{{\rm{th}}}}}$. In this equation, ${{{\rm{\eta }}}}\left({{{\rm{\lambda }}}}\right)$ is the device’s wavelength-dependent absorptance in the IR band; $\chi \left(\omega \right)={\left(1+{{{{\rm{\omega }}}}}^{2}{{{{\rm{C}}}}}_{{{{\rm{th}}}}}^{2}{{{{\rm{R}}}}}_{{{{\rm{th}}}}}^{2}\right)}^{-\frac{1}{2}}$ captures the device response versus the modulation frequency *ω* of the incoming IR beam; R_th_ and C_th_ are the MEMS thermal resistance and capacitance, respectively, and ${{{{\rm{P}}}}}_{{{{\rm{IR}}}}}\left(\lambda \right)$ is the total IR power incident on the device–calculated as the power density of the beam integrated over the sensing area. If the IR detector is excited by an RF tone, it is evident from Fig. 1d–f that the magnitude and phase variations of the reflected signal will strongly depend on Q_DL_. We experimentally and numerically verified that the frequency shift linearly depends on the power of the incident IR beam but is independent of Q_DL_ (Supplementary Information). In terms of frequency shift, as previously explored in the literature^21,23,24^, the detector responsivity is obtained as ${{{{\rm{R}}}}}_{{{{\rm{f}}}}}\left({{{\rm{\omega }}}},{{{\rm{\lambda }}}}\right)\,=\Delta {{{\rm{f}}}}/{{{{\rm{P}}}}}_{{{{\rm{IR}}}}}={{{{\rm{f}}}}}_{0}\cdot {{{\rm{TCF}}}}\cdot {{{\rm{\eta }}}}\left({{{\rm{\lambda }}}}\right){\cdot {{{\rm{\chi }}}}\left({{{\rm{\omega }}}}\right)\cdot {{{\rm{R}}}}}_{{{{\rm{th}}}}}[{{{\rm{Hz}}}}/{{{\rm{W}}}}]$. Using an unmodulated IR beam $\left({{{\rm{\omega }}}}\to 0\right)$, we determine a responsivity of ${{{{\rm{R}}}}}_{{{{\rm{f}}}}}=124{{\ {\rm{Hz}}}}/{{{\rm{uW}}}}$ and an effective thermal resistance ${{{{\rm{R}}}}}_{{{{\rm{th}}}}}=31.4\,\cdot {10}^{3}{{\ {\rm{K}}}}/{{{\rm{W}}}}.$ In this type of MEMS, thermal resistance is dominated by the thermal conductance of the anchors^19,20^. These values are in good agreement with the ones obtained using theoretical calculations and multi-physics numerical simulations (Supplementary Information).

### IR responsivity based on RF reflectometry

Instead of relying on frequency shift-based responsivity, we introduce and define here the responsivity of reflectometric IR detectors and demonstrate both theoretically and experimentally that it can reach exceptionally high values at room temperature. We begin our analysis by considering an RF signal exciting the IR detector exactly at its resonance frequency f_0_. Upon absorbing IR light, the signal reflected from the device undergoes a relative phase change (Supplementary Information)

1 $$\Delta \measuredangle \Gamma \left({{{\rm{\omega }}}},{{{\rm{\lambda }}}}\right)=2\cdot {{{{\rm{Q}}}}}_{{{{\rm{DL}}}}}\cdot {{{\rm{TCF}}}}\cdot {{{\rm{\eta }}}}\left({{{\rm{\lambda }}}}\right)\cdot {{{{\rm{P}}}}}_{{{{\rm{IR}}}}}\left({{{\rm{\lambda }}}}\right)\cdot {{{\rm{\chi }}}}\left({{{\rm{\omega }}}}\right)\cdot {{{{\rm{R}}}}}_{{{{\rm{th}}}}}\,\left[{{{\rm{rad}}}}\right].$$

The detector responsivity is then defined as ${{{{\rm{R}}}}}_{\Delta \measuredangle }\left({{{\rm{\omega }}}},{{{\rm{\lambda }}}}\right)=\Delta \measuredangle \Gamma \left({{{\rm{\omega }}}},{{{\rm{\lambda }}}}\right)\,/{{{{\rm{P}}}}}_{{{{\rm{IR}}}}}\left({{{\rm{\lambda }}}}\right)\,\left[{{{\rm{rad}}}}/{{{\rm{W}}}}\right]$. Relative phase changes can be obtained by resolving the phase difference between a reference RF tone and the reflected signal (Fig. 1c). The detector responsivity can then be calculated as

2 $${{{{\rm{R}}}}}_{{{{\rm{v}}}}}\left({{{\rm{\omega }}}},{{{\rm{\lambda }}}}\right)=2\cdot {{{\rm{H}}}}\cdot {{{{\rm{Q}}}}}_{{{{\rm{DL}}}}}\cdot {{{\rm{TCF}}}}\cdot {{{\rm{\eta }}}}\left({{{\rm{\lambda }}}}\right)\cdot {{{\rm{\chi }}}}\left({{{\rm{\omega }}}}\right)\cdot {{{{\rm{R}}}}}_{{{{\rm{th}}}}}\ [{{{\rm{V}}}}/{{{\rm{W}}}}],$$

where H [V/rad] is the transfer function of the RF phase comparator. Equation (2) reveals that the responsivity is given in V/W units and that its magnitude can be manipulated by (i) adjusting the detector Q_DL_ through properly designed electrical matching circuits; (ii) increasing the device thermal resistance; and (iii) employing RF phase comparators with high gain. Remarkably, the platform provides an output voltage via a phase comparator with 50-Ω output impedance, ensuring compatibility with standard RF readout systems.

**Fig. 2:**
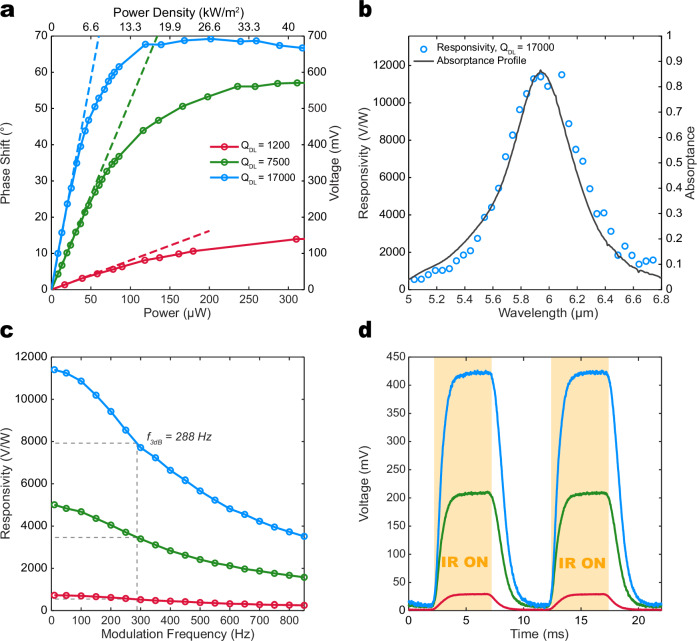
Responsivity of reflectometric RF MEMS-based IR detectors. **a** Relative phase change between a reference RF tone and the RF signal reflected on the IR detector–see schematic in Fig. 1a–versus the power of an incoming IR beam at ${\lambda }_{0}=5.94\,{{{\rm{\mu }}}}{{{\rm{m}}}}$. Right *y*-axis shows the measured voltage at the output of the RF comparator. Dashed lines, capturing the detector response in the linear regime, are calculated using Eq. (1). **b** IR spectral selectivity of the detector with phase-slope quality factor ${{{{\rm{Q}}}}}_{{{{\rm{DL}}}}}={{\mathrm{17,000}}}$. Solid black lines show the measured absorption profile of the device. Blue markers show wavelength-dependent responsivities in V/W obtained by relating the output voltage from the RF comparator to the power of the incoming IR beam. **c** Detector peak-to-peak responsivity in V/W versus the modulation frequency of the incoming IR beam. Results reveal a cut-off frequency of ${{{{\rm{f}}}}}_{{{{\rm{cutoff}}}}}=288\,{{{\rm{Hz}}}}$. **d** Detector output voltage versus time when it is illuminated by an IR beam at ${\lambda }_{0}=5.94\,{{{\rm{\mu }}}}{{{\rm{m}}}}$, chopped at 100 Hz, and with a total power over the sensor area of ${{{{\rm{P}}}}}_{{{{\rm{in}}}}}=40\,{{{\rm{\mu }}}}{{{\rm{W}}}}$. Results are plotted for various values of the detector’s phase-slope quality factor Q_DL_.

Figure 2 presents a comprehensive responsivity study of reflectometric MEMS-based IR detectors. Fig. 2a shows the phase shift of the RF signal reflected on the device versus the power of an unmodulated IR beam at *λ*_0_ = 5.94 μm. As expected, the phase difference follows a linear relation with respect to moderately low IR powers. The slope of this curve represents the detector responsivity in phase, ${{{{\rm{R}}}}}_{\Delta \measuredangle }\left({{{\rm{\omega }}}},{{{\rm{\lambda }}}}\right)$, and depends on Q_DL_. Linear responsivity values of ${{{{\rm{R}}}}}_{\Delta \measuredangle }=$ $72.6\cdot {10}^{3}$, $501\cdot {10}^{3}$ and $1140\cdot {10}^{3}$ [deg/W] are obtained for increasingly larger Q_DL_ values of ~1200, ~7500, and ~17,000, respectively. To convert RF phase differences into a DC voltage, our setup employs a commercial ADI AD8302 chip that provides a transfer function of H = 10 mV/deg (Supplementary Information). The right y-axis of Fig. 2a shows the measured output voltage that corresponds to responsivities of ${{{{\rm{R}}}}}_{{{{\rm{v}}}}}=726$, 5010, and 11400 [V/W] in the linear regime. These metrics are in good agreement with theoretical predictions from Eq. (2) and with circuit and multi-physics numerical simulations (Supplementary Information). As the IR power increases further, the device resonance shifts away from the frequency of the RF signal and brings the detector response into a regime that does not linearly depend on the IR power. This nonlinear regime originates from the finite bandwidth of the RF resonance and not from the intrinsic nonlinear response of the materials composing the RF MEMS/metasurface structure. The saturation power P_sat_ follows ${{{{\rm{P}}}}}_{{{{\rm{sat}}}}}\propto \frac{1}{{{{{\rm{Q}}}}}_{{{{\rm{DL}}}}}}$ and leads to an adjustable dynamic range: larger Q_DL_ increases the detector responsivity but limits the maximum detectable IR power. In our device, IR power saturation decreases from approximately 100 µW to 65 µW and 47 µW, corresponding to power densities of 11.9, 7.74, and 5.36 kW/m^2^, as Q_DL_ increases.

Figure 2b shows the wavelength-dependent responsivity and absorption profile of the device. These measurements demonstrate that the IR spectral selectivity of the metasurface directly translates into the detector responsivity, and that substrate heating from incident radiation does not have a significant effect on the detection process. Peak responsivity is achieved at 5.94 µm with a FWHM of 545 nm, providing spectrally selective detection without the need for external optical filters. Such intrinsic narrowband operation is well suited for sensing modalities that operate at discrete wavelengths, including molecular gas sensing, active imaging, and laser-based detection systems, where enhanced selectivity and reduced background loading are critical. Fig. 2c plots the IR detector responsivity for various Q_DL_ values versus the frequency ω of an optical chopper that modulates an incoming IR beam. Results reveal a cut-off frequency and time constant of 288 Hz and 552 μs, respectively, associated with a thermal capacitance ${{{{\rm{C}}}}}_{{{{\rm{th}}}}}=17.58\ {{{\rm{nJ}}}}/{{{\rm{K}}}}$. These values agree well with the typical speed of CMR sensors reported in the literature^19,20^. The detector output voltage upon chopped IR light is plotted in Fig. 2d versus time.

### RF signal reflected from the detector: noise mechanisms and NEP

**Fig. 3:**
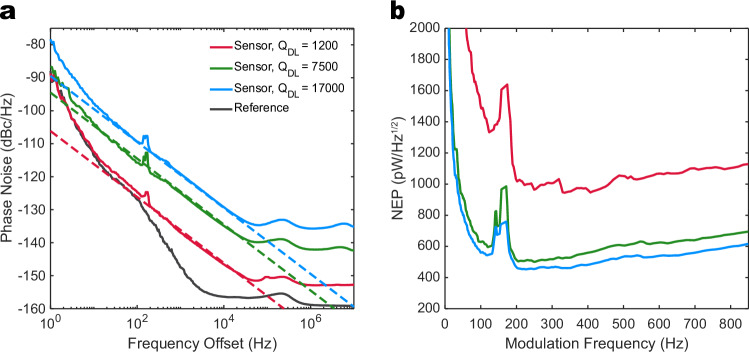
Noise response and NEP of reflectometric RF MEMS-based IR detectors determined by analyzing RF signals. **a** Phase noise of the reference RF tone (black solid line) and the RF signal reflected from the IR detector (colored solid lines). Dashed lines show the flicker 1/f noise component of the reflected RF signals predicted by Eq. (4). Results confirm that the flicker noise dominates the detector noise response and that the noise increases with the detector phase-slope quality factor, Q_DL_. The RF operation frequency is set exactly at the resonance frequency of each device. **b** Detector’s noise equivalent power versus the modulation frequency of an incoming IR beam at ${\lambda }_{0}=5.94\,{{{\rm{\mu }}}}{{{\rm{m}}}}$, calculated by relating measured phase noise responsivity values following Eq. (5). Results are plotted for various values of the detector’s phase-slope quality factor, Q_DL_.

To explore the noise response of reflectometric MEMS-based IR detectors, we focus first on the phase noise^40^ of the RF signal that is reflected from the IR device. It can be expressed as

3 $${{{{\rm{\phi }}}}}_{{{{\rm{n}}}}}^{2}\left({{{\rm{\omega }}}}\right)={{{{\rm{\phi }}}}}_{{{{\rm{RF}}}}}^{2}\left({{{\rm{\omega }}}}\right)+{{{{\rm{\phi }}}}}_{{{{\rm{MEMS}}}}}^{2}\left({{{\rm{\omega }}}}\right)\ \left[{{{{\rm{rad}}}}}^{2}/{{{\rm{Hz}}}}\right],$$

where ${{{{\rm{\phi }}}}}_{{{{\rm{RF}}}}}\left({{{\rm{\omega }}}}\right)$ is the phase noise of the RF signal that excites the device. This noise depends on the type of generator employed to synthesize the tone and will be mostly removed by using a common-mode noise suppression scheme, as described below. In Eq. (3), ${{{{\rm{\phi }}}}}_{{{{\rm{MEMS}}}}}\left({{{\rm{\omega }}}}\right)$ accounts for the phase noise added to the reflected RF signal by the reflectometric device. Several mechanisms compose ${{{{\rm{\phi }}}}}_{{{{\rm{MEMS}}}}}\left({{{\rm{\omega }}}}\right)$, including flicker noise^41,42^, thermal noise^43^, and the thermomechanical noise generated by the motion of the MEMS^43^, among others^44^. In this platform, flicker noise–commonly known as 1/f noise–becomes dominant with respect to other noise components for devices with high Q_DL_, *i.e.*, ${{{{\rm{\phi }}}}}_{{{{\rm{MEMS}}}}}\left({{{\rm{\omega }}}}\right)\approx {{{{\rm{\phi }}}}}_{{{{\rm{flicker}}}}}\left({{{\rm{\omega }}}}\right)$, and can be modeled as (Supplementary Information)

4 $${{{{\rm{\phi }}}}}_{{{{\rm{flicker}}}}}^{2}\left({{{\rm{\omega }}}}\right)=\frac{2{{{\rm{\pi }}}}}{{{{{\rm{V}}}}}_{0}^{2}\cdot {\left|\Gamma \right|}^{2}}\frac{{{{{\rm{b}}}}}_{0}}{{{{\rm{\omega }}}}}\ [{{{{\rm{rad}}}}}^{2}/{{{\rm{Hz}}}}],$$

where Γ is the device’s reflection coefficient, V_0_ is the amplitude voltage of the incoming RF signal, and b_0_ is a device-specific constant that depends on the RF MEMS geometry, design, and thermal properties of the composing materials^42^. Such behavior appears due to the interplay between the power of the reflected RF signal, which decreases as Q_DL_ increases, and the power of the flicker noise originating within the MEMS resonator, ${{{{\rm{\phi }}}}}_{{{{\rm{flicker,MEMS}}}}}^{2}=\frac{{{{{\rm{b}}}}}_{0}}{{{{\rm{\omega }}}}}$, which is mostly independent of the RF signal^41,42^. Fig. 3a plots measured phase noise associated with a low-noise reference RF tone and with the RF tone reflected from the device for various Q_DL_. Results reveal that increasing the device Q_DL_ increases the phase noise of the reflected signal, as expected from Eq. (4). Such a response implies a performance trade-off: increasing Q_DL_ leads to larger IR responsivity values but also increases the overall detector’s noise response.

Once the detector’s noise and responsivity have been characterized, it is possible to determine the minimum IR power that can be detected using a modulated IR beam together with a phase-sensitive lock-in detection approach^45^. In this frequency-domain approach, the detection is locked onto a narrow bandwidth $\left({{{\rm{B}}}}=1\ {{{\rm{Hz}}}}\right)$ around the IR modulation frequency and avoids noise contributions from unwanted frequency components. The detector NEP can be then defined as

5 $${{{\rm{NEP}}}}\left({{{\rm{\omega }}}},\,{{{\rm{\lambda }}}}\right)\,=\frac{{{{{\rm{\phi }}}}}_{{{{\rm{n}}}}}\left({{{\rm{\omega }}}}\right)}{{{{{\rm{R}}}}}_{\Delta \measuredangle }\left({{{\rm{\omega }}}},{{{\rm{\lambda }}}}\right)\cdot {{{{\rm{c}}}}}_{1}}\,\left[{{{\rm{W}}}}\cdot {{{{\rm{Hz}}}}}^{-1/2}\right],$$

where ${{{{\rm{c}}}}}_{1}=\frac{\sqrt{2}}{\pi }.$ The term ${{{{\rm{R}}}}}_{\Delta \measuredangle }\left({{{\rm{\omega }}}},{{{\rm{\lambda }}}}\right)\cdot {{{{\rm{c}}}}}_{1}$ represents the responsivity measured by the lock-in amplifier when detecting an IR beam chopped at frequency ω, and corresponds to the root means square amplitude of the first Fourier harmonic of a square wave modulation^46^ (Methods). Equation (5) reveals that the frequency-dependent response of NEP is critical in thermal sensors, as both their responsivity and noise rapidly change with the modulation frequency of the IR beam. Fig. 3b plots the NEP predicted by Eq. (5) using measured noise and responsivity data for detectors with different Q_DL_. At low modulation frequencies, the NEP is dominated by 1/f noise contributions from the RF MEMS despite the larger responsivity values offered by the detector in that range (Fig. 2c). As frequency increases, noise and responsivity frequency responses compensate each other, leading to a minimum NEP of $\sim 450\ {{{\rm{pW}}}}/\sqrt{{{{\rm{Hz}}}}}$ for modulation frequencies in the 200 to 400 Hz range. At higher modulation frequencies, the NEP slightly increases because the responsivity decreases while noise contributions remain relatively constant. Our study sheds light on the fundamental mechanisms that govern reflectometric IR detectors by analyzing the phase of RF signals. In the following, we describe how such RF behavior translates into baseband voltage signals.

### Noise, NEP, and detection limit in reflectometric RF MEMS-based IR detectors

The platform output is given by the voltage signal generated by an RF phase comparator, as illustrated in Fig. 1a. The voltage noise spectral density ${{{{\rm{v}}}}}_{{{{\rm{n}}}},{{{\rm{sys}}}}}\left({{{\rm{\omega }}}}\right)$ of this signal can be described by (Supplementary Information)

6 $${{{{\rm{v}}}}}_{{{{\rm{n}}}},{{{\rm{sys}}}}}^{2}\left({{{\rm{\omega }}}}\right)={{{{\rm{v}}}}}_{{{{\rm{n}}}},{{{\rm{PC}}}}}^{2}\left({{{\rm{\omega }}}}\right)+{{{{\rm{H}}}}}^{2}{{{{\rm{\phi }}}}}_{{{{\rm{flicker}}}}}^{2}\left({{{\rm{\omega }}}}\right)\left[{{{{\rm{V}}}}}^{2}\ {{{\rm{Hz}}}}\right],$$

where ${{{{\rm{v}}}}}_{{{{\rm{n}}}},{{{\rm{PC}}}}}\left({{{\rm{\omega }}}}\right)$ and H are the output voltage noise spectral density and transfer function of the phase comparator, respectively, and ${{{{\rm{\phi }}}}}_{{{{\rm{flicker}}}}}\left({{{\rm{\omega }}}}\right)$ is the flicker noise added by the RF MEMS as described in Eq. (4). In the proposed differential scheme, the phase noise of the RF source is a common-mode contribution that is effectively eliminated by the phase comparator. This feature reduces the platform noise response and permits the use of standard RF sources without deteriorating the sensing performance.

**Fig. 4:**
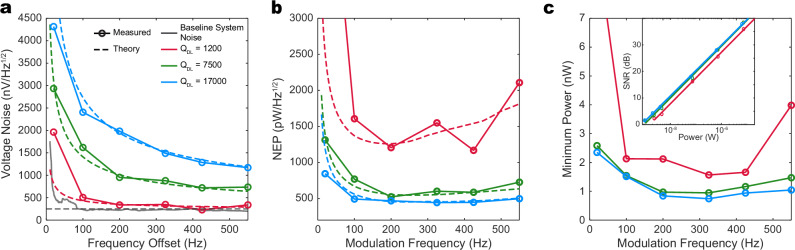
Noise response, NEP, and detection limit of reflectometric RF MEMS-based IR detectors. **a** Voltage noise spectral density versus offset frequency. Measured results (solid lines) are obtained at ambient conditions and room temperature in the absence of external IR radiation. Theoretical predictions (dashed lines) are obtained using Eq. (6). Black lines show the baseline noise associated with the system without including the RF MEMS device. **b** NEP versus the modulation frequency of an incoming IR beam. Solid (dashed) lines correspond to predicted (theoretical) NEP obtained by relating measured (theoretical) voltage noise spectral density and responsivity values following Eq. (7). **c** Minimum IR power detected using a phase-sensitive lock-in approach versus the modulation frequency of an incoming IR beam. The IR power incident on the device is then decreased until an SNR = 1 is found, as described in ref. ^5^ Inset shows the system SNR for IR beams modulated at 325 Hz. In all cases, results are reported for detectors with various Q_DL_ and an incoming IR beam at ${\lambda }_{0}=5.94\,{{{\rm{\mu }}}}{{{\rm{m}}}}$.

We begin by defining the baseline voltage noise spectral density in the system without considering the presence of the RF MEMS. This baseline noise is determined experimentally in Fig. 4a (black solid line) by using a modified setup in which a passive attenuator substitutes the RF MEMS device while providing similar return loss levels (Supplementary Information). As predicted by Eq. (6), baseline noise is governed by the noise response of the phase comparator, *i.e.*, ${{{{\rm{v}}}}}_{{{{\rm{n}}}},{{{\rm{sys}}}}}\left({{{\rm{\omega }}}}\right)\approx {{{{\rm{v}}}}}_{{{{\rm{n}}}},{{{\rm{PC}}}}}\left({{{\rm{\omega }}}}\right)$, with measured values hovering around $250\ {{{\rm{nV}}}}/ \sqrt{{{{\rm{Hz}}}}}$ that are in good agreement with the chip specifications^47^.

Figure 4a (colored solid lines) plots the voltage noise spectral density measured on devices for various Q_DL_ values. Again, results indicate that detectors with larger Q_DL_ possess higher noise. Specifically, the flicker noise of the reflected RF signal translates into a voltage noise spectral density larger than the one originating within the phase comparator. This 1/f noise component dominates the overall system response, *i.e.*, ${{{{\rm{v}}}}}_{{{{\rm{n}}}},{{{\rm{sys}}}}}\left({{{\rm{\omega }}}}\right)\approx {{{\rm{H}}}}\cdot \,{{{{\rm{\phi }}}}}_{{{{\rm{flicker}}}}}\left({{{\rm{\omega }}}}\right)$. Theoretical results obtained using Eq. (6) and plotted in Fig. 4a (dashed lines) agree well with measurements, further confirming our predictions. We have verified that the reported noise levels are stable versus the power and noise of the RF signal.

The detector NEP calculated as

7 $${{{\rm{NEP}}}}\left({{{\rm{\omega }}}},\,{{{\rm{\lambda }}}}\right)\,=\frac{{{{{\rm{v}}}}}_{{{{\rm{n}}}},{{{\rm{sys}}}}}\left({{{\rm{\omega }}}}\right)}{{{{{\rm{R}}}}}_{{{{\rm{v}}}}}\left({{{\rm{\omega }}}},{{{\rm{\lambda }}}}\right)\cdot {{{{\rm{c}}}}}_{1}}\,\left[{{{\rm{W}}}}\cdot {{{{\rm{Hz}}}}}^{-1/2}\right],$$

is plotted in Fig. 4b (solid lines) versus the modulation frequency of the incoming IR beam. Results, obtained by relating measured voltage noise spectral density and responsivity, are superimposed with theoretical predictions (dashed lines). Very good agreement between theory and measurements is found. Predicted NEP follows a similar pattern as in Fig. 3b, with minimum values hovering around $450\ {{{\rm{pW}}}}/\sqrt{{{{\rm{Hz}}}}}$ for IR beam modulation frequencies around 300 Hz.

To further explore the performance limit of this technology, we experimentally determine the minimum IR power that can be detected using a phase-sensitive lock-in approach (Fig. 4c, Methods). To this end, we measure the platform signal to noise ratio (SNR) versus the power of an incoming IR beam at ${{{{\rm{\lambda }}}}}_{0}=5.94\,{{{\rm{\mu }}}}{{{\rm{m}}}}$ modulated with an optical chopper (inset of Fig. 4c). The IR power incident on the device is then decreased until an SNR=1 is found, following the approach described in ref. ^5^. Experimental data reveals that IR power levels down to ~ 742 pW ( ~ 88.3 mW/m^2^) are detected at room temperature and ambient conditions. The minimum power detected in the platform follows similar trends versus Q_DL_ and modulation frequency of the IR beam as the NEP metrics discussed above. Minimum power levels are slightly higher than expected from NEP predictions, which we attribute to fluctuations of the IR beam intensity both in amplitude and space, potential environmental variations, and full-system mismatches. Our experiments validate the platform’s capabilities under realistic operational conditions by directly measuring the weakest detectable IR signal. Even though NEP predictions are widely adopted to characterize IR detector performance, as shown in Fig. 3b and Fig. 4b, they often rely on partial experimental characterization studies that may not fully capture complex mechanisms within the complete detection system–especially as the power of the IR beam is substantially attenuated and approaches environmental levels^5^.

## Discussion

Reflectometric RF MEMS-based IR detectors provide a robust platform for room-temperature IR sensing, combining high performance with mature and scalable technology. Peak current responsivity $\left({{{{\rm{R}}}}}_{{{{\rm{A}}}}}\approx 200\,{{{\rm{A}}}}/{{{\rm{W}}}}\right)$ is over 20 times larger than that from HgCdTe photodetectors in the mid-IR band^1,2,48^ and outperforms other thermal and semiconductor platforms by several orders of magnitude. Even though hybrid devices based on graphene-semiconductor interfaces^10^ or WS_2_/HfS_2_ heterojunctions^9^ have demonstrated responsivity up to 300 A/W and 1000 A/W, respectively, reflectometric IR detectors rely on established MEMS processes that enable seamless on-chip integration and scalability. In terms of noise and speed, reflectometric IR detectors achieve NEP levels comparable to leading bolometers $\left({ \sim \,10}^{-10}\ {{{\rm{W}}}}/\sqrt{{{{\rm{Hz}}}}}\right)$ but with faster response times (500 μs vs. ms range) and drastically improved impedance matching that ensures compatibility with standard RF readout systems. A trade-off between noise and bandwidth arises: larger (smaller) MEMS structures will lead to reduced (enhanced) flicker noise contributions^49^ and reach lower (higher) NEP values at the cost of slower (faster) responses.

A unique aspect of reflectometric IR detectors is their electrically reconfigurable performance, which is obtained by manipulating the capacitance of the matching network and thus adjusting the device Q_DL_. Lowering Q_DL_ increases the saturation power at the cost of reduced performance, allowing the device to operate under high-intensity IR illumination. In contrast to semiconductor photodetectors, which are often susceptible to irreversible photodamage^2^, the damage threshold in RF MEMS sensors is ultimately set by the photothermal limits of their constituent materials^50^. Additionally, and according to Eq. (2), there is plenty of room to further enhance the detector’s IR responsivity. For instance, devices with Q_DL_ > 10^5^ (>10^6^) can be obtained at MHz frequencies using fine-tuned lumped components at room temperature (~ 77 K), yielding voltage IR responsivities beyond 65 kV/W (650 kV/W) and current responsivities over 1300 A/W (13,000 A/W). Furthermore, MEMS with thermal resistance R_th_ > 10^7^ K/W can be developed using advanced anchor engineering approaches^20,51,52^, providing extra knobs to boost the IR responsivity. In terms of speed, the device’s thermal capacitance can be reduced by thinning the AlN layer in the CMR resonator down to ~ 250 nm, improving the time constant by a factor of four. To suppress noise, arrays of RF MEMS can be developed within the same released area to create differential architectures in which one device serves as a reference and others as sensors. In this configuration, 1/f noise may become a common-mode contribution that can be effectively canceled^41^, leaving the noise from the phase detector as the dominant noise source. Considering the metrics reported in Fig. 2 and Fig. 4, such an approach yields an ${{{\rm{NEP}}}}\approx 50\ {{{\rm{pW}}}}/\sqrt{{{{\rm{Hz}}}}}$ for IR beams modulated at 100 Hz. Replacing off-the-self components with dedicated RF phase-comparators and state-of-the-art noise cancellation techniques^53,54^ can further enhance performance. Custom CMOS circuits can achieve voltage noise spectral density below the $10\ {{{\rm{nV}}}}/\sqrt{{{{\rm{Hz}}}}}$ range while maintaining, or even surpassing, the phase gain of commercial RF comparators. Leveraging these approaches, reflectometric IR detectors have the potential to achieve giant IR responsivity and operate in the thermal fluctuation limit at ambient conditions.

More broadly, the proposed reflectometric approach extends the capabilities of IR thermal sensing by drastically enhancing the responsivity of RF MEMS-based technologies, like those based on CMRs^21,22,24^, film bulk acoustic resonators^23^ (FBAR), SiN drumhead resonators^25,26^, or doped aluminum scandium nitride (AlScN) nanoplates^30^, maintaining low noise and greatly simplifying their electrical readout. For example, these devices can be functionalized with tunable IR metasurfaces^55^ to enable spectrally selective IR scanning. Arrays of reflectometric MEMS-based detectors integrated within a thermally optimized package, each targeting a distinct IR wavelength, provide a compact and fully on-chip route toward reconstructive IR spectrometers and imaging systems, in which responsivity and saturation power can be dynamically reconfigured across IR bands to support adaptive and application-specific operation.

## Methods

### Device design

The RF MEMS-based IR sensor consists of a 3-electrode AlN CMR^19,20^ designed to resonate at 201.86 MHz that is decorated with a Pt/SiO_2_/Al metal-insulator-metal (MIM) metasurface^55^ with patch shaped nanostructures designed for infrared absorption at *λ*_0 _= 5.94 µm. Following the design guidelines of AlN CMR devices, the device resonance frequency is set to ${{{{\rm{f}}}}}_{0}=\frac{1}{2{{{{\rm{W}}}}}_{{{{\rm{elec}}}}}}\sqrt{\frac{{{{{\rm{E}}}}}_{{{{\rm{eq}}}}}}{{{{{\rm{\rho }}}}}_{{{{\rm{eq}}}}}}}$, where ρ_eq_ is the equivalent mass density, E_eq_ is the Young’s modulus of the material stack composing the resonator, and W_elec_ is the width of the electrodes forming the interdigital transducer (IDT). The other two main geometrical dimensions, length L_elec_ and thickness d_elec_, control the electrical capacitance, C_0_, and motional resistance, R_m_, of the resonator. The electrical equivalent of the resonator, modeled with a modified Butterworth–Van Dyke (MBVD) equivalent circuit model^20^, can be described in terms of the geometrical dimensions, C_0_, f_0_, and the figures of merit of the resonator, *i.e.*, the electromechanical coupling, ${{{{\rm{k}}}}}_{{{{\rm{t}}}}}^{2}$, and unloaded mechanical quality factor, Q_MU_. The design procedure consists of selecting the width W_elec_ to set the operation frequency and selecting the remaining parameters to maximize ${{{{\rm{k}}}}}_{{{{\rm{t}}}}}^{2}$ and Q_MU_. The device used in this work consists of a 3-electrode IDT with electrode width W_elec_ of 20 µm, electrode length L_elec_ of 140 µm, and thickness d_elec_ of 1 µm, resulting in ${{{{\rm{R}}}}}_{{{{\rm{s}}}}}=10\,\Omega$, ${{{{\rm{R}}}}}_{0}=697.68\,\Omega$, C_0_ = 558.62 fF, ${{{{\rm{R}}}}}_{{{{\rm{m}}}}}=173.0\,\Omega$, C_m _= 2.00 fF, and L_m_ = 310.85 µH, with ${{{{\rm{Q}}}}}_{{{{\rm{MU}}}}}\approx 2200$ and ${{{{\rm{k}}}}}_{{{{\rm{t}}}}}^{2}=0.44\%.\,$ Additional information regarding the resonator response and fitting is provided in the Supplementary Information, Section 1.

In AlN CMRs, the anchor dimensions are a critical design parameter to minimize anchor loss and obtain high-quality factor values^56^. Anchor widths are commonly tuned to be a fraction of the acoustic wavelength to effectively suppress acoustic leakage. This leads to anchor dimension designs short and narrow relative to the resonator body, with widths in the sub-micron to a few microns range depending on the operating frequency. The device employed in this work is tethered to the substrate with two anchors of width 20 µm and length 32 µm.

The metasurface decorating the RF MEMS is composed of an MIM structure, where the top electrode metal layer of the MEMS resonator also acts as the ground metal layer of the metasurface (Supplementary Information, Section 1.3). The ground layer is 100 nm of Pt, the dielectric or spacer layer is 200 nm SiO_2_, and the top layer is 100 nm of nanostructured Al. The metasurface presented in the main text is composed of square patches with a size of 1.87 µm and a periodicity of 4.5 µm.

### Device fabrication

The devices were fabricated on 6” high-resistivity ( > 10 kΩ) silicon wafers. First, the bottom electrodes were patterned using deep ultraviolet (DUV) lithography and liftoff of sputter deposited Cr/Pt (10/100 nm). The piezoelectric material composing the body of the MEMS resonator was formed through AC sputtering of 1-µm AlN. XRD analysis of the deposited AlN film confirmed good crystal quality with 1.45° FWHM for the (0002) peak. TCP etch with a Cl_2_ environment was used to form vias to the bottom electrodes, which was followed by the patterning of the Cr/Pt (10/100 nm) top electrodes using DUV lithography and liftoff. The top electrode of the MEMS resonator simultaneously acts as the ground plane of the MIM metasurface, for which the MIM stack was formed by subsequent deposition of 200-nm PECVD SiO_2_ and sputter deposition of 100-nm Al. The Al nanostructures composing the metasurface were patterned using DUV lithography and DPS etch. Vias to the top electrode were formed by patterning and ICP-RIE etch of the SiO_2_ layer. The resonator shape was defined by patterning and sequentially etching the SiO_2_ and AlN layers. Lastly, the devices were released from the Si substrate using a XeF_2_ etch. A schematic of the fabrication process is shown in Supplementary Information, Section 1.1.

### Matching network

The matching network is an L-section network consisting of a series inductor and a shunt capacitor. The network is matched to the MEMS impedance at its series resonance. A variable trimmer capacitor is used (2 to 10 pF) to achieve a more precise matching in the experiment, which is also convenient for quickly tuning the phase-slope quality factor of the IR detector, Q_DL_. The operation principle of the variable capacitor is mechanical and therefore does not add extra noise to the system. Two series inductors are added (43 nH and 9.1 nH), to provide precise inductance control. The network is implemented using SMD components soldered to a PCB (Supplementary Information, Section 1.4). The PCB contains transmission lines with pads for the two series inductors and a shunt variable trimmer capacitor. Electrical contact is made to the PCB via coaxial cables connected to SMA connectors that are soldered to the PCB. Finally, the PCB is placed in an aluminum enclosure to provide RF shielding.

### Absorption profile

The IR absorption spectra of the metasurfaces incorporated on the MEMS resonators were measured using a Bruker INVENIO Fourier transform infrared (FTIR) spectrometer coupled with a Hyperion 2000 microscope. A bare Au surface was employed as a 100% reflection standard for background calibration. Reflection spectra (R) were acquired at 4 cm^−1^ resolution and averaged over 32 scans. Baseline correction was applied by subtracting a linear baseline over the spectral ranges. Absorption (A) was determined as A = 1 – R, under the assumption that no IR light passes through the metasurface due to the considerable thickness of the metal ground plane, which exceeds the penetration depth (tens of nanometers) of the IR light in gold.

We note that the integration of metasurfaces with RF MEMS to achieve IR spectral selectivity has been widely studied in the literature^21,23,24,30^. Alternative approaches to obtain spectral selectivity can be roughly divided into two main groups. On the one hand, bulky approaches based on interferometry that rely on moving arms coupled with broadband IR detectors^1,2^. On the other hand, optical techniques that provide spectral selectivity before the radiation reaches the detector—like gratings or optical filters—which can also be bulky and might require extreme tunability and careful alignment^32^.

### Responsivity

The responsivity of the device is evaluated using a custom electro-optical interrogation system (see Fig. 1a and Supplementary Information, Section 4). On the electronic side, the readout is a reflectometry-based approach composed of benchtop RF components. An RF tone generated by a Keysight E8663D source is split (Mini-Circuits RF splitter Z99SC-62-S +) into two signal paths. One signal path is fed into the input of an ADI AD8302 RF phase comparator and serves as a reference. The other signal path excites the IR detector. The reflected signal is collected by a circulator (RF-Lambda RFLC090M19M23), amplified using a low-noise amplifier (RF-Lambda R18M66MSA), and then fed into the second input of the RF phase comparator. The comparator outputs a DC signal linearly proportional to the difference in phase between the two RF signals with a transfer function of 10 mV/deg, constant for a wide range of RF signals from 5 to 2700 MHz. The DC output voltage in the absence of IR is taken as a baseline value, where any IR-induced shift away from baseline is taken as the voltage signal.

On the optical side, a coherent IR beam is generated by an optical parametric oscillator (OPO, EKSPLA PT277-XIR) with high output power, ultra-wide wavelength tunability, and automatic electronic control. The OPO beam shape was measured with an IR beam profiler (DataRay S-WCD-IR-BB-30) to determine the total IR power incident over the sensor area. The power of the incident IR is controlled using a linear polarizer mounted in a mechanical rotator, which is controlled electronically to achieve precise and automatic power control. Additional information about the setup is provided in Supplementary Information, Section 4.2.

To derive the detector’s responsivity (Fig. 2a), the output voltage versus incident IR power is recorded. The incident IR power is unmodulated, and a baseline measurement in the absence of IR light was taken. The difference between the baseline output voltage and the voltage with IR light is the reported voltage response and would represent the peak-to-peak value of the square wave produced if the incident IR light were modulated by a chopper. Equation (2) provides a linear fit through the linear portion of the measured data. The slope of this fit is taken as the responsivity. Data in degrees and voltage are simultaneously provided using the RF phase comparator’s transfer function.

The detector’s spectral selectivity is further determined (Fig. 2b) by scanning its response from 5 to 6.8 μm using the wavelength-tunable IR beams generated by the OPO. Power is kept sufficiently low such that the detector operates in its linear regime and is kept constant for each scanned wavelength. The measured response at each wavelength is normalized by the response at ${\lambda }_{0}=5.94\,{{{\rm{\mu }}}}{{{\rm{m}}}}$, and multiplied by the responsivity determined earlier. The time response of the IR detector (Fig. 2c) is determined by illuminating it with 10 μW of incident IR power that is modulated with an optical chopper. The now modulated output of the RF phase comparator is fed into an oscilloscope. The peak-to-peak voltage of the square wave output signal is recorded versus modulation frequency. The peak-to-peak voltage versus modulation frequency is normalized by the peak-to-peak voltage measured at 10 Hz and multiplied by the responsivity measured in Fig. 2a. The modulation frequency is swept from 10 Hz to 850 Hz, far below the 10 MHz cutoff frequency of the RF phase comparator’s output. Fig. 2d is characterized by chopping 40 μW of incident IR light at 100 Hz and connecting the RF phase comparator’s output to an oscilloscope.

We emphasize that our characterization setup does not rely on a blackbody radiator^21,23,24,28^. In set-ups based on blackbody radiators, the detected signal is proportional to the convolution of the device’s spectral responsivity with the blackbody source’s IR spectrum. As a result, such setups do not allow us to determine the device’s wavelength and power-dependent response, which are key figures of merit. Additionally, blackbody radiators generate IR beams with spot sizes that can be significantly larger than the device under test, cannot be easily focused, and might heat the detector substrate. Such heating is difficult to quantify and may mask the true response of the sensor. All these challenges are addressed in this work by using the experimental set-up described above, based on a widely tunable OPO IR laser.

Finally, we note that previous approaches employed frequency-shift measurements in micro- and nanomechanical resonators that are constrained both by resonator noise (*e.g.*, Brownian motion causing random phase and frequency fluctuations) and by the readout circuitry (amplifier and detector noise). This readout scheme imposes trade-offs between speed and precision: high loop bandwidth or fast update rates degrade frequency precision, whereas narrowing the bandwidth improves precision but slows response. ref. ^33^ provides a detailed analysis of conventional tracking schemes, including feedback-free drive, self-sustaining oscillators, and PLLs, showing that the frequency stability (often quantified via Allan deviation) is ultimately limited by the interplay of loop bandwidth, discriminator slope, and resonator noise. In this context, consider the frequency-shift responsivity of the RF MEMS employed here, 124 Hz/µW. Achieving an NEP of ~ $450\ {{{\rm{pW}}}}/\sqrt{{{{\rm{Hz}}}}}$ ^1, 2^ as measured in our reflectometric device, would require effectively resolving frequency shifts down to 0.056 Hz using an RF frequency of around 202 MHz in a readout that does not introduce any additional noise. This comparison highlights again that reflectometry is able to drastically enhance the IR responsivity of RF MEMS-based sensors, and that achieving such performance in RF MEMS-based IR sensors based on frequency tracking would require the use of ultra-precise and low-noise PLL circuits that might not be available at higher RF frequencies (*i.e.*, GHz or 100s of MHz).

### IR beam linewidth

The spectrum of the IR beam generated by the OPO (EKSPLA, PT277XIR) is measured by routing the beam into the inlet port of an FTIR tool (Bruker INVENIO FTIR coupled with a Hyperion 2000 microscope) and setting it as an external source. The beam characterization is performed in reflection mode using a gold mirror (Thorlabs, PF10-03-M02) as a reference, revealing a linewidth of 15.4 nm. Additional information about this characterization is provided in the Supplementary Information, Section 4.4.

### RF noise characterization

A Keysight E5052B signal source analyzer is employed to measure the phase noise of the RF signal that feeds the platform. For the reference path, the RF signal generated by a Keysight E8663D source is coupled directly to the source analyzer. For the sensing path, the RF signal generated by a Keysight E8663D is employed to excite the IR detector at its resonance frequency with an RF power level of −15 dBm ( ~ 40 mVrms). Note that the absolute maximum input RF voltage, *V*_0_, that can be sent to the MEMS before permanent damage varies from device to device but can be roughly stated to be >+10 dBm ( ~ 700 mVrms). The onset of nonlinear responses also varies by device and depends on the quality factor, but can be qualitatively stated to be ~−5 dBm ( ~ 126 mVrms), see refs. ^57,58^. Before being fed into the noise analyzer, the signal reflected from the MEMS is amplified with a low-noise amplifier, which introduces negligible phase noise to the signal. The resolution bandwidth of the phase noise analyzer is set to be 24.4 Hz. Additional information about this characterization is provided in the Supplementary Information, Section 4.5.

### Output impedance and current

The output impedance of the AD8302 RF phase comparator was obtained by measuring the open-circuit DC output voltage and the output voltage with known loads. The expression ${{{{\rm{R}}}}}_{{{{\rm{out}}}}}={{{{\rm{R}}}}}_{{{{\rm{load}}}}}\frac{{{{{\rm{V}}}}}_{{{{\rm{OC}}}}-}{{{{\rm{V}}}}}_{{{{\rm{load}}}}}}{{{{{\rm{V}}}}}_{{{{\rm{load}}}}}}$, where V_OC_, V_load_ and R_load_ are the open-circuit and load voltages and the load resistance, respectively, is employed to determine an output resistance of ${{{{\rm{R}}}}}_{{{{\rm{out}}}}}\approx \,56.5\,\Omega$. An ammeter is then employed to verify that the current generated by the RF phase comparator follows ${{{{\rm{i}}}}}_{{{{\rm{out}}}}}\left({{{\rm{t}}}}\right)=\frac{{{{\rm{v}}}}\left({{{\rm{t}}}}\right)}{{{{{\rm{R}}}}}_{{{{\rm{out}}}}}}.$

### Voltage noise spectral density

To determine the baseline noise floor of the platform, ${{{{\rm{v}}}}}_{{{{\rm{n}}}},{{{\rm{sys}}}}}\left({{{\rm{\omega }}}}\right)\approx {{{{\rm{v}}}}}_{{{{\rm{n}}}},{{{\rm{PC}}}}}\left({{{\rm{\omega }}}}\right)$, a manual control step attenuator (RF-Lambda RKT2G2K10) is employed in the platform instead of the RF MEMS. A schematic of the setup is provided in the Supplementary Information, Section 2.4. The output of the RF phase comparator is fed into a lock-in amplifier (Zurich Instruments MFLI 5 MHz). The lock-in amplifier’s internal oscillator is swept from 10 Hz to 550 Hz to measure the noise signal present at each frequency component (Fig. 4a–black solid lines). The attenuator noise experiment was performed for a return loss of −5 dB and −22.5 dB, with no difference in noise between the two cases. Then, the attenuator is replaced by the RF MEMS coupled with a matching network. The experiment is then repeated for all values of the detector’s phase-slope quality factor considered in this work, leading to the voltage noise spectral density values shown in Fig. 4a (colored solid lines). Our experiments confirm that the voltage noise spectral density of the platform is determined by the flicker noise added by the MEMS to the RF signal that is reflected from the device. This is further proven in the Supplementary Information, Section 2.4, where the voltage noise spectral density of the system is explored experimentally when noisy RF sources are considered.

### Noise equivalent power

NEP calculations reported in this paper relate noise and IR responsivity and provide the predicted optical power of the unmodulated IR beam per square root Hertz. In terms of noise, the Keysight E5052B source analyzer measures the power spectral density of the RF tone phase fluctuations in dBc/Hz. This metric is then converted to root-mean-square (rms) phase deviation per square root Hertz, ${{{{\rm{\phi }}}}}_{{{{\rm{n}}}}}\left({{{\rm{\omega }}}}\right)\left[{{{\rm{rad}}}}\cdot {{{{\rm{Hz}}}}}^{-1/2}\right],$ as described in ref. ^36^. Similarly, the voltage noise spectral density ${{{{\rm{v}}}}}_{{{{\rm{n}}}},{{{\rm{sys}}}}}\left({{{\rm{\omega }}}}\right)\left[{{{\rm{V}}}}\cdot {{{{\rm{Hz}}}}}^{-1/2}\right]$ is determined by the lock-in amplifier as an rms quantity^40^.

In terms of IR responsivity, peak-to-peak values were determined in the time domain in Fig. 2c when the detector is excited by an IR beam modulated with a mechanical chopper spinning at frequency ω, *i.e.*, by a train of IR pulses. For the NEP to predict the power of the unmodulated IR beam, the peak-to-peak responsivity related to a train of pulses in the time domain is converted to the responsivity of a tone at the modulation frequency ω (*i.e.*, the amplitude of the first harmonic of the Fourier expansion of a square wave modulation) through a weight factor ${{{{\rm{c}}}}}_{1}=\frac{\sqrt{2}}{\pi }$. This operation is equivalent to accurately measuring noise and responsivity using an approach that provides record responses in frequency (like a phase-sensitive lock-in technique) and permits us to compare NEP metrics with the power level of the weakest discernible IR signal that can be resolved in the platform for a given bandwidth (detection limit), as described below.

### SNR and detection limit

The detection limit of the proposed reflectometric platform is characterized by feeding the output of the RF phase comparator into a lock-in amplifier (Zurich Instruments MFLI 5 MHz), which is locked to the operation frequency of an optical chopper modulating incoming IR light. The modulation frequency, set by the chopper, is swept from 20 to 550 Hz. The bandwidth of the lock-in is set to 0.783 Hz. The noise floor for a given modulation frequency is determined by recording the mean voltage output plus the standard deviation in the absence of IR light. Detector response is recorded at various IR powers using a power control system based on IR polarizers (Supplementary Information, Section 4) together with combinations of 3 dB, 10 dB, 20 dB, and 30 dB IR neutral density filters to bring incident IR power levels down to very small values. Incident IR light is weakened until the signal level is equal to the noise level. A plot of ${{{\rm{SNR}}}}\left[{{{\rm{dB}}}}\right]=10{\log }_{10}\frac{{{{\rm{signal}}}}}{{{{\rm{noise}}}}}$ is created with measurement data and a linear fit through the data (inset of Fig. 4c). The point giving an SNR of 0 dB is extrapolated and reported as the minimum power (Fig. 4c). During the experiment, an SNR around 1 dB is measured to maximize accuracy in the determination of the 0 dB point. This approach follows recent guidelines provided to accurately characterize IR sensors^5^.

## Supplementary information

Supplementary Information

Transparent Peer Review file

## Data Availability

All source data used in this study are available in the link below 10.6084/m9.figshare.32067705.
