# Supplementary material for: High responsivity IR sensing based on reflectometric RF MEMS
Source: Nat Commun. 2026 Jun 16;17:7625. doi: 10.1038/s41467-026-74152-3 (PMC13429729; doi:10.1038/s41467-026-74152-3)
Supplement: Supplementary file 1 — Supplementary Information [file 41467_2026_74152_MOESM1_ESM.pdf]

Supplementary Information: High Responsivity IR Sensing based on Reflectometric RF-MEMS

Melisa E. Gülseren[1*], Matthew Benson[1*], Zhixing Lin (林志星)[1*], Tianyou Li (李天佑)[1], William Putnam[1], and J. Sebastian Gomez-Diaz[1#]

[1]Department of Electrical and Computer Engineering, University of California, Davis, Davis CA, USA

*These authors contributed equally: M. E. Gulseren, Matthew Benson, Zhixing Lin

[#] Correspondence: J. S. Gomez-Diaz (jsgomez@ucdavis.edu)

Table of Contents

1. Reflectometric RF-MEMS IR detectors 3

1.1 RF-MEMS IR sensors 3

1.2 Temperature coefficient of frequency 5

1.3 IR metasurface 6

1.4 Matching network 7

1.5 RF phase comparator 7

2. Analysis of reflectometric RF-MEMS IR detectors 8

2.1 Responsivity 8

2.1.1 Responsivity in phase 8

2.1.2 Responsivity in amplitude 10

2.1.3 Linearity and saturation 11

2.2 Phase noise in reflectometric RF MEMS based IR detectors 11

2.2.1 Flicker noise 11

2.2.2 Phase noise due to thermal fluctuations 13

2.3 Noise equivalent power 13

2.3.1 Fundamental mechanisms 13

2.3.2 Frequency-shift versus reflectometric RF MEMS-based IR detectors 14

2.4 Differential reflectometric system 15

2.4.1 Baseline voltage noise spectral density 16

2.4.2 Noise equivalent power 16

2.4.3 Noise equivalent power: predictions 17

2.4.4 Influence of the RF tone phase noise 18

3. Numerical simulations 20

3.1 Multi-physics numerical simulations 20

3.2 Circuit simulations 21
4. Experimental characterization 23
4.1 Experimental set-up: Overview 23
4.2 IR beam: power control 25
4.3 IR beam: spatial profile 25
4.4 IR beam: spectral bandwidth 27
4.5 Influence of power and LNA in the phase noise of RF signals 27
4.6 Power-independent absorption profile 28
5. References 29

1. Reflectometric RF-MEMS IR detectors

1.1 RF-MEMS IR sensors

The design and fabrication of the RF MEMS-based IR sensors employed in this work are described in Methods. Fig. S1 summarizes the fabrication process and provides additional information for all steps involved. Fig. S2 shows the measured admittance parameters of the RF MEMS described in the main paper and fits them with a Modified Butterworth-Van Dyke (MBVD) circuit model [1]. Fig. S3 plots the scattering parameters of the RF MEMS when it is connected to different matching networks, as described in Fig. 1a

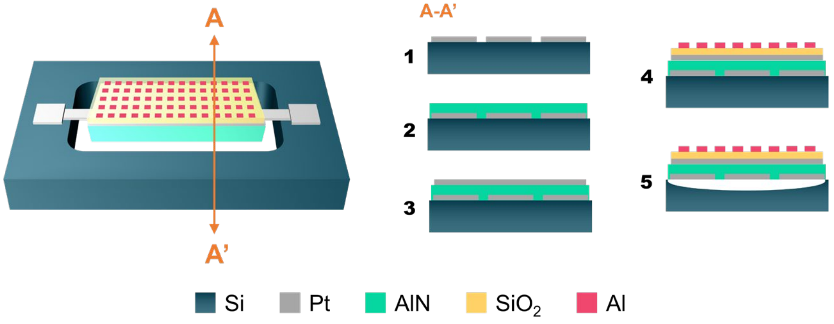

Figure S1. Microfabrication process of RF MEMS-based IR sensors (not to scale). The microfabrication process for the MEMS-IR sensor illustrated in the schematic on the left is shown along the width (A-A') of the device. The microfabrication sequence includes: (1) patterning and depositing a 100 nm platinum bottom electrode; (2) depositing a 1 um thick piezoelectric AlN layer, followed by patterning and dry etching to open vias to the bottom electrode (not shown); (3) patterning and depositing a 100 nm platinum top electrode, which also serves as the metasurface ground plane; (4) depositing a 200 nm SiO_2 layer; depositing, patterning, and etching a 100 nm aluminum layer to form the metasurface nanostructures; patterning and etching the SiO_2 to create vias to the top electrode, and patterning and etching the SiO_2 and AlN layers to define the resonator geometry (not shown); and (5) performing an isotropic etch using xenon difluoride to release the resonators from the substrate.

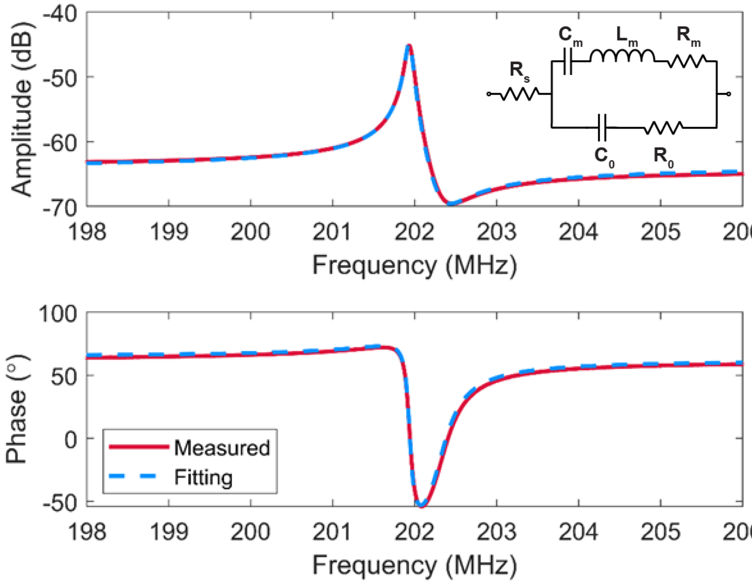

Figure S2. Measured and fitted admittance response in magnitude (top) and phase (bottom) for the RF MEMS described in the main paper. The fitting MBVD circuit parameters are: R_s = 10 Ω, R_0 = 697.68 Ω, C_0 = 558.62 fF, R_m = 173.00 Ω, C_m = 2.00 fF, and L_m = 310.85 μH. Inset shows the equivalent MBVD circuit employed to fit the MEMS response.

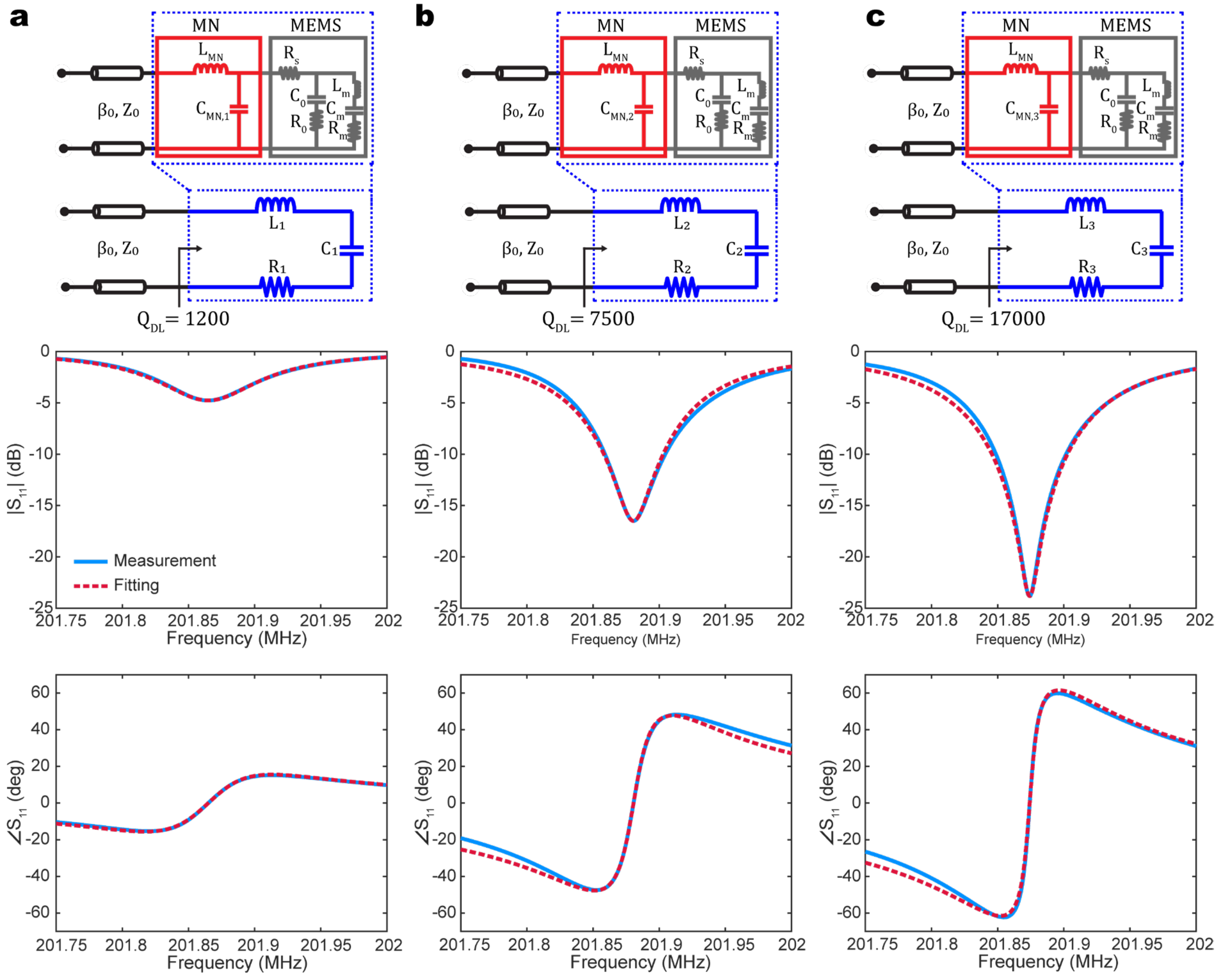

Figure S3. Measured (solid blue lines) and fitted (dashed red lines) scattering parameters of the reflectometric IR detector composed of a RF MEMS resonator connected to a $Z_0 = 50\ \Omega$ transmission line via a matching network. Top row depicts the three different matching networks considered here, each one leading to a different phase-slope quality factor of the device. **a.** Phase-slope quality factor of $Q_{DL} = 1168\ (\sim 1200)$. Equivalent circuit parameters are $R_1 = 186.5\ \Omega$, $L_1 = 297.3\ \mu\text{H}$, and $C_1 = 2.0908\ \text{fF}$. **b.** Phase-slope quality factor of $Q_{DL} = 7517\ (\sim 7500)$. Equivalent circuit parameters are $R_2 = 67.58\ \Omega$, $L_2 = 122.51\ \mu\text{H}$, and $C_2 = 5.0733\ \text{fF}$. **c.** Phase-slope quality factor of $Q_{DL} = 16775\ (\sim 17000)$. Equivalent circuit parameters are $R_3 = 56.89\ \Omega$, $L_3 = 97.365\ \mu\text{H}$, and $C_3 = 6.3837\ \text{fF}$.

of the main paper. Measured results are fitted by modelling the reflectometric device as a simple RLC network, valid around resonance, as described in the main text.

The unloaded quality factor of the RF MEMS is $Q_{MU} \approx 2{,}200$ and has been calculated using the MBVD equivalent circuit extracted from the admittance parameters (Fig. S2) as described in the literature [1]. The phase-slope quality factors of the device (i.e., RF MEMS + matching network) have been determined to be $Q_{DL} \approx 1200$, 7500, and 17000 for different capacitance values. An analytical expression can be obtained to describe the phase-slope quality factor of the device. To this purpose, we start from the definition of quality factor [2]

$$Q_{DL} = \frac{f}{2}\frac{d\varphi}{df}, \tag{S1}$$

where f is the network operation frequency, and φ is the phase of the reflection coefficient. For the specific case of the RLC network shown on Fig. 1a (main paper), the reflection coefficient can be expressed as

$$\Gamma = \frac{Z_{in} - Z_0}{Z_{in} + Z_0} = \cdots = \frac{(1 - 4\pi^2 f^2 LC)^2 + 4\pi^2 f^2 C^2[(R + Z_0)(R - Z_0)] - j4\pi fCR_0(1 - 4\pi^2 f^2 LC)}{(1 - 4\pi^2 f^2 LC)^2 + 4\pi^2 f^2 C^2 (R + Z_0)^2}, \tag{S2}$$

where $Z_{in} = R + j2\pi fL + \frac{1}{j2\pi fC}$ represents the input impedance of the entire IR detector. The phase of the reflection coefficient can be obtained as

$$\varphi = \measuredangle\Gamma = \tan^{-1}\left(\frac{-4\pi fCZ_0(1 - 4\pi^2 f^2 LC)}{(1 - 4\pi^2 f^2 LC)^2 + 4\pi^2 f^2 C^2 (R + Z_0)(R - Z_0)}\right) \approx \frac{-2Z_0(1 - 4\pi^2 f^2 LC)}{2\pi fC(R + Z_0)(R - Z_0)}. \tag{S3}$$

In this last expression and in the following, we assume an operation frequency near resonance $\left(f \to f_o = \frac{1}{2\pi\sqrt{LC}}\right)$ and the small angle approximation. The variation of the phase versus frequency is then given by

$$\frac{d\varphi}{df} = \frac{8\pi Z_0 L}{(R + Z_0)(R - Z_0)}, \tag{S4}$$

which permits us to derive the phase-slope quality factor of the RLC network at resonance as

$$Q_{DL} = \frac{f_0}{2}\frac{d\varphi}{df} = \frac{4\pi f_0 Z_0 L}{(R + Z_0)(R - Z_0)}. \tag{S5}$$

The RLC model of the device can then be extracted by first using Eq. (S2) at resonance to find the value of R from Z_o and S_{11}. Then, the variation of phase versus frequency at resonance is extracted and Eq. (S4) is used to determine L. From here, C is obtained from $f_o = \frac{1}{2\pi\sqrt{LC}}$.

1.2 Temperature coefficient of frequency

The temperature coefficient of frequency (TCF) of RF MEMS resonators refers to the rate at which the resonance frequency shifts with temperature [3]. Aluminum nitride (AlN) typically exhibits a negative TCF, around −20 to −30 ppm/K, due to the combined effects of thermal expansion and temperature-dependent changes in its elastic constants [1]. In common RF applications, such frequency drift can impact the resonator stability and thus temperature compensation techniques have been developed to minimize TCF. In the context of RF MEMS-based IR sensors, it is desirable to have a TCF as high as possible as this results in larger responsivity in the detection process. The TCF is defined as [3]

$$TCF = \frac{1}{f_0}\frac{df_0}{dT} \quad [ppm/K], \tag{S6}$$

where f_0 is the device's resonance frequency and T is temperature. TCF can be determined by measuring the device response versus temperature and tracking the shift of its resonance frequency. To this purpose,

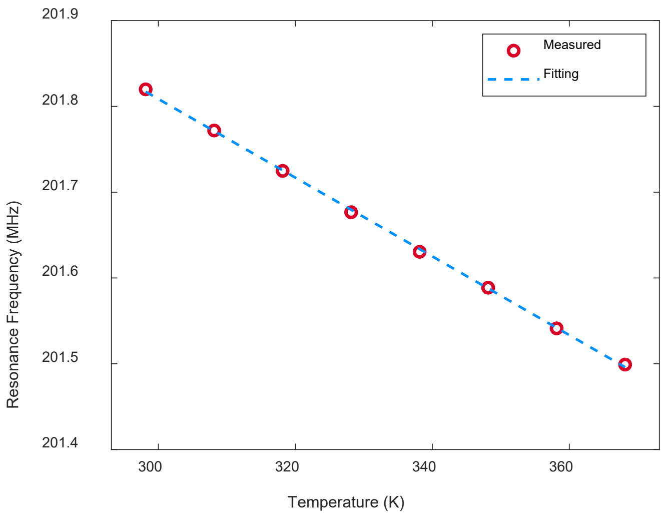

Figure S4. RF MEMS resonance frequency versus temperature. The frequency of the resonator's series resonance at increasing temperatures is extracted from Y-parameter measurements carried out on the MEMS resonator [3]. This measurement permits to extract the device TCF following Eq. (S6).

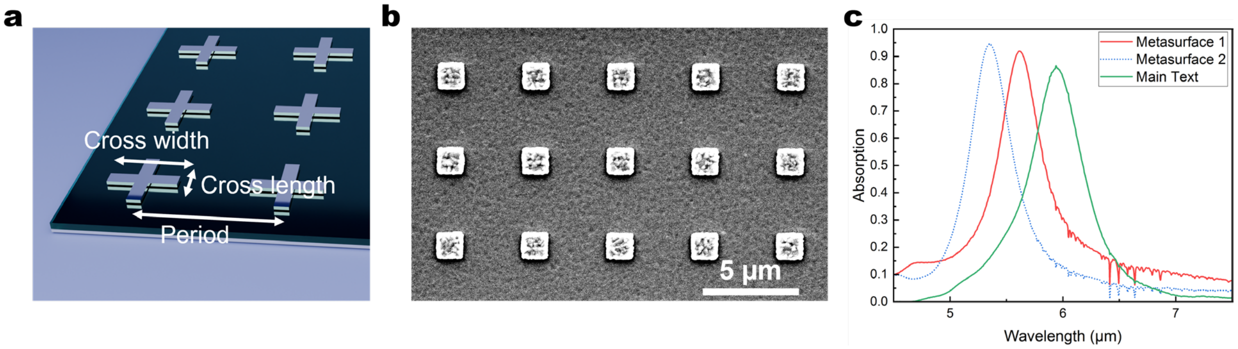

Figure S5. IR metasurface decorating the RF-MEMS resonator. (a) Schematic. (b) SEM of the metasurface employed in the main paper. (c) Measured IR absorption spectrum from the device described in the main text. Two additional devices decorated with metasurfaces composed of a slightly different unit cell designs are also measured and characterized to demonstrate how the targeted IR absorption wavelength can be manipulated through design parameters.

the device is placed in a temperature-controlled Lakeshore RF probe station and is characterized versus temperature. Measured results are shown in Fig. S4, revealing a TCF of -22.73 ppm/K.

1.3 IR metasurface

As described in Methods, the metasurface that defines the IR absorption profile of the sensor is composed of a metal-insulator-metal structure in which the top electrode metal layer of the MEMS resonator acts as the ground metal layer (Fig. S5a). The metasurface decorating the device employed in the main text is composed of patch-shaped nanostructures with size of 1.87 µm and periodicity of 4.5 µm (Fig. S5b). The shape of the nanostructure can easily be varied to control absorption metrics such as wavelength, full width half maximum (FWHM), field of view, polarization dependence, etc. following standard metasurfaces procedures [4]. The absorption profile measured using FTIR for the device

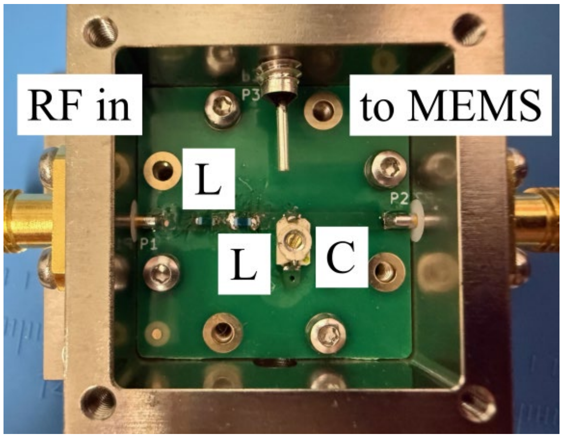

Fig. S6. Matching network. This network is employed in the main paper to control the phase-slope quality factor of the sensor. The shielded network contains two series inductors (L), and one shunt and mechanically variable capacitor (C). RF signals fed the matching network from the "RF in" port; the output port, "to MEMS" port, is connected to the MEMS sensor via a GSG probe.

employed in the main text (Fig. 2b) is duplicated here in Fig. S5c together with two different metasurface designs targeting slightly shifted central absorption wavelengths, evidencing that the detection wavelength can be controlled through the design geometry. Metasurface 1 is composed of a cross shaped nanostructure with cross width of 450 nm, cross length of 2.23 µm, and periodicity of 3.9 µm, resulting in a peak absorption of 92% at wavelength of 5.61 µm, with FWHM of 475 nm. Metasurface 2 is composed of a cross shaped nanostructure with cross width of 350 nm, cross length of 2.17 µm, and periodicity of 3.5 µm, resulting in a peak absorption of 95% at wavelength of 5.35 µm, with FWHM of 450 nm.

1.4 Matching network

The matching network employed in our set-up is described in Methods and depicted in Fig. S6. Power enters from the "RF in" port. The output "to MEMS" port is connected to the GSG probe which interfaces with the RF MEMS resonator.

1.5 RF phase comparator

The RF phase comparator used is a Valon Model 4002 [5], which is a packaged phase/gain detector based on an Analog Devices AD8302 chip [6]. The comparator takes the output difference between two logarithmic amplifiers to provide phase comparison over a wide range of RF frequencies and input powers. The amplitude and phase response of the RF comparator is shown in Fig. S7. The phase port provides an output voltage proportional to the relative phase between two RF input signals, with a transfer function of 10 mV/degree and -10 mV/degree for the ranges of -180° and 0° and between 0° and 180° respectively. The packaged RF comparator has a 30 MHz bandwidth, and a constant phase output for RF signals ranging from 5 MHz to 2700 MHz [5] over a wide range of RF powers.

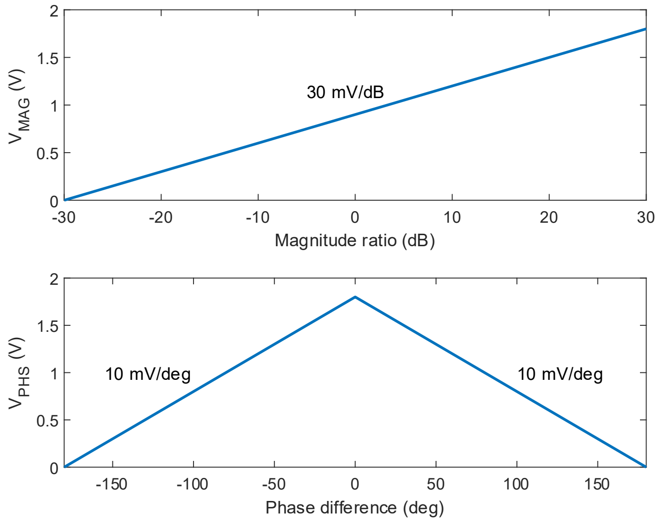

Fig. S7. Response of the RF phase comparator Analog Devices AD8302 in magnitude and phase. Output voltage of the RF comparator versus the amplitude/phase (top/bottom) difference of two RF tones oscillating at the same frequency.

2. Analysis of reflectometric RF-MEMS IR detectors

2.1 Responsivity

2.1.1 Responsivity in phase

Upon absorbing IR radiation, the temperature of the RF MEMS-based detector increases following [7]

$$\Delta T(\omega,\lambda) = \frac{\eta(\lambda)P_{IR}(\lambda)}{\sqrt{G_{th}^2 + \omega^2 C_{th}^2}} = \frac{\eta(\lambda)P_{IR}(\lambda)R_{th}}{\sqrt{1 + \omega^2 C_{th}^2 R_{th}^2}} = \eta(\lambda) \cdot \chi(\omega) \cdot P_{IR}(\lambda) \cdot R_{th} \quad [K], \tag{S7}$$

where $\eta(\lambda)$ is the wavelength dependent absorptance of the RF MEMS, R_{th} and C_{th} are the MEMS thermal resistance and capacitance, respectively, $\chi(\omega) = \left(1 + \omega^2 C_{th}^2 R_{th}^2\right)^{-\frac{1}{2}}$ accounts for the device time response versus the modulation frequency ω of the incoming IR beam, and $P_{IR}(\lambda)$ denotes the total IR power that is incident onto the device. $P_{IR}(\lambda)$ is calculated as the spatially varying beam power density integrated over the sensing area (140 μm x 60 μm in the device shown in Fig. 1c of the main paper). The temperature change in the AlN layer induces a shift in the device resonance frequency given by [8]

$$\Delta f(\omega,\lambda) = f_0 \cdot TCF \cdot \Delta T(\omega,\lambda) \quad [Hz], \tag{S8}$$

where f_0 is the device resonance frequency. Responsivity upon IR illumination can be defined as the ratio between the frequency shift over the IR beam power as [9]

$$R_f(\omega,\lambda) = \frac{\Delta f(\omega,\lambda)}{P_{in}(\lambda)} = f_0 \cdot TCF \cdot \eta(\lambda) \cdot \chi(\omega) \cdot R_{th} \quad [Hz/W]. \tag{S9}$$

The proposed reflectometric IR detectors rely on determining the relative phase variation that the IR beam induces on an RF tone reflected from the device. To maximize such variation, and as illustrated in Fig. 1 of the main paper and discussed above, the IR detector is composed of an RF MEMS coupled to an electrical matching network. This configuration can be modelled using a one port electrical network composed of an LC matching network connected to the MBVD model of the MEMS resonator (see top of Fig. S3). Around resonance, this electrical network can be further simplified using a simple equivalent RLC circuit (see top row of Fig. S3). The input impedance of the RLC network can be expressed as

$$Z_{in} = R + j\frac{(2\pi f)^2 LC - 1}{2\pi f C} \quad [\Omega], \tag{S10}$$

where f and $f_0 = \frac{1}{2\pi\sqrt{LC}}$ are the frequency of the incoming RF tone and the device resonant frequency, respectively. Assuming operation close to resonance ($f \rightarrow f_0$), a change in the resonant frequency f_0 can be mapped as a variation in the equivalent capacitance as $\Delta f = -\frac{f_0}{2C}\Delta C$ [8]. Combining this last expression with Eq. (S8) permits us to obtain the change in the equivalent capacitance due to temperature increase as $C_{\Delta T}(\omega,\lambda) = C\big(1 - 2 \cdot TCF \cdot \Delta T(\omega,\lambda)\big)$. The device input impedance can be then approximated as

$$Z_{in,\Delta T}(\omega,\lambda) \approx R + j2\pi f_0 L\frac{\Delta C(\omega,\lambda)}{C} = R - j4\pi f_0 TCF \cdot \Delta T(\omega,\lambda) \quad [\Omega], \tag{S11}$$

where a small relative variation of the device capacitance has been assumed, i.e., $C \gg \Delta C(\omega,\lambda)$. The device reflection coefficient is

$$\Gamma(\omega,\lambda) = \frac{Z_{in,\Delta T}(\omega,\lambda) - Z_0}{Z_{in,\Delta T}(\omega,\lambda) + Z_0} = \frac{(R - Z_0) - j4\pi f_0 L \cdot TCF \cdot \Delta T(\omega,\lambda)}{(R + Z_0) - j4\pi f_0 L \cdot TCF \cdot \Delta T(\omega,\lambda)}, \tag{S12}$$

where Z_0 is the impedance of the electrical network connected to the device. Multiplying by a complex conjugate, this expression can be expressed as

$$\Gamma(\omega,\lambda) = \frac{R^2 - Z_0^2 + \big(4\pi f_0 L \cdot TCF \cdot \Delta T(\omega,\lambda)\big)^2 - j8\pi f_0 \cdot L \cdot TCF \cdot \Delta T(\omega,\lambda) \cdot Z_0}{(R + Z_0)^2 + \big(4\pi f_0 L \cdot TCF \cdot \Delta T(\omega,\lambda)\big)^2}. \tag{S13}$$

The phase of an RF tone reflected from the device undergoes a relative variation that depends on the temperature change following

$$\measuredangle\Gamma(\omega,\lambda) = \tan^{-1}\left(\frac{8\pi f_0 \cdot L \cdot TCF \cdot \Delta T(\omega,\lambda) \cdot Z_0}{R^2 - Z_0^2 + \big(4\pi f_0 L \cdot TCF \cdot \Delta T(\omega,\lambda)\big)^2}\right) \quad [rad]. \tag{S14}$$

Assuming that $\Delta T(\omega,\lambda) \rightarrow 0$, a small angle approximation can be employed to determine the linear phase variation versus temperature as

$$\measuredangle\Gamma(\omega,\lambda) \approx \frac{8\pi f_0 \cdot L \cdot TCF \cdot \Delta T(\omega,\lambda) \cdot Z_0}{R^2 - Z_0^2} \;\; [rad]. \tag{S15}$$

Next, the definition of phase-slope quality factor from Eq. (S5) is employed to arrive to

$$\measuredangle\Gamma(\omega,\lambda) = 2 \cdot Q_{DL} \cdot TCF \cdot \Delta T(\omega,\lambda) \;\; [rad]. \tag{S16}$$

Combining this last equation with Eq. (S7) allows us to analytically obtain the phase variation of the RF tone versus the power and wavelength of the incoming IR beam as

$$\measuredangle\Gamma(\omega,\lambda) = 2 \cdot Q_{DL} \cdot TCF \cdot \eta(\lambda) \cdot \chi(\omega) \cdot P_{IR}(\lambda) \cdot R_{th} \quad [rad]. \tag{S17}$$

The detector's responsivity in phase, defined as the phase change induced on the RF tone reflected from the device versus the power of the IR beam, can be expressed as

$$R_{\Delta\measuredangle}(\omega,\lambda) = \frac{\measuredangle\Gamma(\omega,\lambda)}{P_{IR}(\lambda)} = 2 \cdot Q_{DL} \cdot TCF \cdot \eta(\lambda) \cdot \chi(\omega) \cdot R_{th} \;\; [rad/W]. \tag{S18}$$

In practice, responsivity can be measured by monitoring the phase variation of the RF tone reflected from the IR detector versus the phase of a reference signal – easily implemented using an RF phase comparator. Then, responsivity yields

$$R_v(\omega,\lambda) = 2 \cdot H \cdot \eta(\lambda) \cdot \chi(\omega) \cdot TCF \cdot R_{th} \cdot Q_{DL} \; [V/W], \tag{S19}$$

which is the main result from this section. In this expression, H denotes the transfer function of the phase comparator and is given in V/rad units as shown in Fig. S7. This responsivity describes the full voltage swing that is produced upon IR light illumination or the peak-to-peak voltage of the output voltage square wave when the incident IR light is modulated by an optical chopper.

2.1.2 Responsivity in amplitude

Reflectometric RF MEMS-based IR detectors can also be configured to monitor amplitude differences between the two RF signals. Starting from Eq. (S13), the magnitude of an RF tone exciting the MEMS undergoes a relative variation that depends on the temperature change following

$$|\Gamma(\omega,\lambda)| = \frac{\sqrt{\left[R^2 - Z_0^2 + \left(4\pi f_0 L \cdot TCF \cdot \Delta T(\omega,\lambda)\right)^2\right]^2 + \left(8\pi f_0 \cdot L \cdot TCF \cdot \Delta T(\omega,\lambda) \cdot Z_0\right)^2}}{(R + Z_0)^2 + \left(4\pi f_0 L \cdot TCF \cdot \Delta T(\omega,\lambda)\right)^2}. \tag{S20}$$

Considering small temperature variations (i.e., $\Delta T(\omega,\lambda) \rightarrow 0$), this last expression can be approximated as

$$|\Gamma(\omega,\lambda)| \approx \frac{R - Z_0}{R + Z_0}\left[1 + \left(2Q_{DL} TCF \cdot \Delta T(\omega,\lambda)\right)^2\right]. \tag{S21}$$

The relative amplitude variation with respect to the reference RF tone is then given by

$$\Delta|\Gamma(\omega,\lambda)| = 2\Gamma_0\left(Q_{DL}\cdot TCF\cdot \Delta T(\omega,\lambda)\right)^2 = 2\Gamma_0\left(Q_{DL}\cdot TCF\cdot \eta(\lambda)\cdot \chi(\omega)\cdot R_{th}\cdot P_{IR}(\lambda)\right)^2, \quad \text{(S22)}$$

where $\Gamma_0 = \frac{R-Z_0}{R+Z_0}$ is the reflection coefficient at resonance without temperature variations. Eq. (S22) reveals that variations in magnitude are not linear with respect to the incident IR power. Additionally, the overall responsivity in amplitude is smaller than the one in phase. For these two reasons, the proposed reflectometric IR platform operates in phase instead of amplitude.

2.1.3 Linearity and saturation

The phase variation of an RF tone reflected from the IR detector versus a temperature change is given in Eq. (S14). As described in Eqs. (S15-S17), phase changes are linearly proportional to small temperature changes and incoming IR power. The linear regime of the proposed detector can be determined by examining the limit of the small angle approximation employed to derive Eq. (S17). This permits us to obtain the maximum IR power that can be detected in the linear regime as

$$2\cdot Q_{DL}\cdot TCF\cdot \eta(\lambda)\cdot \chi(\omega)\cdot P_{IR}(\lambda)\cdot R_{th} \approx 0.1 \;\rightarrow P_{max-linear}(\lambda) = \frac{0.05}{Q_{DL}\cdot TCF\cdot \eta(\lambda)\cdot \chi(\omega)\cdot R_{th}}\ [\mathrm{W}], \quad \text{(S23)}$$

where 0.1 rad has been considered as the limit in which the small angle approximation breaks.

Additionally, as illustrated in Fig. 2a (main paper) and Fig. S12, the sensor enters a nonlinear operation regime as the IR power goes beyond $P_{max-linear}(\lambda)$. Power saturation occurs within this nonlinear regime, and it can be determined numerically for a given detector using numerical simulations as described below in the circuit simulations subsection.

2.2 Phase noise in reflectometric RF MEMS based IR detectors

The phase noise of the RF signal reflected from the IR detector can be expressed as

$$\phi_n^2(\omega) = \phi_{RF}^2(\omega) + \phi_{flicker}^2(\omega) + \phi_{thermal}^2(\omega) \quad [\mathrm{rad}^2/\mathrm{Hz}], \quad \text{(S24)}$$

where $\phi_{RF}(\omega)$ is the intrinsic phase noise of the RF signal that excites the device and depends on the RF source employed; $\phi_{flicker}(\omega)$ is the 1/f flicker noise added by the RF MEMS; and $\phi_{thermal}(\omega)$ is the phase noise added by background thermal fluctuations to the reflected RF signal. Other contributions are negligible with respect to the phase noise from the RF signal.

2.2.1 Flicker noise

Flicker (or 1/f) noise is a fundamental noise contribution that appears in any vibrating mechanical structure [10]. In the case of AlN CMR resonators, this noise component roughly scales proportional to $1/Q_{MU}^4$ where Q_{MU} is the device unloaded quality factor [11]. Additionally, the 1/f noise depends on the specific resonator geometry, composing materials and thermal properties such as thermal diffusivities and

expansion coefficients [11]. The origin and underlying mechanisms of flicker noise are still not fully understood, and there are no closed-form expressions able to accurately capture its response. Through extensive parametric experiments [10, 11], it has been determined that this noise component is mostly independent of the power of the carrier tone and can be simply modelled as $\phi^2_{\text{flicker MEMS}} = \frac{b_0}{f}$, where b_0 is a constant that depends on the specific device.

In the proposed reflectometric platform, flicker noise becomes dominant with respect to other noise components for devices with a high phase-slope quality factor Q_{DL}. This behavior appears due to the interplay between the amplitude of the reflected RF signal, which decreases as Q_{DL} increases, and the flicker noise originating within the RF MEMS, which maintains a constant power level independently of the power of the incident RF tone. To model this scenario, we consider first the tone reflected from the MEMS in the absence of any noise components. When the RF tone excites the device at its resonance frequency, the reflected signal can be approximated as

$$v(t) = V_0 \cdot |\Gamma| \cdot \cos(2\pi f_0 t + \varphi) = V_0 \frac{Q_{DU}}{Q_{DL}} \frac{2R}{R_0 (R + Z_0)^2} \cos(2\pi f_0 t + \varphi) \quad [V], \tag{S25}$$

where V_0 is a constant that depends on the power of the incident RF signal, and the following identities for the reflection coefficient of the network operating around resonance have been employed

$$\Gamma = \frac{R - Z_0}{R + Z_0} = \frac{2\omega_0 Z_0 L}{Q_{DL}(R + Z_0)^2} = \frac{Q_{DU}}{Q_{DL}} \frac{2RZ_0}{(R + Z_0)^2}, \tag{S26}$$

with $Q_{DU} = \frac{\omega_0 L}{R}$. Next, we consider that the MEMS resonator generates a narrowband noise with a power spectral density of $\frac{b_0}{f}$ [W/Hz] at a frequency $f_0 + f$. Following the approach described in Ref. [10], this noise power density translates into phase variations of the reflected signal (i.e., phase noise) as

$$\varphi(t) = \frac{B}{V_0 \cdot |\Gamma|} \sqrt{\frac{b_0}{f}} \sin(2\pi f t) \quad [\text{rad}], \tag{S27}$$

where B is the noise bandwidth. Extending this approach for all frequencies, and dividing by $\sqrt{B}$ [10], the power spectrum density of the phase $\varphi(t)$ can be expressed as

$$S_\varphi(\omega) = \phi^2_{\text{flicker}}(\omega) = \frac{1}{V_0^2 \cdot |\Gamma|^2} \frac{b_0}{f} = \frac{1}{V_0^2} \frac{Q_{DL}^2 (R + Z_0)^4}{Q_{DU}^2 \cdot 4 Z_0^2 R^2} \frac{b_0}{f} \quad [\text{rad}^2/\text{Hz}]. \tag{S28}$$

Eq. (S28) analytically reveals that flicker noise of the reflected signal depends on the squared reflection coefficient of the device. $S_\varphi(\omega)$ can be converted to the more typically used units for phase noise of dBc/Hz as $10\log_{10}[\mathcal{L}(f)]$ where $\mathcal{L}(f) = \frac{1}{2}S_\varphi(f)$.

2.2.2 Phase noise due to thermal fluctuations

The spectral density of temperature fluctuations on the device at room temperature can be calculated analytically as [12]

$$\overline{\Delta T^2}(\omega) = \frac{4k_B T^2 R_{th}}{1 + \left(\frac{\omega}{\omega_c}\right)^2} \quad [K^2/Hz] \tag{S29}$$

where $\omega_c = \frac{G_{th}}{2\pi C_{th}}$ is the thermal cutoff frequency of the device. According to Eq. (S16), these thermal fluctuations translate to the phase of the reflected RF signal as

$$\overline{\Delta\measuredangle\Gamma^2}(\omega) = S_\phi(\omega) = \phi^2_{thermal}(\omega) = (2Q_{DL}TCF)^2 \cdot \overline{\Delta T^2}(f) = (2Q_{DL}TCF)^2 \frac{4k_B T^2 R_{th}}{1 + \left(\frac{\omega}{\omega_c}\right)^2} \ [rad^2/Hz], \tag{S30}$$

and constitute the phase noise added by the RF MEMS to the reflected RF signals. For all cases considered in this work, the phase noise due to thermal fluctuations is at least 10 dB lower than the measured phase noise of the RF reflected on the MEMS (Fig. 3a in the main paper). As a result, this component can safely be neglected.

2.3 Noise equivalent power

2.3.1 Fundamental mechanisms

The fundamental mechanisms that bound the noise equivalent power (NEP) of a MEMS-based thermal IR sensor have been extensively studied in the literature [7, 12, 13]. The lowest NEP attainable in such a sensor is given by

$$NEP_{min} = \sqrt{NEP^2_{thermal} + NEP^2_{rad} + NEP^2_{mech}} \quad [W/\sqrt{Hz}], \tag{S31}$$

where $NEP_{thermal}$, NEP_{rad}, NEP_{mech} refer to the NEP generated by different noise mechanisms, namely (i) thermal fluctuations; (ii) radiative heat exchange with the surrounding background; and (iii) thermomechanical noise generated by the motion of the MEMS, respectively. These components can be calculated as [7, 12, 13]

$$NEP_{thermal} = \frac{1}{\eta}\sqrt{4k_B T^2 R_{th}^{-1}} \quad [W/\sqrt{Hz}], \tag{S32}$$

$$NEP_{rad} = \sqrt{16A\epsilon\sigma k_B T^5} \quad [W/\sqrt{Hz}], \tag{S33}$$

$$NEP_{mech} = \frac{1}{R_{th} TCFQ_U}\sqrt{\frac{k_B T}{4P_c}} \quad [W/\sqrt{Hz}], \tag{S34}$$

where T is temperature; R_{th}, Q_u, A, η, and ϵ are the resonator thermal resistance, unloaded mechanical quality factor, area, absorption, and emissivity, respectively; k_B is the Boltzmann constant, σ is the Stefan-Boltzmann constant, and P_c is the power carried by the RF signal that excites the resonator in the linear regime.

Eq. (S31) approximates the minimum NEP potentially attainable in thermal sensors, but it does not aim to model the device NEP response versus modulation frequency. For the RF MEMS-based IR detector described in the main text, neglecting the presence of the flicker noise originating within the MEMS, a thermal resistance of $R_{th} = 31.4 \cdot 10^3\ K/W$ was experimentally measured leading to a $NEP_{min} \approx 12\ pW/\sqrt{Hz}$.

2.3.2 Frequency-shift versus reflectometric RF MEMS-based IR detectors

To date, RF MEMS-based IR sensors have relied on monitoring the frequency shift of the device resonant frequency upon IR illumination [9, 14-16]. The NEP in these devices is defined as

$$NEP = \frac{f_n}{R_f} \quad [W/\sqrt{Hz}], \tag{S35}$$

where f_n $[Hz/\sqrt{Hz}]$ is the frequency fluctuation induced noise, i.e., the frequency instability of the device at resonance, and R_f $[Hz/W]$ is the detector responsivity defined as the change of the resonant frequency versus the IR power that impinges onto the device. Even though NEP down to ~ 30 $pW/\sqrt{Hz}$ have been predicted [9, 14-16], there are no experiments confirming that such metrics can be attained in practice by measuring IR signals with decreasing power levels. Additionally, these results should be contextualized in the framework of accurate IR sensing characterization –see, for instance, Ref. [17]– which requires the NEP to depend on the modulation frequency of the incoming IR beam. For instance, in some cases, the fluctuation induced noise f_n is approximated at a single frequency offset by tracking the amplitude variations of the signal reflected from the MEMS versus time. A vector network analyzer (VNA) is employed to track the device frequency shift versus IR power and to obtain an estimated integrated noise that includes the VNA's ability to resolve frequencies and the phase noise of the generated RF tones. Similarly, the highest measured responsivity R_f is employed - thus neglecting how the responsivity changes versus the modulation frequency of the incoming beam [9]. Therefore, NEP associated with MEMS-based IR sensors are often calculated using fluctuation induced noise and responsivity values calculated at different

frequency offsets, leading to artificially small NEP values that do not represent the actual sensor response. In fact, the sensor NEP strongly depends on the modulation frequency of the incoming IR beam: continuous IR waves will lead to larger NEP values due to the influence of phase noise of the RF signal in the detection system at low offset frequencies, whereas IR beams modulated at high frequencies will lead to moderate NEP values due to the limited speed of thermal sensors. An optimal IR modulation frequency will lead to the lowest NEP, which appears as a trade-off between the high-pass filter-like noise response and low-pass responsivity responses. Accurately determining the NEP versus the modulation frequency of the IR beam is critical to assess the performance of IR sensors [17].

Considering the RF signal reflected from the IR detector, the noise equivalent power of the detection process is defined in the main paper as

$$\mathrm{NEP}(\omega,\lambda) = \frac{\phi_n(\omega)}{R_{\Delta\measuredangle}(\omega,\lambda)\cdot c_1} \quad [\mathrm{W}/\sqrt{\mathrm{Hz}}], \tag{S36}$$

with $c_1 = \frac{\sqrt{2}}{\pi}$ (see Methods), which adequately captures the device NEP with respect to the modulation frequency of the IR beam and the frequency-dependent phase noise of the detection system. Eq. (S36) relates noise and responsivity in the frequency domain, leading to power metrics that can be directly compared to IR power levels measured using phase-sensitive lock-in techniques. Additionally, Eq. (S36) reveals the importance of using low noise RF signals to excite the MEMS to achieve the lowest possible NEP. As further described below, such a stringent requirement is eliminated by the differential scheme employed in the proposed reflectometric platform.

2.4 Differential reflectometric system

The platform output voltage is provided by the phase detector AD8302 [6] (See Fig. S7), which implements a differential approach to eliminate common-mode noise. The voltage noise spectral density $v_{n,sys}(\omega)$ of the complete system can be modelled as

$$v_{n,sys}^2(\omega) = v_{n,PC}^2(\omega) + H^2\phi_{flicker}^2(\omega) \quad [\mathrm{V}^2/\mathrm{Hz}], \tag{S37}$$

where $v_{n,PC}(\omega)$ and H are the output voltage noise spectral density and transfer function of the phase comparator [6], respectively, and $\phi_{flicker}(\omega)$ is the flicker noise added by the RF MEMS as described in Eq. (S28). It should be stressed that the phase noise of the RF source is common-mode noise and is eliminated by the phase comparator. This remarkable feature greatly reduces the overall noise of the system and enables the use of standard and even relatively noisy RF sources on the proposed platform without deteriorating the overall sensing performance.

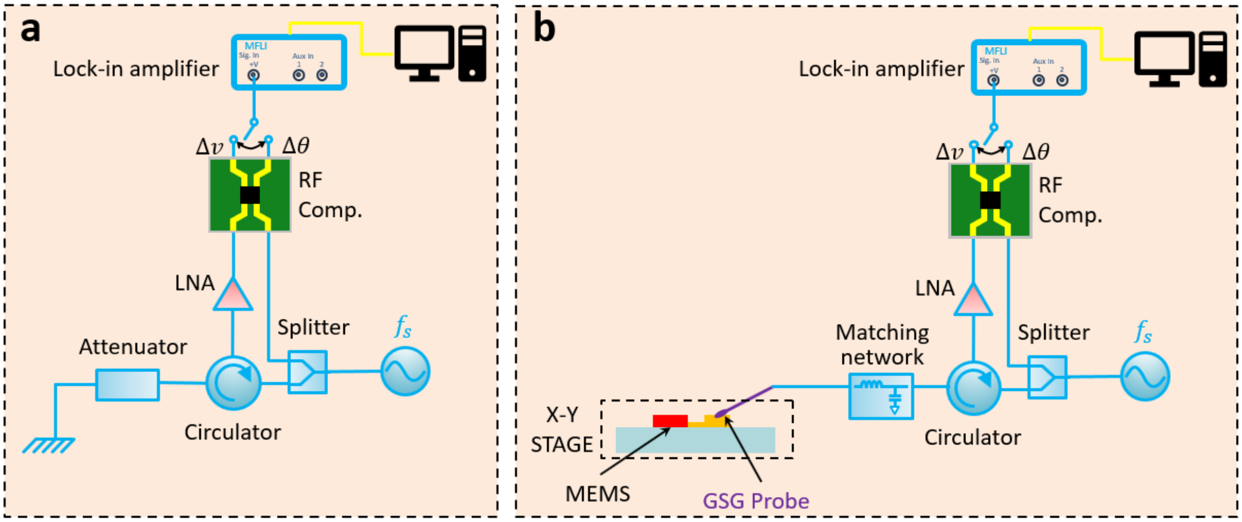

Fig. S8. Experiment setup to characterize the voltage noise spectral density on reflectometric RF MEMS-based IR detectors. (a) Set-up employed to determine the voltage noise spectral density provided the RF phase comparator, $v_{n,PC}(\omega)$. (b) Set-up employed to determine the baseline voltage noise spectral density $v_{n,sys}(\omega)$ in the platform.

2.4.1 Baseline voltage noise spectral density

To experimentally determine the voltage noise spectral density of the phase comparator, $v_{n,PC}(\omega)$, we replaced the RF MEMS device with a manually controlled passive step attenuator (RF-Lambda RKT2G2K10) terminated in a short circuit. Fig. S8a illustrates the schematic of this set-up. In this configuration, the passive attenuator mimics the response of the RF MEMS sensor in terms of attenuation without adding any flicker noise. The attenuation level is adjusted to model the different matching conditions of the platform. Results (Fig 4a in the main paper) confirm that the voltage noise spectral density provided by the phase comparator is in good agreement with the chip specifications [6].

Next, the voltage noise spectral density of the platform $v_{n,sys}(\omega)$ –including the RF MEMS and the matching network– is determined using the set-up shown in Fig S8b. Experimental results shown in Fig. 4a (main paper) are in good agreement with theoretical results from Eq. (S37).

2.4.2 Noise equivalent power

The NEP of the platform can be defined as

$$\mathrm{NEP_{Plat}}(\omega,\lambda) = \frac{v_{n,sys}(\omega)}{R_v(\omega,\lambda)\cdot c_1} \quad [\mathrm{W}/\sqrt{\mathrm{Hz}}]. \tag{S38}$$

This equation relates voltage noise spectral density and responsivity for a given modulation frequency of the IR beam, and corresponds to the platform output measured using a lock-in scheme. Let us consider a device with a high phase-slope quality factor Q_{DL} that is operated at its resonance frequency. In this

scenario, flicker noise will dominate over the phase comparator noise at low and moderate modulation frequencies [i.e., $H^2\phi_{flicker}^2(\omega) \gg v_{n,PC}^2(\omega)$]. There, the NEP can be simplified to

$$\mathrm{NEP_{Plat}}(\omega,\lambda) \approx \frac{H^2\phi_{flicker}^2(\omega)}{2\cdot H\cdot\eta(\lambda)\cdot\chi(\omega)\cdot TCF\cdot R_{th}\cdot Q_{DL}\cdot c_1} = \sqrt{\frac{2\pi b_0}{\omega}}\frac{(R+Z_0)^2}{V_0\cdot 2RZ_0Q_{DU}}\frac{1}{2\eta(\lambda)\cdot\chi(\omega)\cdot TCF\cdot R_{th}\cdot c_1}\quad [W/\sqrt{Hz}], \tag{S39}$$

which in the limit of a perfectly matched resonator (i.e., $R \to R_0$) yields to

$$\mathrm{NEP_{Plat}}(\omega,\lambda) \approx \sqrt{\frac{2\pi b_0}{\omega}}\frac{1}{V_0Q_{DU}}\frac{1}{\eta(\lambda)\cdot\chi(\omega)\cdot TCF\cdot R_{th}\cdot c_1}\quad [W/\sqrt{Hz}]. \tag{S40}$$

As the modulation frequency of the incoming IR beam increases, the noise from the phase comparators becomes more relevant. For sufficiently large frequencies, $v_{n,PC}^2(\omega) \gg H^2\phi_{flicker}^2(\omega)$, and the system NEP yields

$$\mathrm{NEP_{Plat}}(\omega,\lambda) \approx \frac{v_{n,PC}(\omega)}{2\cdot H\cdot\eta(\lambda)\cdot\chi(\omega)\cdot TCF\cdot R_{th}\cdot Q_{DL}\cdot c_1}\quad [W/\sqrt{Hz}]. \tag{S41}$$

2.4.3 Noise equivalent power: predictions

Eq. (S38) states that the NEP of reflectometric MEMS-based IR sensors can been further decreased by (i) decreasing the overall voltage noise spectral density of the system, $v_{n,sys}(\omega)$; and (ii) increasing the overall sensors responsivity, thus maximizing $R_v(\omega,\lambda)$. In the following, we elaborate on the potential performance of the proposed platform to further the NEP and achieve quasi-ideal responses.

First, a $\mathrm{NEP} \approx 450\ \mathrm{pW}/\sqrt{\mathrm{Hz}}$ has been predicted in Fig. 3b and Fig. 4b of the main paper. This response is limited in practice by the 1/f flicker noise arising from the MEMS. This noise component might be removed by developing arrays of MEMS within the same release area and using one device as a reference and another as a sensor. In such differential configuration, the 1/f noise may become a common-mode noise that can be eliminated by the phase detector. There, the noise from the phase comparator, $v_{n,PC}(\omega) \approx 250\ \mathrm{nV}/\sqrt{\mathrm{Hz}}$ (for frequencies > 100 Hz), becomes dominant and a $\mathrm{NEP} \approx 51.3\ \mathrm{pW}/\sqrt{\mathrm{Hz}}$ is predicted for IR beams with a modulation frequency of 100 Hz. This result is close to the ideal response that can be attainable by the device and highlights the significant importance of flicker noise in this technology.

Second, future evolutions of the platform can incorporate dedicated RF phase-comparators instead of a generic off-the-shelf component. As widely described in the literature [18-25], dedicated technology operating at the targeted RF frequency (~ 200 MHz) can yield voltage noise spectral density as low as $v_{n,PC}(\omega) \approx 10\ \mathrm{nV}/\sqrt{\mathrm{Hz}}$ while keeping similar and even higher gain performance. In such scenario, the

thermal noise from the device will become dominant leading to a $\mathrm{NEP_{min}} \approx \mathrm{NEP_{thermal}} = \sqrt{4\mathrm{k_B T^2 R_{th}^{-1}}} = 12\ \mathrm{pW}/\sqrt{\mathrm{Hz}}$ for all frequency offsets.

And third, the sensor IR responsivity can be further increased by boosting the quality factor of the network. Values over 100,000 at possible and realistic in this frequency range using fine-tuned lumped components [26], leading to responsivity values exceeding 65,000 V/W. In that scenario, the predicted NEP at 325 Hz frequency offset would be ~ 125 $\mathrm{pW}/\sqrt{\mathrm{Hz}}$. This number will greatly decrease by removing the flicker noise through a differential MEMS approach as discussed above, leading to a NEP limited by the thermal noise of the detector.

Moving beyond, devices with thermal resistance $\mathrm{R_{th}} > 10^7$ K/W can be achieved using advanced device anchor engineering [1, 27, 28]. Integrating this device into the proposed reflectometric sensing platform would lead to NEP < $1\ \mathrm{pW}/\sqrt{\mathrm{Hz}}$ IR sensors coupled with IR responsivity beyond $100\ \mathrm{kV/W}$. Taken together, these metrics would lead to IR detectors operating at room temperature in the background limited performance (BLIP) regime in which the detector responsivity to background ambient fluctuations exceeds the intrinsic detector noise.

2.4.4 Influence of the RF tone phase noise

The proposed reflectometric platform does not depend on the phase noise of the RF source that excites the device. This important feature relies on the common noise suppression approach integrated within the RF phase comparator and enables the use of relatively noisy RF signals in the platform.

To demonstrate that this is indeed the case, the platform described in the main text is explored using an RF signal generated by the portable RF synthesizer Windfreak SynthUSB3 [29]. Fig. S9a plots the phase noise of the generated signal together with the one reflected from the device, following the scheme illustrated in Fig. 1a of the main paper. The measured phase noise response is well above the one obtained when the low phase-noise Keysight E8663D source is employed (see Fig. 3 in the main paper). The phase noise is high enough to mask the flicker noise generated by the MEMS. Fig. S9b presents the platform NEP for different quality factors of the device calculated using Eq. (S36). Results reveal that lowest NEP values hover around 760 $\mathrm{pW}/\sqrt{\mathrm{Hz}}$, which are higher than those obtained using a low phase-noise RF source.

Fig. S9c plots the voltage noise spectral density measured on the differential system, i.e., after the RF phase comparator that includes the common-noise rejection. Results are plotted versus offset frequency using two different RF sources, namely (a) the RF synthesizer Windfreak SynthUSB3 [29]; and (b) the low

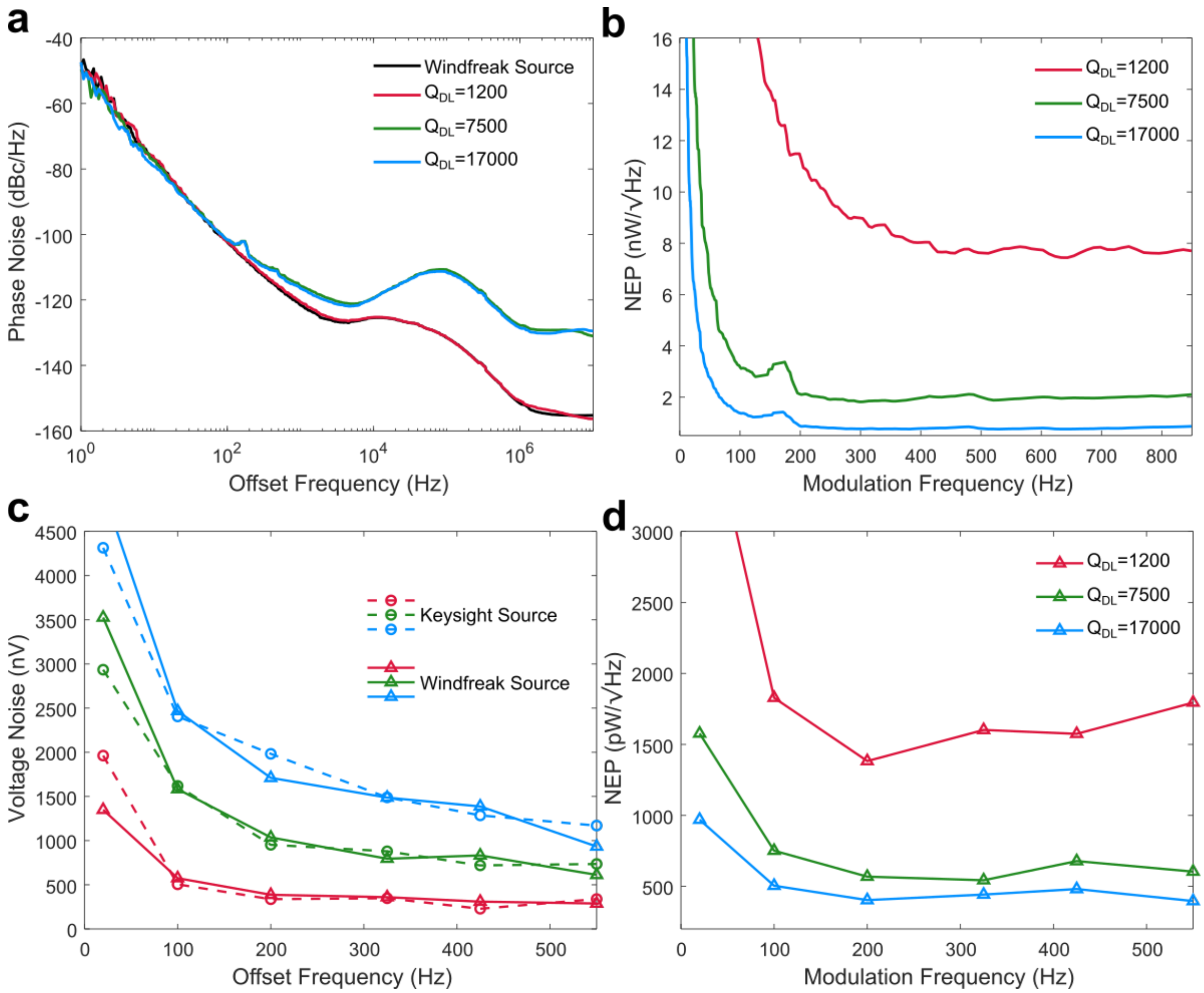

Fig. S9. Noise and NEP of reflectometric RF MEMS-based IR detectors excited with a relatively noisy RF signal. a. Phase noise of the reference RF tone and the RF tone reflected from the IR detector. The initial RF tone is generated by a Windfreak SynthUSB3 source [29]. Results show that both the reference and sensing path exhibit a similar phase noise response. **b.** Noise equivalent power versus the detector phase-slope electrical quality factor Q_{DL}. Results are calculated using Eq. (S36) considering only the signal reflected from the MEMS. **c.** Voltage spectral noise versus offset frequency measured at the output of the RF phase comparator (solid lines). Dashed lines show the voltage noise spectral when the system is excited by a low phase-noise RF source (Fig. 4a main paper). **d.** Noise equivalent power versus the detector phase-slope electrical quality factor Q_{DL}. Results are associated with the complete differential reflectometric platform and are calculated using Eq. (S38) considering measured noise data from panel c and measured responsivity values from Fig. 2 (main paper).

phase-noise Keysight E8663D employed in the main paper. Measured data reveal that the voltage noise spectral density does not significantly depend on the RF source employed, confirming that the intrinsic phase noise of the RF source has been mostly eliminated by the phase common-mode noise rejection module included in the RF phase comparator. Fig. S9d presents the NEP attainable by the platform when excited by the RF synthesizer Windfreak SynthUSB3 [29], which is in good agreement with the values shown in Fig. 4b of the main paper.

3. Numerical simulations

3.1 Multi-physics numerical simulations

The response of RF MEMS-based IR devices was simulated and verified using COMSOL Multiphysics (Fig. S10). Three physics modules are considered in the model: electrical and mechanical physics to model the piezoelectric behavior of the MEMS, and heat transfer to model the heating induced by absorbed IR power which in turn affects the electromechanical properties of the MEMS.

The simulation process begins with a heat transfer simulation, where the absorbed IR light is modeled as a heat source in the SiO_2 layer of the device stack. A temperature change is induced in the MEMS, which results in thermal expansion and a change in the material stiffness. The temperature change plotted against the heat source power gives the thermal resistance of the MEMS (Fig. S10b). Temperature dependent changes in stiffness for each material in the device stack are also considered. Next, a piezoelectric simulation is performed to obtain the electrical network parameters of the device versus

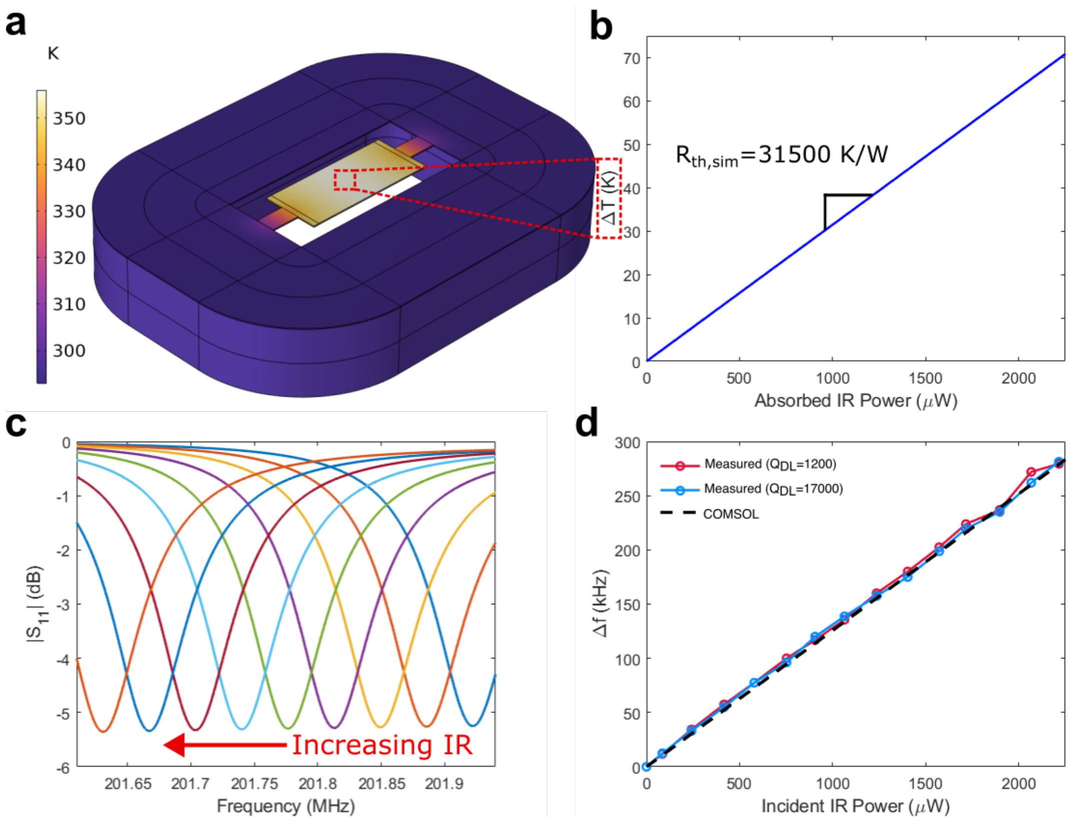

Fig. S10. COMSOL Multiphysics simulation of RF MEMS-based IR sensors. (a) COMSOL model of the RF MEMS IR sensors resonator after it is heated by 2000 µW of absorbed IR power. (b) Temperature change at the center of the MEMS resonator as a function of absorbed IR power. The slope of the curve represents the device's thermal resistance, $R_{th,sim} = 31{,}500\ K/W$, which agrees well with the thermal resistance found experimentally, $R_{th,exp} = 31{,}400\ K/W$. (c) Simulated reflection coefficient of the devices versus incoming IR power. (d) Plot of the simulated (black dashed line) and measured (solid lines) frequency shift. Experimental results are obtained for different values of the device quality factor.

Table S1. Material parameters employed in COMSOL Multiphysics to simulate the response of RF MEMS-based IR sensors. These parameters have been obtained from Refs. [30-33] and were tuned < 1% to match experimental results.

	AlN	Pt	SiO_2	Si
ρ (kgm^{-3})	3300	21450	2200	2329
κ ($Wm^{-1}K^{-1}$)	42	71.6	1.4	130
Y_o (GPa)	-	168	70	-
T_Y (10^{-4}/K)	-	-3.38	+1.7	-
$C_{11,o}$ (GPa)	410			
$C_{12,p}$ (GPa)	149			
$C_{13,o}$ (GPa)	99			
$C_{33,o}$ (GPa)	389			
$C_{44,o}$ (GPa)	125			
$C_{66,o}$ (GPa)	130			
T_{c11} (10^{-6}/K)	-37			
T_{c12} (10^{-6}/K)	-1.8			
T_{c13} (10^{-6}/K)	-1.8			
T_{c33} (10^{-6}/K)	-65			
T_{c44} (10^{-6}/K)	-50			
T_{c66} (10^{-6}/K)	-57			

frequency. This simulation process is repeated for multiple IR powers to attain a resonance frequency shift versus incident IR power plot (Fig. S10c), which agrees very well with experimental results (Fig. S10d). In the software, the heat source represents the absorbed IR power, not incident, therefore in post-processing the absorbed IR power was divided by the metasurface absorption at $\lambda_0 = 5.94\ \mu\text{m}$. Material properties have been taken from literature [30-33] and are tabulated in Table 1 for convenience. Temperature dependent elasticity was modeled using $C_{ij}(T) = C_{ij,o}\left(1 + T_{cij}(T - T_o)\right)$, where $C_{ij,o}$ is the room temperature stiffness coefficient, T_{cij} is the temperature coefficient of elasticity, and $\Delta T = T - T_o$ is the temperature change from room temperature [30]. For Pt and SiO_2, the temperature dependence of Young's modulus is modeled using a similar expression, $Y(T) = \left(Y_o + T_Y(T - T_o)\right)$ [31].

3.2 Circuit simulations

The response of the proposed reflectometric IR detectors has been simulated and verified using Keysight Advanced Design System (ADS) [34]. The MEMS electrical response was fit to an MBVD circuit model (see Fig. S2), while the additional RF system's components employed on the platform were modeled with data provided by their respective vendors. The resulting circuit model of the reflectometric platform is shown in Fig. S11. The inputs of the RF phase comparator are modeled by two 50-Ω loads, representing the input impedances of the two RF input ports. The difference in phase between the two RF signals at the loads is multiplied by the RF comparator transfer function to obtain the DC output voltage that is proportional to the incoming IR light.

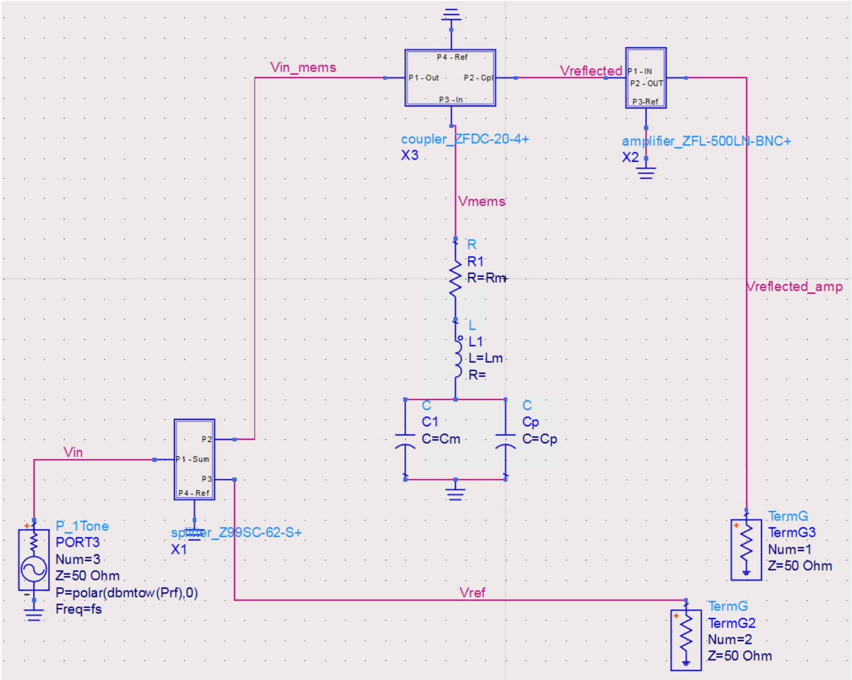

Fig. S11. Circuit simulation of reflectometric RF MEMS-based IR detectors using Keysight ADS. The RF MEMS is represented by the MBVD model shown in Fig. S2. Experimental benchtop components are represented by block components which contain vendor provided S-parameter matrices. The matching network is modeled with an LC network. The two inputs to the RF comparator are represented by two 50-Ω loads.

As theoretically described above in Eq. (S8) and Eq. (S11), the resonance shift of the sensor upon IR illumination can be modeled using an MBVD model of the RF MEMS sensor in which a variable capacitor has been added. This variable capacitor can be obtained as

$$C_{\Delta T}(\omega,\lambda) = C\big(1-2\cdot TCF\cdot \Delta T(\omega,\lambda)\big) = C\big(1-2\cdot TCF\cdot \eta(\lambda)\cdot\chi(\omega)\cdot P_{IR}(\lambda)\cdot R_{th}\big) \qquad (S42)$$

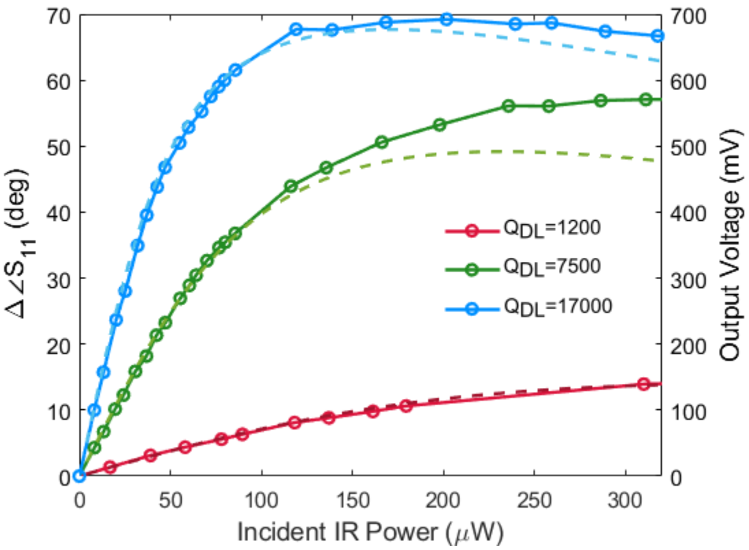

Fig. S12. Performance of reflectometric RF MEMS-based IR sensors: circuit simulations versus experimental data. Change in phase of the RF signal reflected on the device versus incident IR power obtained through circuit simulations (dashed lines) and experiments (solid lines). Results confirm that circuit simulation accurately predicts both the linear and nonlinear sensor response. Parameters are identical to those given in Fig. 2a of the main paper.

where all parameters involved in the equations were previously defined. Using the frequency shift responsivity determined from COMSOL Multiphysics simulations in Fig. S10, the additional capacitance added by IR light absorbed by the device can be characterized using circuit simulations. The output voltage as a function of IR power is plotted in Fig. S12 (dotted line) and compared to measured results. Simulations are repeated for various Q_{DL} values of the network, demonstrating a very good matching between simulation and experiment results for all cases considered. This study further validates the accuracy of experimental results and demonstrates that the sensor performance is well captured using numerical and circuit simulations and that these tools can be further employed to further optimize the platform response.

4. Experimental characterization

4.1 Experimental set-up: Overview

Figure S13 illustrates the experimental set-up employed to characterize reflectometric RF MEMS-based IR sensors. It is composed of a precision motorized XY translation stage coupled to a vacuum chuck that holds the chip under test [16]. A standard 150-µm GSG 40A micro-probe [35] (GGB, 40A-GSG-150-LP) is used to make electrical connection with the device. Two different approaches are employed to characterize the IR response of the sensors.

The first method is the standard technique described in Refs [9, 14-16] and consists of connecting the RF-MEMS with a vector network analyzer (VNA, Copper Mountain Technologies TR1300/1). Using automatized software [16], it is possible to monitor the scattering parameters of the device under test versus the incoming IR beam to determine the sensor responsivity in terms of frequency shift – as described in Eq. (S8) in this document. Measured admittance parameters of the device under test are shown in Fig. S2. This approach is employed to measure the frequency shift of the device's resonance frequency plotted in Fig. S10d. Note that the frequency shift only depends on TCF and the temperature change within the device, as modelled in Eq. (S8), and therefore it is independent of the matching network. For the same reason, no saturation is observed for the frequency shift within the optical power range considered in our experiments.

The second method is the reflectometry approach proposed in this paper. To this purpose, a low phase-noise RF tone at f_s generated by a Keysight E8663D source is split into two RF branches using an RF splitter (MiniCircuit Z99SC-62-S+): one is fed into the RF comparator and used as a reference signal, and the other is sent to the IR detectors, reflected, and directed to the RF comparator using a circulator (RF Lambda RFLC090M19M23). A low-noise amplifier (RF Lambda R18M66MSA) is employed to compensate for the amplitude loss suffered by the RF tone upon reflection on the IR sensor. The shielded RF comparator

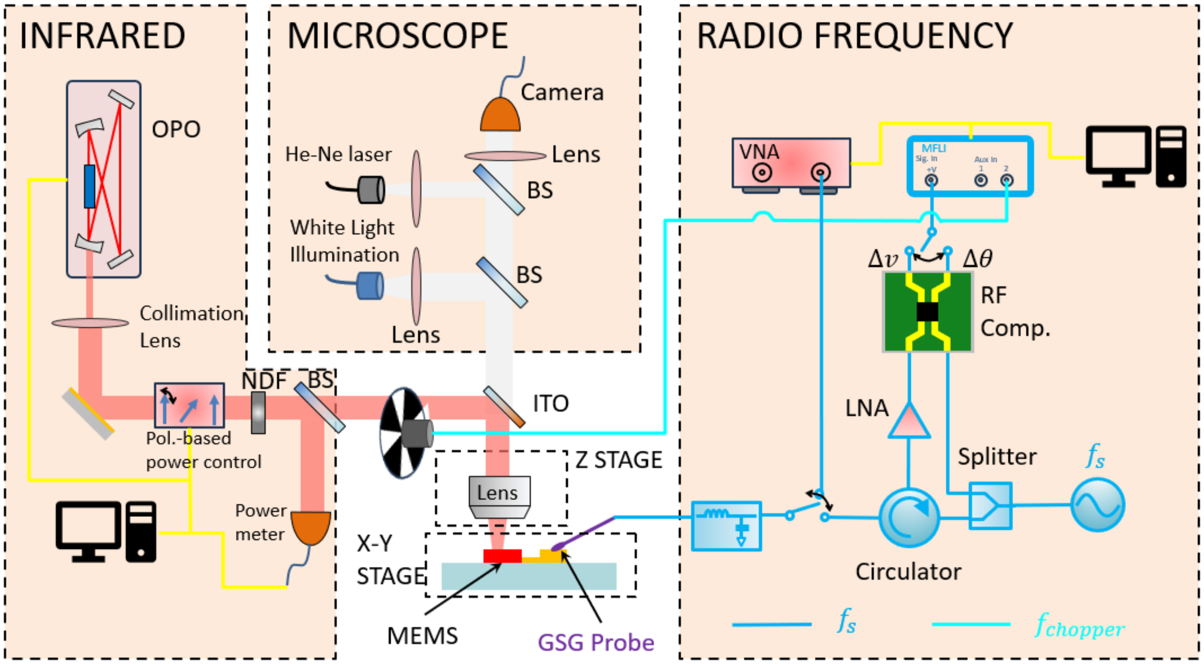

Fig. S13. Experimental setup to characterize reflectometric RF MEMS-based IR detectors. OPO: optical parametric oscillator; NDF: neutral density filters; BS: beam splitter; ITO: indium tin oxide splitter; LNA: low noise amplifier; VNA: vector network analyzer; RF comp.: radio frequency comparator. Infrared part: an IR beam is generated by the optical parametric oscillator and collimated by a ZnSe lens with a focal length of 1000 mm. The power of the IR beam is first attenuated using neutral density filters and then manipulated via a polarization-based power control unit which consists of two polarizers, one polarization rotator, and an optical power meter. The beam power is fine-tuned by controlling the mechanical rotator. The IR beam is chopped, guided by low-loss gold mirrors, and partially focused on the MEMS device with a CaF_2 lens (focal length: 40 mm). The MEMS is placed off the focal plane of the lens for improved uniformity in coverage as opposed to the focal plane. Microscope imaging system: a visible-light camera and a white-light lamp are employed to assist in the RF probing and pinpointing the MEMS. An essential component to combine both visible and infrared light into the same beam path is an ITO splitter which reflects the IR light and transmits the visible light. Radiofrequency part: a low-noise RF signal at f_s frequency is generated by a signal source and split equally by an RF splitter. One signal is directed to the matching network and MEMS resonator via a circulator. After it gets reflected by the device, the RF signal is coupled to the RF comparator through the circulator. The other RF signal coming from the splitter serves as a reference and is also fed to the RF comparator. The output voltage generated by RF comparator, which is linearly proportional to the relative phase difference between the two RF signals, is fed to a lock-in amplifier. A vector network analyzer can also be used to measure the device frequency response of the resonator. All equipment is controlled and automated by a computer program.

(Valon Model 4002 based on the Analog Devices AD8302 chip, see Fig. S7) provides an output voltage that linearly depends on the phase difference between the two RF tones that feed it. Using automatized software, the RF comparator output voltage is monitored versus the incoming IR beam to determine the sensor responsivity in V/W.

In both approaches, a coherent IR beam is generated by an optical parametric oscillator (OPO, EKSPLA, PT277-XIR). This OPO provides high output power, ultra-wide wavelength tunability, and enables selection of the desired output wavelength through a computer program. A broadband ZnSe lens (Thorlabs LA5370) with a focal length of 1 m is used to collimate the IR beam. The optical power level of the IR beam is then adjusted using the power control system described below. After that, an optical chopper is added to the

IR path to modulate the intensity of the IR beam. Next, an indium tin oxide (ITO) plate (SPI 06411-AF) is employed to reflect the IR beam toward the stage. ITO is employed here because it allows bringing visible light from a different source into the IR optical path. After reflecting from the ITO plate, the IR beam is focused by a CaF_2 lens (Thorlabs LA5370) onto the stage. Additionally, as described below, an imaging system consisting of a visible-light camera, a He-Ne laser, a white-light source, and a set of lenses has been built to accurately align the device under test.

4.2 IR beam: power control

The coherent IR beam generated by the OPO has an optical power that varies significantly versus the operation wavelength. To overcome this challenge, an optical power control system has been implemented. The system relies on an IR broadband polarizer (Thorlabs WP25H-B) mounted on a mechanically controlled rotation stage, a second polarizer that ensures that all beams emerging from the system have similar polarization, neutral density filters, and an optical power meter (Thorlabs, S401C). First, broadband neutral density filters (Thorlabs NDI03A) are added to the IR path to roughly obtain a desired power range. Then, the optical power is fine-tuned by adjusting the angle θ between the polarizers using the rotation stage. The output power after the system can be modelled by

$$\mathrm{P_{out}} = \mathrm{P_{in}} \cdot \mathrm{A} \cdot \cos^2(\theta), \tag{S43}$$

where A characterizes the attenuation provided by the neutral density filters. The IR beam is then split into two using a CaF_2 IR beamsplitter (Thorlabs BSW510): one beam is directed toward the device under test, and the other is directed to the optical power meter. A computer program implementing a feedback control loop is employed to (i) monitor the received optical power; and (ii) modify the rotation angle θ by mechanically rotating the polarizer to obtain targeted optical power levels. Simultaneously, the IR power received on the stage at the DUT position is monitored with an MCT IR photodetector (Thorlabs PDAVJ10). This allows us to accurately correlate the IR power impinging on the DUT versus the angle θ of the IR polarizer and the neutral density filters. By adjusting θ using a computer program, this experimental set-up permits us to perform IR power-dependent measurements at desired IR wavelengths.

Fig. S10d further confirms the accuracy of the developed power-dependent system by using full-wave numerical simulations in COMSOL Multiphysics.

4.3 IR beam: spatial profile

To determine the sensor responsivity and the minimum IR power that can be detected by the proposed platform, it is critical to accurately resolve the IR power impinging on the detector. The detector area, 140 µm x 60 µm (see Fig. 1b in the main paper), is significantly smaller than the beam width of the

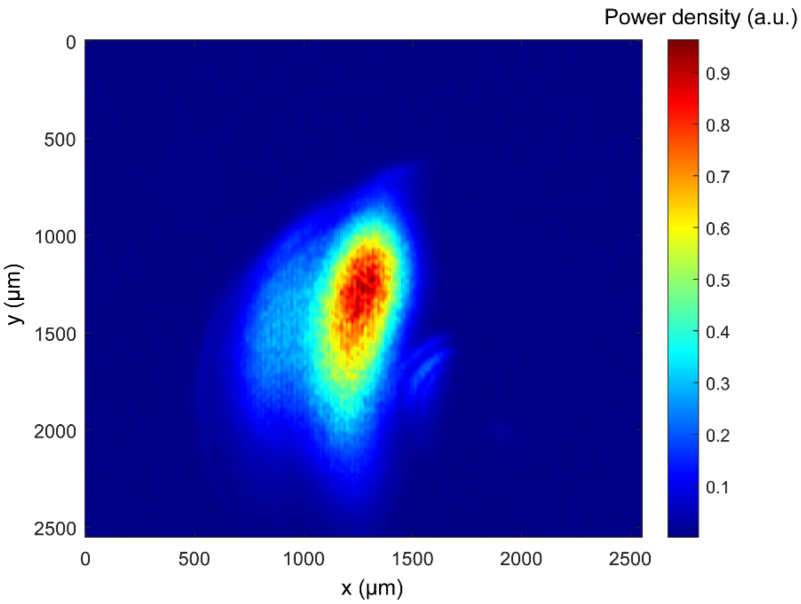

Fig. S14. Normalized spatial power density of the IR beam impinging on the device under test.

incoming IR beam. The spatial power density of the IR beam is determined as follows. First, the beam profile is obtained with an IR camera to resolve its normalized power density. Second, the total power of the IR beam is measured using a MCT IR photodetector (Thorlabs PDAVJ10). And third, the power density of the IR beam is obtained by integrating the normalized power density over the measurement area and relating it to the measured power.

A mid-IR/far-IR beam profiler (DataRay, S-WCD-IR-BB-30) [36] is employed to measure the profile of the incoming IR beam (see Fig. S14). The pixel size of the beam profiler is ~17 µm. One important step in this characterization is to precisely locate the beam profiler at a vertical position equivalent to the top surface of the MEMS resonator so that the power density at this specific height can be accurately measured. To do so, a stabilized He-Ne laser (Thorlabs HRS015B) is employed to assist in the alignment [37]. The red laser beam generated by the He-Ne is brought to the IR beam path through the ITO layer, which is quasi-transparent at visible frequencies [38]. When the visible beam from the He-Ne passes through the focusing lens, it attenuates and focuses on a different focal depth than the IR light. The red-light focal point is employed as reference position of the top surface of the IR camera. Such position is identified using a visible camera and then employed to assist in probing and pinpointing the MEMS resonator. The focal point of the red light is adjusted by changing the diverging angle of the red-light beam through various optical lenses. The different focal depths of the IR and visible light was exploited to achieve a fully focused visible beam together with a defocused IR beam. Such a response is desired in our set-up to obtain a uniform IR power density over the detector sensing area.

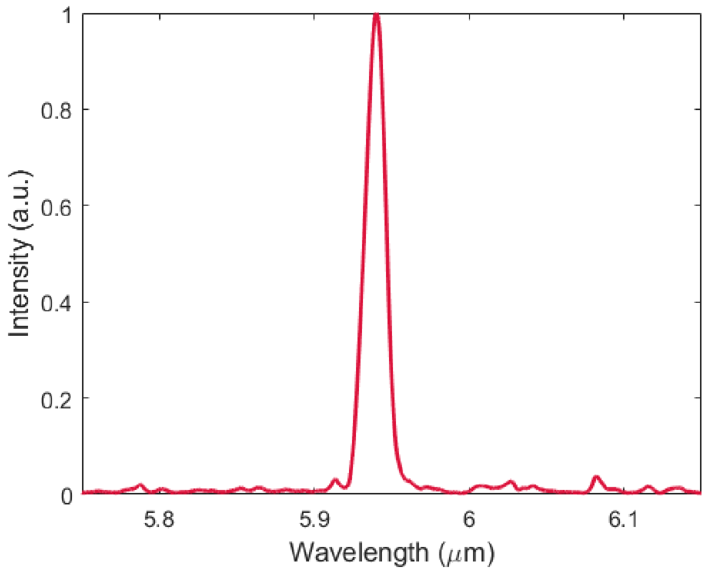

Fig. S15. Linewidth measurement of the IR beam generated by the OPO EKSPLA PT277XIR. The OPO is set to generate an IR beam oscillating at 5.94 μm.

The vertical position of the MEMS device is then determined by focusing the He-Ne laser beam on the top of the metasurface such that the sensor plane of the beam profiler will be the same height as the MEMS.

4.4 IR beam: spectral bandwidth

The optical spectrum of the IR beam generated by the OPO (EKSPLA, PT277XIR) has been characterized. To this purpose, the beam is routed into the inlet port of an FTIR tool (Bruker INVENIO coupled to a Hyperion 2000 microscope). The FTIR is configured to admit external sources as input IR beams. The characterization has been performed in reflection mode, using a gold mirror (Thorlabs, PF10-03-M02) as a reference. The linewidth of the OPO is measured using the FTIR and is found to be 15.4 nm (4.57 cm^{-1}) and constant across the wavelength range considered in this work (~ 5 to 7 µm). Fig. S15 shows an example of the measured spectrum, which agrees well with the OPO specifications [39].

4.5 Influence of power and LNA in the phase noise of RF signals

Fig. S16 shows the phase noise of the RF tone generated by the Keysight E8663D source for different power levels. Measurements are first carried out by connecting the generator directly to the signal source analyzer without including the RF low-noise power amplifier. The center frequency is set to 201.92 MHz. Output powers ranging from -8 dBm to -2 dBm show no significant phase noise variations. Additionally, the phase noise of the RF signal is measured when the low noise amplifier (RF Lambda R18M66MSA) is included in the RF path, confirming that its influence on the phase noise of the RF tone is not significant.

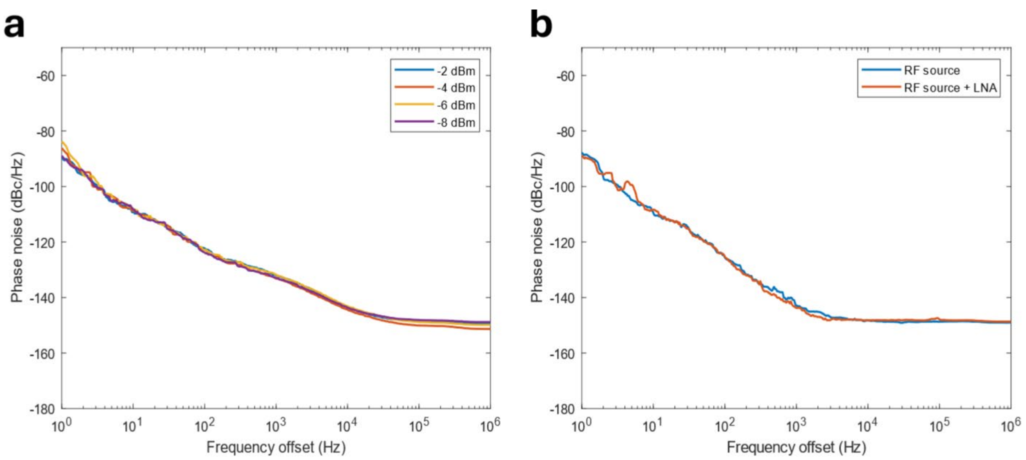

Fig. S16. Noise characterization of the RF source and low noise amplifier. a. Phase noise of the RF signal generated by the Keysight E8663D source for different output powers at 201. 92 MHz. The phase noise at different output power is very similar, which confirms that the power of the RF signal does not significantly affect the noise of the signal. **b.** Phase noise of the RF signal with and without including a low noise amplifier. The comparable noise levels confirm that the amplifiar does not add significant noise to the RF signal.

4.6 Power-independent absorption profile

We highlight that the absorption profile of the RF MEMS detector does not change for moderate/low IR power. This is because the absorption profile of the device is defined by the electromagnetic properties of the materials that compose the metasurface (SiO2, Au). Entering the nonlinear regime of these materials would require high-power beams that cannot be easily obtained in practice, with peaks well over kW/cm levels [40]. Instead, moderate-power IR beams can bring the device into the nonlinear regime in the sense that variations of the output do not linearly follow the incoming power.

More specifically, we demonstrated spectral selectivity in two different manners. First, we measured IR responsivity at different wavelengths using a widely tunable IR laser. Second, we measured the absorption profile of the device using a FTIR. As demonstrated in Fig. 2b of the main paper, there is a remarkable agreement in the profile of these measurements. Additionally, Fig. 2a (main paper) shows power-dependent IR measurements that require power control only at 5.94 um. Results show that the device behaves in the expected linear regime for low and moderate IR power. For larger IR powers, the device enters a nonlinear regime in which the phase variations of the reflected RF signal do not vary linearly with the power of the incoming IR beam. Note that the maximum IR power employed in our study is quite moderate, with values up to 300 μW, but still larger than the power required to go above the damage threshold of conventional semiconductor heterostructures.

5. References

[1] Y. Hou, M. Zhang, G. Han, C. Si, Y. Zhao, and J. Ning, "A review: Aluminum nitride MEMS contour-mode resonator," *Journal of Semiconductors,* vol. 37, p. 101001, 2016.

[2] M. Steer, "Microwave and RF design," *NC State University: Raleigh, NC, USA,* 2019.

[3] H. Bhugra and G. Piazza, *Piezoelectric MEMS resonators*: Springer, 2017.

[4] H.-T. Chen, A. J. Taylor, and N. Yu, "A review of metasurfaces: physics and applications," *Reports on progress in physics,* vol. 79, p. 076401, 2016.

[5] L. Valon Technology. (2018). *4002 Phase-Gain Detector Module* Available: https://www.valonrf.com/uploads/1/1/7/3/117370920/4002_datasheet.pdf

[6] A. Devices. *LF–2.7 GHz RF/IF Gain and Phase Detector*. Available: https://www.analog.com/media/en/technical-documentation/data-sheets/ad8302.pdf

[7] P. L. Richards, "Bolometers for infrared and millimeter waves," *Journal of Applied Physics,* vol. 76, pp. 1-24, 1994.

[8] J. Segovia-Fernandez and G. Piazza, "Thermal nonlinearities in contour mode AlN resonators," *Journal of microelectromechanical systems,* vol. 22, pp. 976-985, 2013.

[9] Y. Hui, J. S. Gomez-Diaz, Z. Qian, A. Alu, and M. Rinaldi, "Plasmonic piezoelectric nanomechanical resonator for spectrally selective infrared sensing," *Nature communications,* vol. 7, p. 11249, 2016.

[10] U. L. Rohde, E. Rubiola, and J. C. Whitaker, *Microwave and wireless synthesizers: theory and design*: John Wiley & Sons, 2021.

[11] H. J. Kim, S. I. Jung, J. Segovia-Fernandez, and G. Piazza, "The impact of electrode materials on 1/f noise in piezoelectric AlN contour mode resonators," *AIP Advances,* vol. 8, 2018.

[12] P. W. Kruse, *Uncooled thermal imaging: arrays, systems, and applications* vol. 51: SPIE press, 2001.

[13] A. Cleland and M. Roukes, "Noise processes in nanomechanical resonators," *Journal of applied physics,* vol. 92, pp. 2758-2769, 2002.

[14] Y. Hui, S. Kang, Z. Qian, and M. Rinaldi, "Uncooled infrared detector based on an aluminum nitride piezoelectric fishnet metasurface," *Journal of Microelectromechanical Systems,* vol. 30, pp. 165-172, 2021.

[15] M. E. Gülseren, M. Benson, R. W. Parker, J. Segovia-Fernandez, E. T.-T. Yen, and J. S. Gómez-Díaz, "Experimental Study of Spectrally-Selective MEMS/metasurface Infrared Detectors," *IEEE Sensors Journal,* 2024.

[16] M. Benson, R. W. Parker, M. E. Gülseren, and J. S. Gómez-Díaz, "Automatic Characterization of High-Performance MEMS-Based IR Sensors," *IEEE Access,* 2024.

[17] Y. Fang, A. Armin, P. Meredith, and J. Huang, "Accurate characterization of next-generation thin-film photodetectors," *Nature Photonics,* vol. 13, pp. 1-4, 2019.

[18] J.-C. Chien, P. Upadhyaya, H. Jung, S. Chen, W. Fang, A. M. Niknejad*, et al.*, "2.8 A pulse-position-modulation phase-noise-reduction technique for a 2-to-16GHz injection-locked ring oscillator in 20nm CMOS," in *2014 IEEE International Solid-State Circuits Conference Digest of Technical Papers (ISSCC)*, 2014, pp. 52-53.

[19] J.-C. Chien and A. M. Niknejad, "Oscillator-based reactance sensors with injection locking for high-throughput flow cytometry using microwave dielectric spectroscopy," *IEEE Journal of Solid-State Circuits,* vol. 51, pp. 457-472, 2015.

[20] H. Wang, C.-C. Weng, and A. Hajimiri, "Phase noise and fundamental sensitivity of oscillator-based reactance sensors," *IEEE Transactions on Microwave Theory and Techniques,* vol. 61, pp. 2215-2229, 2013.

[21] J.-C. Chien and A. M. Niknejad, "Design and analysis of chopper stabilized injection-locked oscillator sensors employing near-field modulation," *IEEE Journal of Solid-State Circuits,* vol. 51, pp. 1851-1865, 2016.

[22] J.-C. Chien, "A 1.8-GHz near-field dielectric plethysmography heart-rate sensor with time-based edge sampling," *IEEE Journal of Solid-State Circuits,* vol. 55, pp. 615-628, 2019.

[23] M. Elkholy and K. Entesari, "A Wideband Low-Power LC-DCO-Based Complex Dielectric Spectroscopy System in 0.18-$\mu\text {m} $ CMOS," *IEEE Transactions on Microwave Theory and Techniques,* vol. 65, pp. 4461-4474, 2017.

[24] S. Hao, T. Hu, and Q. J. Gu, "Time-Amplifier Enhanced Phase Noise Filter," *IEEE Microwave and Wireless Components Letters,* vol. 28, pp. 699-701, 2018.

[25] T. Hu, S. Hao, and Q. J. Gu, "A bang-bang PD based phase noise filter with 23 dB noise suppression," in *2017 IEEE MTT-S International Microwave Symposium (IMS)*, 2017, pp. 1137-1140.

[26] B. Razavi and R. Behzad, *RF microelectronics* vol. 2: Prentice hall New York, 2012.

[27] M. Fujiyoshi, T. Ozaki, Y. Omura, H. Funabashi, T. Akashi, and Y. Nonomura, "Improved anchor design for flat MEMS structure by suppressing deformation due to buried-oxide stress on silicon-on-insulator wafer," *Journal of Micromechanics and Microengineering,* vol. 31, p. 045001, 2021.

[28] L. Li, W. He, Z. Tong, H. Liu, and M. Xie, "Q-factor enhancement of coupling bragg and local resonance band gaps in single-phase phononic crystals for TPOS MEMS resonator," *Micromachines,* vol. 13, p. 1217, 2022.

[29] L. Windfreak Technologies. (2025). *SynthUSB3: 12.5MHz – 6.4GHz USB RF Signal Generator*. Available: https://windfreaktech.com/product/synthusb3-6ghz-rf-signal-generator/

[30] R. R. Reeber and K. Wang, "High temperature elastic constant prediction of some group III-nitrides," *Materials Research Society Internet Journal of Nitride Semiconductor Research,* vol. 6, p. e3, 2001.

[31] R. Farraro and R. B. Mclellan, "Temperature dependence of the Young's modulus and shear modulus of pure nickel, platinum, and molybdenum," *Metallurgical Transactions A,* vol. 8, pp. 1563-1565, 1977.

[32] M. Gad-el-Hak, *The MEMS handbook*: CRC press, 2001.

[33] C. Duquenne, M.-P. Besland, P. Tessier, E. Gautron, Y. Scudeller, and D. Averty, "Thermal conductivity of aluminium nitride thin films prepared by reactive magnetron sputtering," *Journal of Physics D: Applied Physics,* vol. 45, p. 015301, 2011.

[34] "Advanced Design System (ADS)," ed: Keysight Technologies.

[35] G. Industries. (2024). *Model 40A*. Available: https://ggb.com/home/model-40a/

[36] D. Inc. (2024). *WinCamD-IR-BB*. Available: https://store.dataray.com/all-products/beam-profiling-cameras/wincamd-ir-bb/?srsltid=AfmBOop26-jLCsA62mOPbv1J3RInzZdYziETY_xRAMpJkE5pIkKdhmq-

[37] T. Inc. (2025). *HRS015B - Stabilized HeNe Laser, 632.992 nm (Vacuum), 1.2 mW, Polarized* Available: https://www.thorlabs.com/thorproduct.cfm?partnumber=HRS015B

[38] R. Bi, C. Zheng, W. W. Yu, W. Zheng, and D. Wang, "Breaking through the plasma wavelength barrier to extend the transparency range of ultrathin indium tin oxide films into the far infrared," *Journal of Applied Physics,* vol. 134, 2023.

[39] Ekspla. (2025). *Tunable wavelength lasers*. Available: https://ekspla.com/products/tunable-wavelength-lasers/

[40] R. Boyd, Nonlinear Optics, Academic Press, 2020.
