# Supplementary material for: High responsivity IR sensing based on reflectometric RF MEMS
Source: Nat Commun. 2026 Jun 16;17:7625. doi: 10.1038/s41467-026-74152-3 (PMC13429729; doi:10.1038/s41467-026-74152-3)
Supplement: Supplementary file 2 — Transparent Peer Review file [file 41467_2026_74152_MOESM2_ESM.pdf]

nature portfolio

Peer Review File

High Responsivity IR Sensing based on Reflectometric RF-MEMS

Corresponding Author: Dr Juan Sebastian Gomez-Diaz

This file contains all reviewer reports in order by version, followed by all author rebuttals in order by version.

Version 0:

Reviewer comments:

Reviewer #1

(Remarks to the Author)
The manuscript presents a MEMS-based infrared (IR) sensing concept that combines a state-of-the-art piezoelectric resonator with LC-matched RF reflectometry and phase-sensitive detection. The claimed novelty lies in achieving high responsivity and in reading out IR-induced thermal frequency shifts through the phase change of a reflected RF tone, combined with an open-loop phase detection scheme, similar to conventional frequency-tracking methods such as lock-in detection. The authors further propose that the sensor's responsivity can be electrically tuned by adjusting the loaded Q-factor of the RF matching network.

Major Comments

- Figures of merit and comparison to state-of-the-art detectors

The authors emphasize responsivity as the key performance metric and even highlight it in the title. I strongly disagree with this notion.
For meaningful comparison with other IR detectors, whether electrical or mechanical, the relevant figures of merit are the noise-equivalent power (NEP) and the time constant. These two parameters together define a detector's sensitivity and speed independently of the specific readout configuration.

Responsivity (in V/W or A/W) is only appropriate for purely electrical detectors where the noise is also electrical (e.g., Johnson noise or 1/f noise). Even in such cases, responsivity alone does not characterize performance, as it ignores the noise level and thus the signal-to-noise ratio. For mechanical detectors, where thermomechanical noise often dominates, the transduction from frequency or displacement to voltage is irrelevant to the detector's intrinsic limits.

Following this logic, the NEP reported here does not surpass that of existing MEMS/NEMS IR detectors. Consequently, the manuscript's impact is limited. When normalized to detector area, the specific detectivity of the presented device (D* ~ 2e7 Jones) is two orders of magnitude below state-of-the-art values (D* > 1e9 Jones), as demonstrated, for example, in Ref. 25, in Nano Letters 2025, DOI: 10.1021/acs.nanolett.5c03415, or in APL Photonics 2024 10.1063/5.0238977, where the latter also features a metamaterial absorber offering a defined spectral absorption.

I find it commendable that the authors follow a well-defined and rigorous procedure to characterize their detector's performance, as outlined in Ref. 5. This is good scientific practice, and future studies in the field would certainly benefit from adopting similarly standardized methods. However, I do not agree with the authors' implication that earlier works lacking such systematic characterization are, therefore, in essence, not trustworthy. The introduction's rather sweeping critique of prior studies is unjustified; many of those reports have demonstrated meaningful advances in detector performance, even if their evaluation protocols were less standardized.

- Novelty of the readout concept

The proposed RF reflectometry scheme essentially constitutes an open-loop implementation with phase detection. The phase of the reflected signal is monitored at a fixed frequency to infer small resonance shifts. This approach is functionally

equivalent to the phase error signal used in lock-in detection, e.g., in a phase-locked loop (PLL), a well-established closed-loop frequency-tracking method based on phase comparison.

Therefore, the “phase comparison” technique presented here does not represent a new sensing or readout principle; it is simply the open-loop analog of conventional PLL-based frequency demodulation. Moreover, such open-loop operation will likely be challenging to implement in practice, as the resonance frequency of the highly temperature-sensitive MEMS resonator will drift with environmental variations.

In addition, RF reflectometry using LC-coupled MEMS or NEMS resonators is not new. Similar approaches have been reported previously for capacitively coupled nanomechanical systems (see Nano Letters 2006, DOI: 10.1021/nl062278g; Nature 2014, DOI: 10.1038/nature13029).

It is also unclear how a loaded Q greater than the intrinsic Q of the resonator could be achieved. When the electrical (RC) and mechanical resonators couple, especially in a piezoelectric device, the resulting system should exhibit a total Q limited by the most lossy component, which here is the mechanical resonator (Q = 2200). A more detailed discussion of this point would be helpful.

Minor Comments

- Spectral absorptance and application context
The manuscript highlights the advantage of a narrow spectral absorptance but does not specify where such a characteristic would be particularly useful. A clear application example would strengthen the argument. For instance, narrowband detection is beneficial for gas sensing, but would be impractical for spectroscopy, which typically requires hundreds or thousands of resonators to achieve broadband spectral coverage for mid-IR fingerprinting of unknown samples.

- References
Several references are incomplete or incorrect:
Page 1: “…using either optical 22–25 or electrical readout 17,19,20.”

The concept in Ref. 22 was later implemented as a NEMS array with electrical readout, achieving NEP = 27 pW/√Hz (Phys. Rev. Appl. 2018, 9, 024016).
Ref. 23 also describes a NEMS detector with electrical readout and NEP = 7 pW/√Hz.

Page 2: “…and has recently attracted attention for probing quantum devices 18,27.”
Ref. 18 does not concern quantum devices; it describes an earlier version of the present detector.

Page 2: “…while optical readouts require bulky interferometric setups incompatible with chip-scale platforms 22,23.”
As noted above, both Refs. 22 and 23 actually employ electrical readout.

These are only a few examples; the authors should carefully check all references for correctness and completeness.

- Page 3, eq. for the frequency shift:
Conventionally, the absorption coefficient is expressed in units [1/m]. The absorption defined here is rather the absorptance, a dimensionless quantity. For clarity, the authors should revise the notation and terminology accordingly.

- Page 3, thermal resistance Rth:
What is the dominant thermal loss mechanism in the system? Radiation, thermal conduction, or convection?

- Page 4, eq.(4):
What is the maximum input RF voltage V0 that can be sent to the MEMS?
Does the RF drive contribute to self-heating, and if so, how does this affect the overall noise performance?

Summary and Recommendation

The manuscript presents a technically sound engineering demonstration but lacks the conceptual novelty and physical insight expected for Nature Communications. The performance improvement over existing piezoelectric IR detectors (Ref. 17) is incremental rather than fundamental. The achieved specific detectivity is two orders of magnitude below the current state of the art.

I therefore do not recommend publication in Nature Communications.
The work would be better suited to a specialized MEMS or sensor journal, where solid incremental advances in device design and readout schemes are valued.

Reviewer #2

(Remarks to the Author)
This manuscript reports a room-temperature MIR detector with spectral selectivity. While RF reflectometry–based MIR detectors have appeared recently, they typically rely on tracking small frequency shifts and have shown limited performance.

In contrast, the present work detects the reflection phase of an RF tone coupled to a MEMS resonator, establishing a new approach that enables high-resolution MIR measurement. The wavelength resolution is evaluated with a laser source, and the data convincingly support the claims and novelty. In my view, this paper is suitable for publication in Nature Communications, pending clarification of the following points:

1. Introduction—spectral selection elements
The Introduction focuses on detector comparisons for implementing an on-chip IR spectroscopy system but omits the spectral-selection components (e.g., gratings/AWGs, metasurfaces, MEMS Fabry–Pérot, interferometric approaches). This makes the argument feel incomplete. Please briefly discuss candidate spectral elements and their trade-offs to strengthen the narrative.

2. Reliance on broadband blackbody sources—what exactly is the issue?
You list reliance on broadband blackbody sources as a challenge, but the problem is stated abstractly. The device itself has spectral selectivity; however, a non-selective evaluation source means the measured signal is the convolution of the device's spectral responsivity with the blackbody spectrum. Please clarify whether your point is that this setup makes it difficult to fully demonstrate the device-intrinsic selectivity.

3. Limits of conventional electrical readout
You suggest that conventional electrical readouts have difficulty resolving small RF shifts, but no quantitative limit is given. Please report representative values for the minimum detectable frequency shift in prior methods—and the determining factors (loop bandwidth, discriminator slope, frequency-noise spectral density)—and then show by how much the present method surpasses that limit. Even in the Introduction, a quantitative benchmark for "resolving small RF shifts" would help.

4. Path to an on-chip spectrometer and thermal shift handling
Please outline how the device will be used as an on-chip spectrometer—for example, (i) an array of MEMS resonators with different absorption peaks for reconstructive spectroscopy, or (ii) a tunable element for scan-based spectroscopy. In addition, while the incident light changes the reactance via heating, does the absorption peak of the structure itself shift? If so, wavelength and intensity will couple, making spectral reconstruction nonlinear and requiring correction or inversion methods. If, under the intended operating flux, this effect is negligible, please state the evidence (e.g., a power sweep showing negligible peak shift).

Version 1:

Reviewer comments:

Reviewer #1

(Remarks to the Author)
The authors have addressed most of my concerns and have convinced me that the paper can be published in Nature Communications, as it presents, for the first time, a reflectometric readout of an RF-MEMS IR detector. This is particularly useful for high-frequency piezoelectric resonators. The only remaining issue I have is the discussion of responsivity vs. NEP (signal vs. SNR) as the key figure of merit. I recommend publishing the manuscript. I would, however, recommend that this issue be addressed in a revised version.

Here are some concrete points:

- Responsivity in units [V/W] is not a measure of sensitivity without knowing the corresponding noise. On the one hand, the signal could be amplified or depend on a bias voltage, boosting the value without improving sensitivity. On the other hand, the noise could be reduced, e.g., through parallel measurements (possible if the noise is uncorrelated), thereby improving sensitivity while the responsivity remains constant. The sensitivity of an IR detector is given by the NEP (you are correct that D* does not apply for thermal detectors, but only as long as they are not operated at the temperature fluctuation noise limit).

- On line 36, you mention that a high responsivity could lift the sensitivity into the BLIP regime. BLIP applies only to photodetectors, not to thermal detectors, because it usually implies reaching the shot noise limit (which is not possible with RT thermal detectors). And, to make my point about signal vs. SNR: every BLIP discussion in IR detector textbooks (e.g., Rogalski) is and has to be conducted by the NEP or D*, representing the SNR of the detector. Only the SNR can tell if a detector is operating in the BLIP regime; the responsivity alone can not.

- On line 44 and subsequent lines, it is correctly mentioned that the absolute IR responsivity, in units of [Hz/W], of MEMS/NEMS resonators scales with the resonance frequency. But the absolute IR responsivity in [Hz/W] is not an indication of performance. It is fractional resonant frequency changes that matter, which are typically characterized by the fractional Allan deviation of an oscillator, in units of [1/W]. This is similar to the signal vs. SNR discussion.

- On line 58, the authors mention that "the performance limits of RF MEMS-based IR sensors remain unquantified". I disagree with this statement. There have been many papers published in recent years, among them those from the St-Gelais group in Ottawa, Cleland and Roukes, etc, that clearly lay out the physical limits of resonant IR detectors. The fundamental limits of these detectors are temperature fluctuation noise and thermomechanical noise, which have both been clearly understood and experimentally demonstrated

Reviewer #2

(Remarks to the Author)
While I find the responses satisfactory and recommend acceptance, I note that some of the key clarifications provided in the rebuttal would strengthen the paper if partially reflected in the main text.

Version 2:

Reviewer comments:

Reviewer #1

(Remarks to the Author)
The authors have addressed all my remarks in depth, and I find the manuscript ready for publication in its current form.

Open Access This Peer Review File is licensed under a Creative Commons Attribution 4.0 International License, which permits use, sharing, adaptation, distribution and reproduction in any medium or format, as long as you give appropriate credit to the original author(s) and the source, provide a link to the Creative Commons license, and indicate if changes were made.
In cases where reviewers are anonymous, credit should be given to 'Anonymous Referee' and the source.
The images or other third party material in this Peer Review File are included in the article's Creative Commons license, unless indicated otherwise in a credit line to the material. If material is not included in the article's Creative Commons license and your intended use is not permitted by statutory regulation or exceeds the permitted use, you will need to obtain permission directly from the copyright holder.
To view a copy of this license, visit https://creativecommons.org/licenses/by/4.0/

We would like to thank the Reviewers for their time and valuable comments which greatly helped us to improve the quality of our manuscript. In this document we answer in detail each of the comments and describe the corresponding changes made to the manuscript.

Legends

This document uses the following color codes:

• Black: original comments of the reviewer.
• Blue: response from the authors.
• Red: changes introduced in the manuscript.

Reviewer #1 (Remarks to the Author):

—--

The manuscript presents a MEMS-based infrared (IR) sensing concept that combines a state-of-the-art piezoelectric resonator with LC-matched RF reflectometry and phase-sensitive detection. The claimed novelty lies in achieving high responsivity and in reading out IR-induced thermal frequency shifts through the phase change of a reflected RF tone, combined with an open-loop phase detection scheme, similar to conventional frequency-tracking methods such as lock-in detection. The authors further propose that the sensor's responsivity can be electrically tuned by adjusting the loaded Q-factor of the RF matching network.

We would like to thank the Reviewer for the insightful comments and suggestions that have undoubtedly helped us to increase the quality of our manuscript. We feel that many comments, in particular those claiming lack of novelty, have appeared due to a misinterpretation of some concepts introduced in our work - probably due to a somewhat confusing notation used in the previous version of the manuscript. We have revised the text to better articulate the operation mechanism and novelty of our IR sensing system, and to clarify the fundamental differences with respect to the state of the art.

<u>Major Comments</u>

- Figures of merit and comparison to state-of-the-art detectors

We have split our answer to this main comment into various sections, following the Reviewer's discussion.

The authors emphasize responsivity as the key performance metric and even highlight it in the title. I strongly disagree with this notion. For meaningful comparison with other IR detectors, whether electrical or mechanical, the relevant figures of merit are the noise-equivalent power (NEP) and the time constant. These two parameters together define a detector's sensitivity and speed independently of the specific readout configuration.

Responsivity (in V/W or A/W) is only appropriate for purely electrical detectors where the noise is also electrical (e.g., Johnson noise or 1/f noise). Even in such cases, responsivity alone does not characterize performance, as it ignores the noise level and thus the signal-to-noise ratio. For mechanical detectors, where thermomechanical noise often dominates, the transduction from frequency or displacement to voltage is irrelevant to the detector's intrinsic limits.

The performance of an IR sensor depends on many parameters and constraints. We fully agree with the Reviewer that the noise-equivalent power (NEP) and time constant are key figures of merit defining an IR detector's intrinsic limits. However, other parameters like responsivity, dynamic range, area, readout, integration, scalability, tunability, and cost are critical in IR sensing applications and should not be overlooked.

The Reviewer points out that responsivity is an appropriate performance metric for detectors where the noise is also electrical, i.e., 1/f noise. In fact, in our proposed reflectometric approach, the main noise component is the electrical 1/f flicker noise [see Eq. (4) in the main paper]. This electrical noise has been modelled theoretically (see Supplementary Information section 2.2) and confirmed experimentally (see Fig. 3 in the manuscript). We would like to stress that, even though our proposed platform relies on a thermal detector, thermomechanical noise is not dominant and thus the transduction from phase variations into voltage is indeed relevant to the detector limits. A detailed study on noise components can be found in our manuscript as well as in Supplementary Information, Section 2. As a result, and in agreement with the Reviewer's technical remarks, we believe that responsivity is an adequate metric to characterize the performance of our proposed platform. In our manuscript, responsivity is provided together with a careful study of noise, bandwidth, dynamic range, and tunability to fully characterize the proposed technology.

Furthermore, we respectfully advocate that responsivity remains a critical metric for system-level performance and realistic applications. In practice, a detector may possess an exceptionally low intrinsic NEP, but if its responsivity is insufficient to overcome the noise floor of the necessary readout electronics (LNAs, phase comparators, PLLs) and the unavoidable technical noise (~1/f) present in portable platforms, that intrinsic limit becomes far from attainable. Consequently, the system sensitivity becomes limited by the readout configuration and technical noise rather than the detector itself. In this context, high responsivity is the essential mechanism that lifts the detector's signal above the electronic noise floor, ensuring that the realizable system NEP approaches the fundamental limits the Reviewer correctly prioritizes. Thus, while the fundamental detector NEP defines the intrinsic limit, responsivity determines our ability to reach it in a real-world measurement and enables practical applications. This is particularly important in integrated, scalable, CMOS-compatible, and portable sensing platforms in which intrinsic electronic noise (1/f) is unavoidable and difficult to cancel out.

Additionally, developing IR sensors with giant responsivity will allow them to operate in the so-called background limited regime (BLIP) in which the detector's responsivity to background ambient fluctuations exceed the intrinsic detector noise. In this operation regime, usually attainable at cryogenic temperatures[1,2], the detector NEP and other noise components lose their relevance because background fluctuations dominate the sensor response. Thus, there is a clear

need to develop IR sensors with giant responsivity values that can eventually achieve BLIP operation at ambient conditions.

In this context, recent works have focused on seeking new approaches to enhance IR responsivity at room temperature - for instance, using new properties found in 2D materials[3-7] - despite challenges arising in terms of noise, reproducibility, and scalability. Our manuscript introduces a new approach that can be applied to drastically enhance the responsivity and performance of a whole family of RF MEMS-based IR sensors while addressing these shortcomings.

While we believe responsivity is a relevant metric, we understand that the language used in the introduction and title may give the reader the impression that we are ignoring NEP and the impact of noise. To address the Reviewer's valid concerns and also account for our discussion above, we have made the following modifications to our manuscript:

- In the Introduction, first paragraph, we have added the sentence:

Taken together, these limitations constitute a key bottleneck in the development of scalable, cost-effective, and on-chip IR spectroscopy and sensing systems[1,2,16]. These systems require moderately fast, low-noise, and spectrally selective detectors whose IR responsivity overcomes both the readout noise floor and the dominant $1/f$ noise of ambient electronics, enabling operation near their intrinsic NEP.

- In the second paragraph of the Introduction, we have expanded our discussion regarding NEP and bandwidth of previously reported sensing platforms. Additional details are provided in our answer to the Reviewer's next comment.

- In the third paragraph of the Introduction, we have provided more details regarding the 1/f noise component that dominates the proposed sensing platform. Specifically:

We also show that flicker 1/f noise dominates the platform's response and that it is inversely proportional to the device reflection coefficient.

- At the end of the first paragraph of the Introduction, we have discussed why increasing responsivity of IR systems is important to achieve operation in the BLIP regime.

Achieving even larger IR responsivity values would push these systems into the background-limited performance (BLIP) regime. Under BLIP conditions, usually attainable at cryogenic temperatures[1,2], background-induced fluctuations dominate the detector response and intrinsic noise components contribute negligibly to the sensor performance metrics.

Following this logic, the NEP reported here does not surpass that of existing MEMS/NEMS IR detectors. Consequently, the manuscript's impact is limited. When normalized to detector area, the specific detectivity of the presented device (D* ~ 2e7 Jones) is two orders of magnitude below state-of-the-art values (D* > 1e9 Jones), as demonstrated, for example, in Ref. 25, in Nano Letters

2025, DOI: 10.1021/acs.nanolett.5c03415, or in APL Photonics 2024 10.1063/5.0238977, where the latter also features a metamaterial absorber offering a defined spectral absorption.

We appreciate the Reviewer directing our attention to the 2025 Nano Letters and 2024 APL Photonics works, as well as emphasizing the reported metrics in Ref. 25. All three of these works report impressive specific detectivity (D*) values exceeding >1e9 Jones together with a limited bandwidth of just a few Hz. While we acknowledge the high intrinsic performance of the detectors in the provided references, we respectfully assert that it is not appropriate to *only* use detectivity to compare the performance of the detectors and their suitability to enable IR sensing applications. As further discussed below, key concepts to consider include (i) relevance of detectivity or NEP as a figure of merit; (ii) NEP and bandwidth; and (iii) integration and scalability. We note that our work did not claim to exhibit record-high detectivity levels.

First, while we recognize that D* is a common figure of merit for comparing IR detectors, we specifically avoided reporting D* for our detector given the uncertainty in the applicability of D* to this specific technology. The main difference between our work and the large-area IR detectors in the cited papers lies in the dominant fundamental mechanism that limits the NEP. More specifically, D* is a normalized metric derived under the assumption that NEP scales with the square root of the detector area, a relationship that holds true for photon-noise-limited detectors or radiation-limited thermal detectors. For instance, the devices in the works cited by the Reviewer operate in the radiation-dominated limit due to their larger areas. However, for the reflectometric MEMS-based IR sensors presented in this work, the mechanisms that limit the NEP are not directly related to the detector area. In fact, the dominant noise component is the electronic 1/f flicker noise that depends on the detector composing materials, geometry, and matching to the feeding transmission line [see Eq. (4) in the main paper, and Supplementary Information Section 2.2]. Other non-dominant noise components depend on the MEMS's thermal resistance (set by the anchor geometry and its composite materials) and the ambient temperature, rather than the device area. A detailed discussion on the noise components can be found in the Supplementary Information, Section 2. Consequently, applying an area normalization to calculate D* for our device introduces an ambiguity that penalizes the compact footprint of our sensor without reflecting its NEP performance. Therefore, while we acknowledge the high-performance of the referenced works, we maintain that NEP remains the most physically rigorous metric for characterizing the performance of the IR sensing technology developed in our work.

Second, NEP and bandwidth are important parameters to consider when comparing the performance of different IR detectors as they will determine their potential application in practice. In our work, the device bandwidth is ~ 288 Hz and the dominant noise component is electrical (1/f), with a measured NEP of 600 pW/Hz$^{-1/2}$ at 800 Hz and a predicted level still under 1 nW/Hz$^{-½}$ at 2 kHz. A key aspect of our work is that we measured low-power IR signals down to levels similar to the detector's predicted NEP - see Figure 4 in the main manuscript. Our experiments are performed at room temperature and ambient conditions using an integrated and scalable platform. Note that this detector was employed to illustrate the reflectometric approach, and, as discussed in the paper, there are still many options to further enhance its overall performance.

We are confident that this technology is ready to be employed in a wide variety of civil and military portable IR applications.

In the reference cited by the Reviewer, the IR sensor possesses a low thermal bandwidth of just a few Hz (~ two orders of magnitude lower than the detector shown in our work) and their noise is limited by the thermal noise of the mechanical resonator (NEP of 5 and 800 pW/Hz$^{-1/2}$ at 2 Hz and 800 Hz, respectively). Ultra-low NEP values are achieved at the cost of drastically reducing the operation bandwidth. This restricts the application of this technology to detect quasi-static infrared signals. It should also be noted that other RF MEMS-based IR sensing platforms reported in the literature predicted a NEP of a similar order of magnitude, ranging from 10 to 50 pW/Hz$^{-1/2}$ at low frequency offsets[8-9]. It must be noted that, in most cases, NEP metrics are reported using partial experimental characterization techniques that may not fully capture complex mechanisms within the complete detection system and electronic readout - especially important when the power of the IR light approaches environmental noise levels. Thus, even though predicted, the actual performance of such devices in realistic and portable sensing platforms has not yet been fully demonstrated.

And third, the sensors described in the references pointed out by the Reviewer rely on an optical readout that includes a laser interferometer. Measurements are reported in a vacuum chamber. Such set-ups are well suited for delicate experiments within lab settings, but they are bulky, complex, expensive, and challenging to integrate within other systems and to scale for mass production. Thus, it is difficult to apply such technology in modern applications that must meet stringent requirements in terms of integration, portability, power, weight, and cost. In contrast, the approach proposed in our work can be applied to the entire family of MEMS-based IR sensors while being compatible with standard fabrication processes, miniaturized, scalable, and ready to be applied in portable IR sensing systems.

More broadly, our manuscript introduces a new approach to enhance the responsivity and overall performance of RF MEMS-based IR sensors. This is a general technique that is demonstrated in our manuscript through a specific CMR MEMS device. Note that this detector has not been optimized to achieve record low NEP performance - as this was not the purpose of our work. Following the Reviewer’s comments, the features of our reflectometric technique can be exploited to further manipulate the detector NEP and bandwidth for specific applications. To this purpose, it is important to keep in mind that the electrical 1/f flicker noise dominates the response of this technology. It is well-known that flicker noise decreases with larger device areas[10], as bigger body sizes average out random fluctuations that originate this type of noise. On one hand, scaling down the size of the MEMS body would increase the overall noise and NEP but it would also increase the detection speed (thermal bandwidth) and facilitate the development of integrated and miniaturized arrays of sensors that target different IR bands for spectroscopy applications. On the other hand, larger MEMS body areas would lead to a much lower overall noise and a NEP approaching the theoretical fundamental limits of the device as discussed in the Supplementary Information, Section 2. The price to pay would be a reduced thermal bandwidth and associated detection speed. The MEMS device employed in our manuscript represents a trade-off between the two options discussed above. Optimized responses can also be obtained by increasing the

device thermal resistance by optimizing the MEMS anchors or using better performing RF phase comparators. Moving beyond, the reflectometric approach introduced in this manuscript is general and can be applied to other MEMS technologies, like FBAR, SiN, different types of CMRs, etc that might greatly outperform the response of the CMR MEMS-resonator employed here for the sake of demonstration.

To account for the Reviewer comment and our response, we have modified the manuscript as follows:

- In the second paragraph, we explicitly mention the NEP of other works reported in the literature:

To date, the operation principle of RF MEMS-based IR sensors has relied on monitoring frequency shifts of specific mechanical modes upon IR illumination[28], using either optical[22-26] or electrical readouts[17-21], yielding NEP values down to ~ $\mathrm{pW}/\sqrt{\mathrm{Hz}}$ levels.

- In the second paragraph, we discuss the trade-off between NEP and bandwidth in RF MEMS-based IR sensors:

In these devices, IR responsivity directly scales with the MEMS RF operation frequency and thermal resistance whereas the device size determines speed and bandwidth. Despite promising performance, several challenges have hindered a broader adoption of this technology. First, integrating readout mechanisms on-chip remains a challenge: optical schemes require bulky interferometric setups incompatible with chip-scale platforms and electrical readouts must resolve small RF shifts. This latter challenge imposes a performance trade-off in this technology. Devices operating at low RF frequencies usually offer limited bandwidth and IR responsivity but achieve ultralow NEP values by using precise frequency tracking based on phase-locked loop (PLL) circuits[28]. High-frequency RF MEMS, operating at GHz or 100s of MHz, provide larger responsivity and faster response. Nevertheless, PLL tracking becomes constrained at these frequencies by an elevated phase noise that increases NEP.

- In the Discussion section, first paragraph, we added a sentence to further discuss the trade-off between NEP and bandwidth in RF MEMS-based IR sensors:

A trade-off between noise and bandwidth arises: larger (smaller) MEMS structures will lead to reduced (enhanced) flicker noise contributions[44] and reach lower (higher) NEP values at the cost of slower (faster) responses.

I find it commendable that the authors follow a well-defined and rigorous procedure to characterize their detector's performance, as outlined in Ref. 5. This is good scientific practice, and future studies in the field would certainly benefit from adopting similarly standardized methods. However, I do not agree with the authors' implication that earlier works lacking such systematic characterization are, therefore, in essence, not trustworthy. The introduction's rather sweeping critique of prior studies is unjustified; many of those reports have demonstrated meaningful advances in detector performance, even if their evaluation protocols were less standardized.

We sincerely thank the Reviewer for their positive comments regarding the experimental rigor of our work. We apologize if the tone of the introduction came across as a harsh sweeping critique of previous studies. It was not our intention to imply that works lacking the same level of systemic characterization are therefore untrustworthy.

Our intent with the introduction was to highlight what we feel is a very relevant distinction: that it is one thing to approximate and predict low-power detection, but it is an entirely different and significant challenge to experimentally measure and validate those predictions.

The claims summarized in the introduction about the need for rigorous characterization of IR detectors are not only our own - similar concerts can be found in the conclusions and commentary from other works[4,11]. By adopting a rigorous frequency-dependent responsivity, noise, and NEP characterization that also incorporates IR spectral selectivity and power-control (i.e., dynamic range and saturation), we sought to address these known criticisms. We feel the demonstration of a measured low-power IR signal (Figure 4 of our main paper) adds to the novelty of our work as it provides experimentally validated proof of an achievable NEP rather than a prediction that might not be achievable in practice.

Since, in our view, the rigorous characterization of IR detectors in our work should be highlighted, some discussion of the criticisms raised by other works[4,11] in the introduction is warranted. Nonetheless, we certainly acknowledge that potential readers may view the introduction in its current form as a harsh criticism of prior works and implies that other works which do not use the same detailed characterization are unreliable. To remedy this harsh tone in the introduction, we have made the following modifications to the second paragraph of the Introduction to make it clearer we are providing solutions to raised criticisms and are not implying that results in other works are incorrect:

Second, many studies have emphasized the development of key device innovations instead of focusing on systematic sensing characterization procedures[5]. Figures of merit such as responsivity and NEP are often presented without considering their strong dependence on IR wavelength, power, or modulation frequency, leading to performance metrics that may not reflect realistic operation conditions. As a result, the performance limits of RF MEMS-based IR sensors remain unquantified and their potential for chip-integrated IR spectroscopy largely untapped.

- Novelty of the readout concept

The proposed RF reflectometry scheme essentially constitutes an open-loop implementation with phase detection. The phase of the reflected signal is monitored at a fixed frequency to infer small resonance shifts. This approach is functionally equivalent to the phase error signal used in lock-in detection, e.g., in a phase-locked loop (PLL), a well-established closed-loop frequency-tracking method based on phase comparison.

Therefore, the “phase comparison” technique presented here does not represent a new sensing or readout principle; it is simply the open-loop analog of conventional PLL-based frequency demodulation. Moreover, such open-loop operation will likely be challenging to implement in

practice, as the resonance frequency of the highly temperature-sensitive MEMS resonator will drift with environmental variations.

We do not agree with this assessment. In the following, we describe in detail how the proposed reflectometric-based readout system is novel in the context of IR sensing, relies on an operation principle different to the common frequency-tracking employed in RF-MEMS, requires an implementation that though somewhat similar is not equivalent to the one found in open-loop PLL detection, and significantly enhances the sensor performance. We thank the Reviewer for the comment, which allows us to clarify this important point both in this document and in the manuscript.

We begin by recalling that, from a fundamental perspective, the incident IR power modifies the impedance of an RF MEMS-based sensor which in turn changes its resonance frequency. To date, MEMS-based IR sensing has relied on tracking the resonance frequency of the device. However, tracking the changes of the detector's input impedance versus IR light have been mostly overlooked. The resonator's input impedance is a complex and frequency-dispersive parameter that provides richer information than simple resonance frequency tracking. Tracking changes in the detector input impedance is a general concept that can be applied to RF MEMS-based IR sensors implemented with any technology, including FBAR, CMR, SiN, etc., regardless of size and operation frequency.

In our manuscript, we propose reflectometric RF MEMS-based IR sensors. In RF reflectometry, the main goal is to accurately determine changes in the input impedance of a circuit, material, or sensor. Specifically, ***we monitor variations of the detector input impedance versus IR power and translate them into sensing parameters such as responsivity and noise***. Additionally, it is well-known in reflectometric readouts that manipulating the effective quality factor of the device significantly improves the detection performance[12-13]. Inspired by reflectometry, we incorporated a matching network to the MEMS resonator to control the effective quality factor of the IR device. This parameter could be interpreted as the loaded quality factor of the MEMS + matching network circuit. We emphasize that this effective quality factor is defined through the device scattering parameter S11 phase-frequency slope[14] and accounts for the MEMS, a matching network, and the interconnection to an external transmission line. In this context, our work goes well beyond putting together a MEMS sensor and a reflectometric readout: we analytically demonstrate that the device IR responsivity and effective quality factor are directly proportional [Eq. (2) in the manuscript], and thus the matching network becomes a key part of the intrinsic sensor performance. We exploit this fact to boost and manipulate the detector IR responsivity by using tunable matching networks. Note that even though reflectometric approaches are well-known in the literature[15,16], they have not been previously applied to IR sensing.

In the comment, the Reviewer specifically discusses approaches to track the frequency shift of the MEMS resonator versus incoming IR light using PLLs. In a PLL, the system actively adjusts the drive frequency to follow the device resonance frequency. This approach is very well suited for MEMS devices resonating at low frequencies, usually at kHz and below, a regime in which the PLL's phase noise is lower than the intrinsic noise of the MEMS resonator[17,18]. For this reason,

PLL-readouts are excellent for low-frequency MEMS-based sensors. However, these sensors are usually relatively large in size and offer a limited thermal constant that hinders their sensing speed[18] coupled with a moderate responsivity - note that responsivity is directly proportional to the MEMS operation frequency, see technical details in Supplementary Information, Sections 1 and 2 and Eq. (S9) therein. As frequency increases, the PLL's phase noise increases (scaling with 20log(N) where N is the frequency multiplication factor) and may become dominant. This might explain why PLL-assisted readouts have not yet been employed in RF MEMS-based IR sensors that operate at MHz and GHz frequencies, such CMR[19-20] and FBAR[21] structures. These devices offer larger IR responsivity and reduced footprint associated with a much faster speed. In the literature[22], several techniques designed specifically for tracking the resonance frequency of MEMS/NEMS resonators are compared. They demonstrate that monitoring the phase of a fixed-frequency reflected RF signal, an open-loop method, can detect small resonance shifts with high sensitivity. The study further notes that while open-loop phase detection is conceptually similar to the phase error in a PLL, it avoids the complexity of closed-loop frequency tracking, and even "feedback-free" schemes can achieve performance comparable and superior to PLLs under certain conditions. Still, these approaches determine the resonator frequency shift upon IR illumination and all lead to the same IR responsivity as previously reported in the literature. Conceptually, this frequency-shift tracking is fundamentally different with respect to the reflectometric approach introduced in our manuscript.

Finally, thermal-based IR detectors are generally susceptible to environmental fluctuations. In this context, the concept of thermally optimized MEMS packaging is well-developed and has been extensively explored in both research and commercial applications[23,24]. Over the past two decades, advances in wafer-level packaging, hermetic sealing, and material engineering have enabled MEMS devices with highly stable thermal performance, even under fluctuating ambient conditions. Techniques such as silicon or glass cavity encapsulation, low-CTE package materials, and suspended MEMS structures are now standard in precision sensors, including accelerometers, gyroscopes, and RF resonators. Additionally, active thermal stabilization using on-chip heaters and temperature sensors has been implemented in specialized applications requiring extreme thermal stability, such as infrared detectors and atomic force microscopy. Therefore, we believe that potential environmental shifts in the resonance frequency can be well accounted and compensated for. Additionally, reflectometric MEMS-based IR detectors have a tunable matching network that can also partially compensate for such effects. Note all experimental data reported in our manuscript has been collected at room temperature and ambient conditions.

To further highlight the novelties of the proposed readout circuit, and address the Reviewer's comment, we have revised the manuscript as follows:

- We have updated the Abstract. It now reads as:

Radiofrequency microelectromechanical systems (RF MEMS) decorated with metasurfaces have emerged as promising spectrally-selective infrared (IR) sensing platforms. Upon IR illumination, these devices modify their effective motional capacitance driving a measurable shift in their RF resonance frequency. To date, MEMS-based sensors operate by tracking such frequency shifts

using electrical or optical readouts. Here, we apply RF reflectometry to track changes in the RF MEMS input impedance and analytically relate these variations to fundamental sensing metrics such as responsivity and noise. The resulting detector, composed of a reconfigurable matching network coupled to an RF MEMS, exhibits an intrinsic IR responsivity governed by its loaded quality factor and tunable up to exceptionally large values. The platform, demonstrated at ambient conditions using contour mode resonator (CMR) MEMS, achieves a responsivity >11,400 V/W in a 50-Ω readout circuit (>200 A/W), spectral selectivity with a full-width half maximum (FWHM) of 0.54 μm centered at 5.94 μm, noise equivalent power (NEP) of $\sim 450\ \mathrm{pW}/\sqrt{\mathrm{Hz}}$, a time constant of $\sim 552\ \mu\mathrm{s}$, and resolves low-IR power levels down to ~ 740 pW. By combining reconfigurability, spectral selectivity, and ultra-high responsivity with scalable fabrication, reflectometric RF MEMS IR detectors open new avenues toward high-performance on-chip IR spectroscopy and sensing platforms.

- We have incorporated the discussion about PLL for frequency-tracking in the second paragraph of the Introduction. The sentences added read:

In these devices, IR responsivity directly scales with the MEMS RF operation frequency and thermal resistance whereas the device size determines speed and bandwidth. Despite promising performance, several challenges have hindered a broader adoption of this technology. First, integrating readout mechanisms on-chip remains a challenge: optical schemes require bulky interferometric setups incompatible with chip-scale platforms and electrical readouts must resolve small RF shifts. This latter challenge imposes a performance trade-off in this technology. Devices operating at low RF frequencies usually offer limited bandwidth and IR responsivity but achieve ultralow NEP values by using precise frequency tracking based on phase-locked loop (PLL) circuits[28]. High-frequency RF MEMS, operating at GHz or 100s of MHz, provide larger responsivity and faster response. Nevertheless, PLL tracking becomes constrained at these frequencies by an elevated phase noise that increases NEP.

- We have updated the third paragraph of the Introduction to highlight the novelty of our proposed approach. It now reads as:

Here, we introduce a new paradigm for high-responsivity IR sensing based on tracking the variations of the RF MEMS input impedance at a single RF frequency and mapping them into sensing performance metrics. To this purpose, we merge RF MEMS-based IR sensors with RF reflectometry[29-32] - a technique that enables highly sensitive detection of reactance variations by monitoring the phase shift of a reflected RF tone and that can operate up to RF frequencies well into the GHz regime. In our approach, the IR detector is composed of an electrically tunable matching network coupled to the MEMS resonator. The device is excited at resonance with an RF tone, and the phase delay of the reflected signal is measured in a differential configuration as a function of the incident IR radiation. The output voltage is delivered in a $50\ \Omega$ circuit, ensuring compatibility with standard readout electronics. We show both theoretically and experimentally that responsivity scale linearly with the device's loaded quality factor thus enabling reconfigurable and high-performing IR sensing.

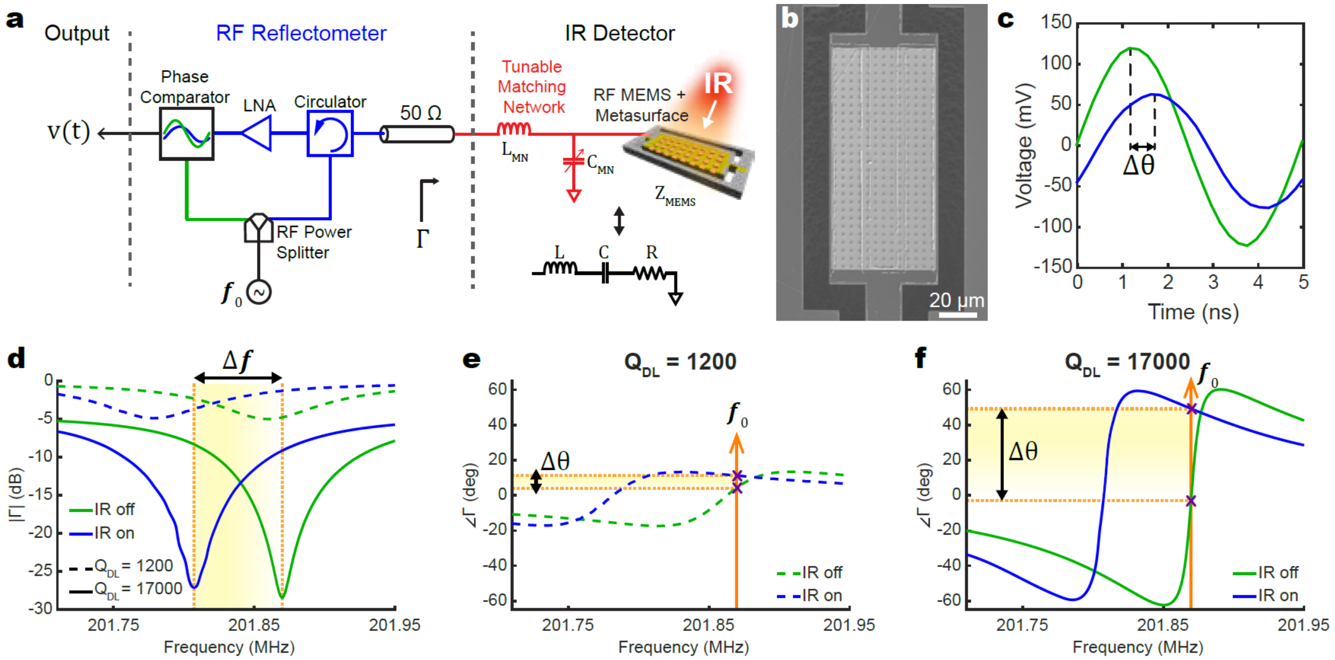

Fig. 1. Operation principle of reflectometric RF MEMS-based IR detectors. a. Schematic. The detector is composed of an AlN CMR RF MEMS resonator (decorated with an IR absorptive metasurface) that is coupled to a tunable LC matching network. The MEMS is characterized with an impedance Z_{MEMS} via a Modified Butterworth-Van Dyke (MBVD) network. Around resonance, the detector is modelled using an equivalent RLC resonator network that possesses a loaded quality factor Q_{DL}. The reflectometric readout is composed of a generator that supplies an RF signal at f_0 that is split into two paths: one feeds the IR detector and is reflected back through a circulator (blue line) and the other serves as a reference (green line). An RF phase comparator generates a voltage signal $v(t)$ that is proportional to the phase difference between the two RF tones. The detector is illuminated with a coherent IR beam that is tunable is terms of wavelength and power (see Supplementary Information). **b.** Scanning electron microscope image of the RF MEMS. The sensing area is 140 μm x 60 μm. **c.** Time-domain measurement of the RF tones serving as a reference (green line) and reflected from the MEMS (blue line). The loaded quality factor of the device is $Q_{DL} = 1200$, the frequency and power of the RF tone are set to 201.86 MHz and – 7 dBm, respectively, and the incoming IR beam at $\lambda_0 = 5.94\ \mu m$ has a total optical power $P_{IR} = 750\ \mu W$ on the sensing area. **d-e.** Reflection coefficient Γ in magnitude (d) and phase (e,f). Results are plotted when the device's quality factor is set to $Q_{DL} = 1200$ (dashed lines) and $Q_{DL} = 17{,}000$ (solid lines) considering no IR light (green lines) and a focused and coherent IR beam as described in panel **c** (blue lines). Circuit parameters are $R = 186.5\ \Omega$, $L = 297.3\ \mu H$, $C = 2.09\ fF$ for Q_L =1200, and $R = 56.89.8\ \Omega$, $L = 97.635\ \mu H$, $C = 6.383\ fF$ for $Q_{DL} = 17{,}000$.

- We have updated Fig. 1 to better clarify the operation principle of the proposed platform. The figure is also included in this document as a reference.
- In the last paragraph of the manuscript, we have highlighted the need of using appropriate package for this technology:

Arrays of reflectometric MEMS-based detectors integrated within a thermally optimized package, each targeting a distinct IR wavelength, provide a …

In addition, RF reflectometry using LC-coupled MEMS or NEMS resonators is not new. Similar approaches have been reported previously for capacitively coupled nanomechanical systems (see Nano Letters 2006, DOI: 10.1021/nl062278g; Nature 2014, DOI: 10.1038/nature13029).

Thank you for the comment. While RF reflectometry and applying it to MEMS/NEMS is certainly not entirely new, applying this approach to IR sensing is to the best of our knowledge **a new research direction.** Any change in the mass, damping, or stiffness of the MEMS resonator leads to a change in the resonator impedance, so there is a wide range of applications for reflectometric readout systems in RF MEMS-based detectors

In terms of the references cited by the Reviewer, the Nano Letters paper provides a useful and relevant introduction to RF reflectometry with MEMS. We consider it relevant and we have added it to our literature review. That work includes an equivalent circuit model, experimental demonstration, and extension of the readout to arrays of resonators. However, this paper does not focus or describe IR sensing, nor explore the control of the phase or amplitude of the reflected signals. In contrast, we demonstrate theoretically and experimentally that through RF reflectometry the intrinsic IR detector responsivity can be enhanced by adjusting the device's effective quality factor and we leveraged such response for high-performing IR sensing.

In the Nature reference, the LC circuit is tuned only to match the resonator frequency, aiming to boost weak RF signals for optical readout. The goal of the device is to detect RF signals optically. This approach is fundamentally different from ours, both in tuning strategy and in purpose. While that work also presents detailed noise analysis, it differs from our analysis because the device operation and sensing principle are fundamentally different.

For additional context, note that using matching networks in frequency-tracking approaches in RF MEMS allow to enhance weak signals and simplify the experiments in practice, but the detector's responsivity (frequency shift change upon IR light) does not change. **This scenario is fundamentally different in our proposed reflectometric approach**: the intrinsic IR sensor's responsivity is directly proportional to its effective quality factor [Eq. (2) in the manuscript]. As discussed above and demonstrated in our manuscript, this response arises because reflectometry determines impedance variations of the IR detector instead of tracking its frequency shift upon IR light. Thus, the RF reflectometry approach described here drastically differs from prior work in the context of IR sensing and leads to new perspectives and possibilities in the field of MEMS-based IR sensors.

It is also unclear how a loaded Q greater than the intrinsic Q of the resonator could be achieved. When the electrical (RC) and mechanical resonators couple, especially in a piezoelectric device, the resulting system should exhibit a total Q limited by the most lossy component, which here is the mechanical resonator (Q = 2200). A more detailed discussion of this point would be helpful.

We thank the Reviewer's comment and acknowledge that the notation previously employed might have been confusing. We completely agree that the loaded Q of a resonator should not exceed the intrinsic one. Here, we address in detail the Reviewer's concern and detail our changes in the manuscript.

In the proposed reflectometry system, the effective quality factor Q_{DL} accounts for the input impedance of the RF MEMS, potential mismatch to the connecting transmission line, and matching network. It can be interpreted as the loaded quality factor of the MEMS + matching network circuit. Analytically, we defined it through the phase-frequency slope of the S11 parameter (see Supplementary Information, Section 1 and Refs.[25-26]). Note that it is physically meaningless to compare the effective quality factor of the entire device (loaded Q of MEMS+matching network) with the loaded/unloaded quality factor of just the MEMS resonator, and that the former can be drastically larger than the latter.

In our proposed system, the IR detector's responsivity depends on the effective quality factor of the device Q_{DL} together with other parameters such as the thermal resistance and the TCF (see Eq. (2) of the manuscript, and the detailed derivation in the Supplementary Information, Section 2). Thus, it is possible to enhance the intrinsic IR sensor performance by manipulating a matching network to boost Q_{DL}. Similar responses in different application contexts are well-known in reflectometric systems: tiny variations of input impedances can be determined with high sensitivity by judiciously adjusting the overall matching of the system[13, 16]. However, this is not the case in RF MEMS-based IR sensors that rely on tracking the device resonance frequency. In these detectors, the IR responsivity does not depend on the device's effective quality factor or even on the mechanical quality factor of the MEMS resonator. Instead, IR responsivity only depends on the device operation frequency, thermal resistance, and TCF (see Eq. (S9) in the Supplementary Information). This is a fundamental difference between our reflectometric IR sensing platform and previous approaches.

To account for the Reviewer's comment and our response, we have modified the manuscript as follows:

- We have updated Fig. 1 to better illustrate the response of our proposed platform. As a reference, it is included as Fig. 1 in this document.

- We have updated the first paragraph under the Results section as follows:

The RF MEMS resonator considered here is shown in Fig. 1b and consists of a 1 μm-thick aluminum nitride (AlN) CMR, with resonance frequency $f_0 = 201.86$ MHz an unloaded quality factor $Q_{MU} = 2200$, integrated with a metasurface targeting IR absorption at $\lambda_0 = 5.94$ μm (Methods). The reflectometric detector is constructed by coupling a tunable matching network to the RF MEMS, as shown in Fig. 1a. Near its resonance, the detector response can be accurately described by an equivalent RLC circuit, characterized by a loaded quality factor Q_{DL} that is tunable via the matching network. Figures 1d–f show the frequency-dependent magnitude and phase of the detector reflection coefficient for two representative values of Q_{DL} (green curves). In our quality-factor notation, subscripts M, D, L, and U correspond to MEMS resonator, detector, loaded, and unloaded, respectively.

Minor Comments

- Spectral absorptance and application context

The manuscript highlights the advantage of a narrow spectral absorptance but does not specify where such a characteristic would be particularly useful. A clear application example would strengthen the argument. For instance, narrowband detection is beneficial for gas sensing, but would be impractical for spectroscopy, which typically requires hundreds or thousands of resonators to achieve broadband spectral coverage for mid-IR fingerprinting of unknown samples.

Narrowband IR detection is particularly valuable in applications where high sensitivity is required only within a *limited and known spectral bandwidth*. As pointed out by the Reviewer, narrowband detection is already a cornerstone of gas sensing, where tunable mid-IR and near-IR laser-absorption techniques target specific molecular absorption lines to achieve high-sensitivity trace-gas detection, making selective, wavelength-matched detectors inherently advantageous for these applications. Additional applications where narrowband spectral absorptance would be advantageous include active imaging, multispectral/hyperspectral imaging, target designation, free-space communications, laser spectroscopy for chemical/biological/explosives sensing, laser/beacon detection, and Light Detection and Ranging (LiDAR)[27]. These systems routinely operate at discrete wavelengths set by a laser source, a priori spectral signature, or an engineered filter set; therefore, a detector with an intrinsically narrow FWHM improves selectivity, reduces background loading, and eliminates the need for external spectral filtering.

Example applications include:

- **Multispectral and hyperspectral imaging.** For instance, multispectral IR imaging of microbial colonies using QCL illumination at nine discrete wavelengths has been shown to extract both morphological and biochemical information, enabling machine-learning-based classification[28]. Similarly, "algorithmic spectrometry" using plasmonic filter arrays has demonstrated that a small set of narrowband channels can reconstruct unknown LWIR scenes[29]. In these systems, each pixel benefits from a well-defined, narrow absorption band rather than broadband response.

- **Laser spectroscopy.** Tunable mid-IR lasers (such as quantum-cascade and difference-frequency sources) enable high-sensitivity and high-selectivity detection of chemical species via their characteristic absorption lines. As shown by Sigrist, these techniques are used in trace-gas monitoring, analysis of surgical smoke, non-invasive in vivo glucose sensing using defined mid-IR lines and narrowband laser wavelengths[30].

- **LiDAR and active ranging.** In Ref. [31], narrowband photodetectors were demonstrated without external filters to enhance sensitivity in weak-signal LiDAR systems by removing losses due to optics. This is an approach directly aligned with the narrowband metasurface absorption of our platform.

- **Infrared spectral fingerprinting and pattern recognition.** Many IR-based classification tasks are increasingly combined with machine-learning frameworks, such as macrophage phenotyping via near-IR fingerprinting[32] and broader disease classification through spectral analysis[33], to aid in the faster resolution of complicated problems, particularly those that cannot be solved using traditional human heuristics. These methods rely on selected spectral features (e.g., peak intensities, wavelength centers) rather than continuous broadband spectra. A narrowband detection strategy that is tuned to engineered wavelengths corresponding to those discriminative features could therefore enhance classification accuracy and significantly reduce the complexity and cost of detector arrays.

We also agree with the Reviewer that narrowband absorptance is not suitable for *broadband* chemical fingerprinting of unknown samples, which requires many spectral channels. To avoid confusion, we now explicitly state that our platform is intended for *spectrally selective* measurements at one or a few targeted wavelengths, not full-spectrum reconstruction. We additionally clarify that future array-level implementations or utilization of tunable metasurfaces may enable multi-band operation without the need for hundreds of distinct resonators; this is explained in more detail in our reply to Reviewer 2.

We have not included the full answer to this Reviewer's question in the manuscript because it is already very long. We can easily do so if the editor recommends it. However, to partially account for this discussion, we have added the following sentence in the last paragraph of the IR responsivity based on RF reflectometry section:

Peak responsivity is achieved at 5.94 μm with a FWHM of 545 nm, providing spectrally selective detection without the need of external optical filters. Such intrinsic narrowband operation is well suited for sensing modalities that operate at discrete wavelengths, including molecular gas sensing, active imaging, and laser-based detection systems, where enhanced selectivity and reduced background loading are critical.

- References

We appreciate the Reviewer identifying and bringing to our attention the issues with the references. We have thoroughly checked the references throughout the manuscript and updated them as necessary, with changes highlighted in the document.

For the sake of completeness, the issues with the references pointed out by the Reviewer are also addressed below:

Several references are incomplete or incorrect:
Page 1: "…using either optical 22–25 or electrical readout 17,19,20."

The concept in Ref. 22 was later implemented as a NEMS array with electrical readout, achieving NEP = 27 pW/√Hz (Phys. Rev. Appl. 2018, 9, 024016).
Ref. 23 also describes a NEMS detector with electrical readout and NEP = 7 pW/√Hz.

We have fixed the references as suggested by the Reviewer. Note that we have also added new references, as previously discussed in this document.

Page 2: "…and has recently attracted attention for probing quantum devices 18,27."
Ref. 18 does not concern quantum devices; it describes an earlier version of the present detector.

Thank you for pointing this typo out. The citation of Reference 18 has been removed. We have added the reference:

> Vigneau, F. *et al.* Probing quantum devices with radio-frequency reflectometry. *Applied Physics Reviews* **10** (2023). https://doi.org/10.1063/5.0088229.

Page 2: "…while optical readouts require bulky interferometric setups incompatible with chip-scale platforms 22,23."
As noted above, both Refs. 22 and 23 actually employ electrical readout.

Since Reference 22 relies on an optical interference setup that cannot be easily integrated, we have chosen to keep this citation. The citation to Reference 23 has been removed and replaced with:

> Adiyan, U., Larsen, T., Zárate, J.J. et al. Shape memory polymer resonators as highly sensitive uncooled infrared detectors. *Nat Commun* **10**, 4518 (2019). https://doi.org/10.1038/s41467-019-12550-6

These are only a few examples; the authors should carefully check all references for correctness and completeness.

We apologize for these typos that appeared when finalizing the manuscript. We have verified, to the best of our knowledge, that the updated version of the manuscript is typo free.

- Page 3, eq. for the frequency shift:

Conventionally, the absorption coefficient is expressed in units [1/m]. The absorption defined here is rather the absorptance, a dimensionless quantity. For clarity, the authors should revise the notation and terminology accordingly.

The Reviewer is correct: the expressions in our manuscript and Supplementary Information do not rely on the absorption coefficient but on the absorptance $\eta(\lambda)$ - a dimensionless wavelength-dependent parameter. An absorptance with a value of 0 means that there is no absorption at that particular wavelength, whereas a value of 1 indicates complete absorption.

To account for this remark, we have modified 'absorption coefficient' to 'absorptance' wherever needed in the text.

- Page 3, thermal resistance Rth:

What is the dominant thermal loss mechanism in the system? Radiation, thermal conduction, or convection?

In the piezoelectric contour mode resonator employed in this work, which has already been well-characterized in the literature[34,35], the dominant thermal loss mechanism is conduction. Thermal conduction here occurs through the anchors (see Supplementary Figure S1) which tether the device to the wafer substrate and allow heat to escape through solid materials. Radiation is comparatively negligible due to the low environmental temperature and small device area. Similarly, convection is comparatively negligible due to small device area. The effective thermal resistance associated to each effect can be approximated as[34,35]

$$R_{th,eff,conduction} = \frac{L_{anc}}{\sum \kappa_i t_i \cdot W_{anc}},$$

$$R_{th,eff,radiation} = \frac{1}{A\epsilon\sigma(T^2+{T_0}^2)(T+T_o)},$$

$$R_{th,eff,convection} = \frac{1}{hA}.$$

In the above expressions, L_{anc} and W_{anc} represents the length and width of the anchors, respectively; κ_i and t_i are the thermal conductivity and thickness of the layer i; and A, ϵ, σ, T, and T_o represent the sensor area, emissivity, stefan-boltzman constant, temperature of the sensor, and environmental temperature, respectively. Finally, h represents the heat transfer coefficient. The expression for thermal resistance due to conduction accounts for multiple material layers in parallel. Feeding these approximate equations with the parameters of our fabricated device (see Supplementary Information) reveals that thermal resistances due to radiation and convection exceed conduction by well over an order of magnitude. Thus, we can safely conclude that our device is dominated by thermal conduction - in agreement with numerical simulations (see Supplementary Information) and the literature[34,35].

We have not included this discussion in the manuscript because it is already quite long, and this information is available in the literature. However, we have modified our manuscript to explicitly mention that thermal resistance in this specific device is dominated by thermal conductance through the anchors, and we refer the interested reader to appropriate references. Specifically, we have added the following sentence in the last paragraph of the Operation Principle section:

> In this type of MEMS, thermal resistance is dominated by the thermal conductance of the anchors[33-34].

- Page 4, eq.(4):

What is the maximum input RF voltage V0 that can be sent to the MEMS?
Does the RF drive contribute to self-heating, and if so, how does this affect the overall noise performance?

We thank the Reviewer for the comment. The absolute maximum input RF voltage, V_0, that can be sent to the MEMS before permanent damage varies from device to device but can be roughly stated to be >+10 dBm (~700 mV_{rms}). The edge of nonlinearity also varies by device and depends

on quality factor but can be qualitatively stated to be ~-5 dBm (~126 mV_{rms}), see Refs. [36, 37]. In our work, the MEMS is excited by -15 dBm (~40 mV_{rms}) of RF power. This is selected to retain a high SNR while sufficiently avoiding the nonlinear regime.

The RF drive does contribute to self-heating and noise; however, this dissipation-induced noise is not significant with respect to other noise components. Dissipation-induced phase noise has a $1/f^2$ characteristic[38]:

$$S_{\phi}(\omega) = \frac{k_B T}{8\pi P_c Q^2}\left(\frac{\Omega}{\omega}\right)^2 \ ,$$

where kb, T, Pc, Q, Ω, and ω are Boltzman's constant, temperature, RF drive power, unloaded quality factor of the MEMS, RF drive frequency, and frequency offset respectively. For the MEMS studied in this work, this expression predicts $S_{\phi}(f = 100\ Hz) \approx -177\ dBc/Hz$, far below the measured phase noise presented in the main text (Fig. 3).

To account for this comment and our response, we have modified the section "RF noise characterization" in Methods as follows:

For the sensing path, RF signal generated by a Keysight E8663D is employed to excite the IR detector at its resonance frequency with an RF power level of -15 dBm (~40 mVrms). Note that the absolute maximum input RF voltage, V0, that can be sent to the MEMS before permanent damage varies from device to device but can be roughly stated to be >+10 dBm (~700 mVrms). The onset of nonlinear responses also varies by device and depends on quality factor but can be qualitatively stated to be ~-5 dBm (~126 mVrms), see Refs. [53, 54].

Summary and Recommendation

The manuscript presents a technically sound engineering demonstration but lacks the conceptual novelty and physical insight expected for Nature Communications. The performance improvement over existing piezoelectric IR detectors (Ref. 17) is incremental rather than fundamental. The achieved specific detectivity is two orders of magnitude below the current state of the art.

I therefore do not recommend publication in Nature Communications.
The work would be better suited to a specialized MEMS or sensor journal, where solid incremental advances in device design and readout schemes are valued.

We thank the Reviewer again for the detailed feedback. In our reply, we have clarified the novelty of the proposed reflectometric approach and described how it can be applied to enhance the performance of the RF MEMS-based IR sensor family; contextualized that the responsivity value reported is outstanding with respect to the state of the art (>200 A/W) and very relevant in portable platforms in which 1/f technical noise is unavoidable; highlighted that the development of high-responsivity IR sensors is important to achieve operation in the BLIP regime at ambient conditions, and answered all other questions and concerns raised. We have incorporated all these changes in the updated version of the manuscript. We hope the Reviewer will reconsider their opinion with respect to the quality of our work and its suitability to Nature Communications.

Reviewer #2 (Remarks to the Author):
—---

This manuscript reports a room-temperature MIR detector with spectral selectivity. While RF reflectometry–based MIR detectors have appeared recently, they typically rely on tracking small frequency shifts and have shown limited performance. In contrast, the present work detects the reflection phase of an RF tone coupled to a MEMS resonator, establishing a new approach that enables high-resolution MIR measurement. The wavelength resolution is evaluated with a laser source, and the data convincingly support the claims and novelty. In my view, this paper is suitable for publication in Nature Communications, pending clarification of the following points:

We thank the Reviewer for their positive comments.

1. Introduction—spectral selection elements
The Introduction focuses on detector comparisons for implementing an on-chip IR spectroscopy system but omits the spectral-selection components (e.g., gratings/AWGs, metasurfaces, MEMS Fabry–Pérot, interferometric approaches). This makes the argument feel incomplete. Please briefly discuss candidate spectral elements and their trade-offs to strengthen the narrative.

Thank you for the comment.

In the proposed devices, an ultrathin metasurface is integrated into the RF MEMS to provide spectral selectivity. The metal-insulator-metal (MIM) metasurface absorbs IR with a specific spectral profile using plasmonic resonances. The absorbed power is converted into heat, which in turns changes the input impedance of the RF MEMS resonator. The ultra-thin and light-weight MIM-based absorber has little impact on the effective mass and the acoustic speed of the MEMS and thus it does not hinder its performance. The integration of metasurfaces with RF MEMS to achieve IR spectral selectivity has been widely studied in the literature[8, 9, 21, 39].

Alternative approaches to obtain spectral selectivity can be roughly divided into two main groups. On one hand, bulky approaches based on interferometry that rely on moving arms coupled with broadband IR detectors[1,2]. On the other hand, optical techniques that provide spectral selectivity before the radiation reaches the detector - like gratings or optical filters - which can also be bulky and might require extreme tunability and careful alignment.

We have not included the entire discussion in the updated version of the manuscript because it is already too long and key points can be found in the literature[8, 9, 21, 39. 40]. We can easily do so if the editor recommends it. However, to account for the Reviewer’s comment and our subsequent response, we have added the following sentences at the beginning of the second paragraph in the Introduction section:

In this context, thermal IR sensing based on RF MEMS loaded with ultrathin metasurfaces has attracted significant attention[17-26]. These devices embed IR spectral selectivity at the sensor level and avoid bulky alternatives like interferometry or optical filters/gratings that might be also difficult to align[27].

2. Reliance on broadband blackbody sources—what exactly is the issue?
You list reliance on broadband blackbody sources as a challenge, but the problem is stated abstractly. The device itself has spectral selectivity; however, a non-selective evaluation source means the measured signal is the convolution of the device's spectral responsivity with the blackbody spectrum. Please clarify whether your point is that this setup makes it difficult to fully demonstrate the device-intrinsic selectivity.

We thank the Reviewer for this comment. We fully agree. As stated by the Reviewer, using the common set-ups based on blackbody radiators, the detected signal is proportional to the convolution of the device's spectral responsivity with the blackbody source's IR spectrum. Therefore, this approach does not allow us to determine the sensor's wavelength dependent response, which is a key figure of merit.

In the literature, set-ups based on blackbody radiators are often complemented with the device absorption profile obtained using FTIR (Fourier Transform IR spectroscopy). Even though this technique resolves the spectral selectivity absorption of the device, it cannot determine how the detector responds to each individual wavelength. The use of blackbody radiators to characterize IR detectors has profound implications:

First, and according to Planck's law, blackbody radiation carries significantly higher power in the near-IR than at mid and far-IR wavelengths. Let us consider a detector designed to absorb mid-IR, as the one described in our manuscript. Even though the detector is not tuned to detect short wavelengths, powerful incoming radiation at these wavelengths will still be absorbed and converted into heat. Even if the absorbed power is less than 10% that the incident one, it may not be negligible compared to the power absorbed at the targeted wavelengths. This is because the IR power generated by a black body radiator in the mid-IR there is significantly lower than in the near IR. Thus, the interplay between wavelength-dependent power variations and non-zero out of band absorption responses might lead to a significant miscalculation of the detector's response.

Second, a blackbody source prevents meaningful power-dependent characterization. Note that the total beam power radiated from a blackbody radiator is non-equally spread across a range of wavelengths and thus will not be equal to the power generated at one particular wavelength by an IR laser. Thus, set-ups relying on black-body radiators don't allow to determine the detector spectral selectivity nor its power dependence.

And third, blackbody radiators generate IR beams with spot sizes that might be larger than the device under test and that cannot be easily focused as lenses do not possess a broadband response able to operate across the entire IR spectrum. As a result, is it possible that the surrounding silicon substrate is heated by the mid-IR and the shorter wavelength light. This heating is challenging to quantify and may mask the true response of the sensor.

To conclude, the use of a blackbody provides experimental uncertainties that prevent an adequate characterization of IR detectors. Our work addresses this important challenge by using a widely tunable IR laser coupled with an automatized power-dependent control set-up.

To account for the Reviewer's comment and our reply, we have included the following changes in our manuscript:

- In the second paragraph of the Introduction, we have included the sentences:

Second, many studies have emphasized the development of key device innovations instead of focusing on systematic sensing characterization procedures[5]. Figures of merit such as responsivity and NEP are often presented without considering their strong dependence on IR wavelength, power, or modulation frequency, leading to performance metrics that may not reflect realistic operation conditions. As a result, the performance limits of RF MEMS-based IR sensors remain unquantified and their potential for chip-integrated IR spectroscopy largely untapped.

- In the third paragraph of the Introduction, we have included the sentences:

As proof of concept, we demonstrate reflectometric 202 MHz lateral CMR MEMS detectors operating in the mid-IR and characterize them using frequency, wavelength, and power dependent approaches[5].

- At the end of the section "Responsivity", in Methods, we have added the following paragraph:

We emphasize that our characterization set-up does not rely on a blackbody radiator[17, 19, 20, 24]. In set-ups based on blackbody radiators, the detected signal is proportional to the convolution of the device's spectral responsivity with the blackbody source's IR spectrum. As a result, such set-ups do not allow us to determine the device's wavelength and power dependent response, which are key figures of merit. Additionally, blackbody radiators generate IR beams with spot sizes that can be significantly larger than the device under test, cannot be easily focused, and might heat the detector substrate. Such heating is difficult to quantify and may mask the true response of the sensor. All these challenges are addressed in this work by using the experimental set-up described above based on a widely tunable OPO IR laser.

3. Limits of conventional electrical readout

You suggest that conventional electrical readouts have difficulty resolving small RF shifts, but no quantitative limit is given. Please report representative values for the minimum detectable frequency shift in prior methods—and the determining factors (loop bandwidth, discriminator slope, frequency-noise spectral density)—and then show by how much the present method surpasses that limit. Even in the Introduction, a quantitative benchmark for "resolving small RF shifts" would help.

We thank the Reviewer for this comment.

In RF MEMS-based resonant sensors, frequency shifts induced by external stimuli are often small relative to the device's resonance frequency, typically on the order of parts per million (ppm)[41]. Resolving such shifts requires precise frequency measurement circuits, including phase-locked loops (PLLs), heterodyne detection, or frequency-discriminator-based schemes.

Conventional electrical readouts are fundamentally limited by three factors:

1. PLL loop bandwidth (f_{BW}): A wider bandwidth enables faster tracking but increases noise, while a narrower bandwidth reduces noise at the cost of slower response.
2. Discriminator slope (dV/df): The voltage output per unit frequency shift; a shallow slope limits sensitivity.
3. Frequency-noise spectral density ($S_f(f)$): Thermomechanical and electronic noise in the resonator sets a lower bound on resolvable shifts.

Frequency-shift measurements in micro- and nanomechanical resonators are therefore constrained both by resonator noise (e.g., Brownian motion causing random phase and frequency fluctuations) and by the readout circuitry (amplifier and detector noise). Moreover, the readout scheme imposes trade-offs between speed and precision: high loop bandwidth or fast update rates degrade frequency precision, whereas narrowing the bandwidth improves precision but slows response. Ref. [19] provides a detailed analysis of conventional tracking schemes, including feedback-free drive, self-sustaining oscillators, and PLLs, showing that the frequency stability (often quantified via Allan deviation) is ultimately limited by the interplay of loop bandwidth, discriminator slope, and resonator noise[19].

Representative (but not comprehensive) benchmarks from prior work illustrate these limits:

- In a high-precision resonant cavity displacement sensor operating near 275–750 MHz, heterodyne detection resolved frequency shifts limited by DAC step size to ~3 kHz (≈1100–400 ppm), which could be further refined to 0.5 kHz[42].

- PLL-based tracking of micromechanical cantilevers with ~12 kHz resonances resolved shifts of 83–200 Hz (≈6900–16,600 ppm)[43].

- Quartz crystal microbalance (QCM) systems at 10 MHz achieved 0.26 Hz resolution over measurable shifts of 0–2000 Hz, with the minimum detectable shift set by the ratio of Allan deviation to system sensitivity[44-45].

- In cryogenic VHF NEMS mass sensing (~190 MHz), frequency-modulated PLL detection resolved shifts on the order of 0–1000 Hz under vacuum and cryogenic conditions[46].

In this context, consider the frequency-shift responsivity of the RF MEMS employed in our work, 124 Hz/µW. Achieving a NEP of ~ 450 pW/Hz$^{1/2}$, as measured in our reflectometric device, would require resolving frequency shifts down to 0.056 Hz using RF frequency around 202 MHz. Such readout should not add any additional noise. Our "equivalent" frequency shift compares very

favorably with the metrics reported above but (i) this is an approximate comparison, as it neglects frequency and power dependence of the sensing metrics; and (ii) our literature review is extensive but not comprehensive. In any case, this simple study highlights again that reflectometry is able to drastically enhance the IR responsivity of RF MEMS-based sensors, and that achieving such performance in RF MEMS-based IR sensors based on frequency tracking would require the use of ultra-precise and low-noise PLL circuits that might not be available at higher RF frequencies (i.e., GHz or 100s of MHz).

In the broader context of IR sensing, we could not find any prior work that explicitly reported a "smallest detectable frequency shift" or any quantitative study. Such limits largely depend on frequency and are difficult to generalize. For instance, in Ref. [47] frequency shifts of 300 Hz are experimentally reported (Fig. 2c therein) for an electronic readout, whereas frequency shifts up to 10 kHz (Fig. 4d) are reported in other work that uses an optical readout[39]. A visual inspection of these measurements reveals that non-negligible noise is also present. Note that these frequency shifts do not correspond to the minimum that can be achieved in these devices, and that such values are not readability available in the literature. Additionally, and as previously discussed, the operation frequency of the RF MEMS can widely change from kHz to GHz which in turn has significant implications in the overall performance of the IR sensor. MEMS operating at lower RF frequencies will offer pretty low bandwidth, moderate responsivity, and low NEP as frequency shifts will be easier to track using PLL technology. RF MEMS operating at higher RF frequencies offer significantly larger responsivity and bandwidth, but frequency-shift tracking using PLL leads to much larger noise responses.

We have not included this discussion in the updated version of the manuscript because (i) our comparison analysis of RF MEMS sensors is approximate, in particular regarding the minimum frequency shift needed to achieve a particular NEP; and (ii) our manuscript is already too long. However, to account for the Reviewer's comment and our subsequent response, we have:

- Modified the second paragraph of the Introduction, which now includes

To date, the operation principle of RF MEMS-based IR sensors has relied on monitoring frequency shifts of specific mechanical modes upon IR illumination[28], using either optical[22-26] or electrical readouts[17-21], yielding NEP values down to ~ $\mathrm{pW}/\sqrt{\mathrm{Hz}}$ levels. In these devices, IR responsivity directly scales with the MEMS RF operation frequency and thermal resistance whereas the device size determines speed and bandwidth. Despite promising performance, several challenges have hindered a broader adoption of this technology. First, integrating readout mechanisms on-chip remains a challenge: optical schemes require bulky interferometric setups incompatible with chip-scale platforms and electrical readouts must resolve small RF shifts. This latter challenge imposes a performance trade-off in this technology. Devices operating at low RF frequencies usually offer limited bandwidth and IR responsivity but achieve ultralow NEP values by using precise frequency tracking based on phase-locked loop (PLL) circuits[28]. High-frequency RF MEMS, operating at GHz or 100s of MHz, provide larger responsivity and faster response. Nevertheless, PLL tracking becomes constrained at these frequencies by an elevated phase noise that increases NEP.

- Cited Bešić et al. (2023) (Ref. 22 here) in the main paper to refer the interested reader to a detailed analysis of conventional frequency tracking schemes and specific metrics.

4. Path to an on-chip spectrometer and thermal shift handling

Please outline how the device will be used as an on-chip spectrometer—for example, (i) an array of MEMS resonators with different absorption peaks for reconstructive spectroscopy, or (ii) a tunable element for scan-based spectroscopy. In addition, while the incident light changes the reactance via heating, does the absorption peak of the structure itself shift? If so, wavelength and intensity will couple, making spectral reconstruction nonlinear and requiring correction or inversion methods. If, under the intended operating flux, this effect is negligible, please state the evidence (e.g., a power sweep showing negligible peak shift).

Thank you for these comments.

Regarding the first point, we agree with the Reviewer in the different approaches that can be employed to construct an on-chip spectrometer with the proposed technology. First, one can use wavelength-tunable metasurface absorbers[48-50] to adjust the center wavelength targeted by the detector. Even though the electrical drive on the metasurface may induce some heat and temperature change on the device, a careful calibration procedure should resolve this challenge and capture the continuous IR spectrum across the tunable bandwidth. However, electrically tunable metasurfaces have a relatively small range and thus it is unlikely that just one device can serve as a spectrometer. The second approach consists of using an array of the proposed devices, each one decorated with a metasurface that targets a different IR wavelength. This requires some preliminary knowledge of the characteristic wavelength of the targeted objects like gas, plasma, etc. With such a priori information, the proposed devices can be tailored into the sensors for specific application scenarios such as gas sensor, cancer predictor, etc[51,52]. Moving beyond, hundreds of devices can be gathered together to construct a general-purpose on-chip IR spectrometer.

To account for this comment, we have added the following sentence at the end of the Discussion section:

For example, these devices can be functionalized with tunable IR metasurfaces[50] to enable spectrally selective IR scanning. Arrays of reflectometric MEMS-based detectors integrated within a thermally optimized package, each targeting a distinct IR wavelength, provide a compact and fully on-chip route toward reconstructive IR spectrometers and imaging systems, in which responsivity and saturation power can be dynamically reconfigured across IR bands to support adaptive and application-specific operation.

Regarding the second point, we highlight that the wavelength and intensity do not couple in this structure for moderate or low IR power. This is because the absorption profile of the device is defined by the electromagnetic properties of the materials that compose the metasurface (SiO2, Au). Entering into the nonlinear regime of these materials would require high-power beams that cannot be easily obtained in the IR beam, with peaks well over kW/cm levels[53]. Instead, high-

power IR beams can bring the device into the nonlinear regime in the sense that variations of the output do not linearly follow the incoming power.

More specifically, we demonstrated spectral selectivity in two different manners. First, we measured IR responsivity at different wavelengths using a widely tunable IR laser. As described in the Supplementary Information, a power control combined with a calibration system was employed to accurately determine the IR power employed at each wavelength. We have already employed the maximum IR power that our set-up can handle to make this study - limited in practice by the power that the IR laser can generate at the highest wavelength. Second, we measured the absorption profile of the device using a FTIR. As demonstrated in Fig. 2b, there is a remarkable agreement in the profile of these measurements.

Fig. 2a shows power-dependent IR measurements that require power-control only at 5.94 um. Results show that the device behaves in the expected linear regime for low and moderate IR power. For larger IR powers, the device enters a nonlinear regime in which the phase variations of the reflected RF signal do not vary linearly with the power of the incoming IR beam. Such nonlinear response is fully captured with a reflectometric RF circuit model (see Supplementary Information, Section 3.1 and Fig. S12). Note that the maximum IR power employed in our study is quite moderate, with values up to 300 uW, but still larger than the power required to go above the damage threshold of conventional semiconductor heterostructures. If a nonlinear coupling between wavelength and intensity appears, then our power-controlled experiment (Fig. S12) would have revealed a quite different nonlinear regime. Taking all these factors together, i.e., the absorption mechanism on which the detector is based (metasurfaces), spectral selectivity experiments, and the power-controlled studies, it is clear that wavelength and intensity do not couple in our sensors. Such coupling might appear at much higher IR power levels.

To account for this comment, we have added the following sentence when describing Fig. 2a:

This nonlinear regime originates from the finite bandwidth of the RF resonance and not from the intrinsic nonlinear response of the materials composing the RF MEMS/metasurface structure.

References

1. Dereniak, E. & Boreman, G. Infrared Detectors and Systems. in (1996).
2. Rogalski, A. *Infrared and Terahertz Detectors, Third Edition*. (CRC Press, Boca Raton, 2019). doi:10.1201/b21951.
3. Rogalski, A., Kopytko, M. & Martyniuk, P. Two-dimensional infrared and terahertz detectors: Outlook and status. *Applied Physics Reviews* **6** (2019).
4. Rogalski, A. Commentary on the record-breaking performance of low-dimensional solid photodetectors. *ACS Photonics* 10, 647-653 (2023).
5. Lukman, S. et al. High oscillator strength interlayer excitons in two-dimensional heterostructures for mid-infrared photodetection. Nature Nanotechnology 15, 675-682 (2020).
6. Yu, X. et al. Narrow bandgap oxide nanoparticles coupled with graphene for high performance mid-infrared photodetection. Nature Communications 9, 4299 (2018).
7. Liu, T., Tong, L., Huang, X. & Ye, L. Room-temperature infrared photodetectors with hybrid structure based on two-dimensional materials. Chinese Physics B 28, 017302 (2019).

8 Hui, Y., Kang, S., Qian, Z. & Rinaldi, M. Uncooled Infrared Detector Based on an Aluminum Nitride Piezoelectric Fishnet Metasurface. *J. Microelectromechanical Syst.* **30**, 165–172 (2021).

9 Gülseren, M. E. *et al.* Experimental Study of Spectrally Selective MEMS/Metasurface Infrared Detectors. *IEEE Sens. J.* **24**, 17313–17323 (2024).

10. Mohd-Yasin, F., Nagel, D. J., Mohd-Yasin, F. & Nagel, D. J. Noise as Diagnostic Tool for Quality and Reliability of MEMS. *Sensors* **21**, (2021).
11. Fang, Y., Armin, A., Meredith, P. & Huang, J. Accurate characterization of next-generation thin-film photodetectors. *Nat. Photonics* **13**, 1–4 (2019).
12. Liu, Y.-Y. *et al.* Radio-Frequency Reflectometry in Silicon-Based Quantum Dots. *Phys. Rev. Appl.* **16**, 014057 (2021).
13. Filmer, M. J. *et al.* Gate reflectometry of single-electron box arrays using calibrated low temperature matching networks. *Sci. Rep.* **12**, 3098 (2022).
14. Petersan, P. J. & Anlage, S. M. Measurement of resonant frequency and quality factor of microwave resonators: Comparison of methods. *J. Appl. Phys.* **84**, 3392–3402 (1998).
15. Bagci, T. *et al.* Optical detection of radio waves through a nanomechanical transducer. *Nature* **507**, 81–85 (2014).
16. Truitt, P. A., Hertzberg, J. B., Huang, C. C., Ekinci, K. L. & Schwab, K. C. Efficient and Sensitive Capacitive Readout of Nanomechanical Resonator Arrays. *Nano Lett.* **7**, 120–126 (2007).
17. Zhang, C. *et al.* High detectivity terahertz radiation sensing using frequency-noise-optimized nanomechanical resonators. *APL Photonics* **9**, 126105 (2024).
18. Zhang, C. *et al.* Enhanced Bandwidth in Radiation Sensors Operating at the Fundamental Temperature Fluctuation Noise Limit. *Nano Lett.* **25**, 14660–14667 (2025).

19. *Piezoelectric MEMS Resonators*. (Springer International Publishing, Cham, 2017). doi:10.1007/978-3-319-28688-4.

20. Piazza, G. Contour-Mode Aluminum Nitride Piezoelectric MEMS Resonators and Filters. in *MEMS-based Circuits and Systems for Wireless Communication* (eds Enz, C. C. & Kaiser, A.) 29–54 (Springer US, Boston, MA, 2013). doi:10.1007/978-1-4419-8798-3_2.

21. Tao, J. *et al.* Dual functionality metamaterial enables ultra-compact, highly sensitive uncooled infrared sensor. *Nanophotonics* **10**, 1337–1346 (2021).

22. Bešić, H., Demir, A., Steurer, J., Luhmann, N. & Schmid, S. Schemes for Tracking Resonance Frequency for Micro- and Nanomechanical Resonators. *Phys. Rev. Appl.* **20**, 024023 (2023).

23. Rahim, K. & Mian, A. A Review on Laser Processing in Electronic and MEMS Packaging. *J. Electron. Packag.* **139**, (2017).

24. Tilmans, H. A. C. *et al.* MEMS packaging and reliability: An undividable couple. *Microelectron. Reliab.* **52**, 2228–2234 (2012).

25. Razavi, B. A study of phase noise in CMOS oscillators. *IEEE J. Solid-State Circuits* **31**, 331–343 (1996).

26. Miller, J. M. L. *et al.* Effective quality factor tuning mechanisms in micromechanical resonators. *Appl. Phys. Rev.* **5**, 041307 (2018).

27. Navy - 23.2 SBIR - Mid-Wave Infrared Detectors with Tunable Narrow-Band Spectral Response. https://navysbir.us/n23_2/N232-104.htm#qa.

28. Le Galudec, J., Dupoy, M., Duraffourg, L., Rebuffel, V. & Marcoux, P. R. Microbial Identification Through Multispectral Infrared Imaging of Colonies: A New Type of Morpho-Spectral Fingerprinting. *Microb. Biotechnol.* **18**, e70093 (2025).

29. Jang, W.-Y. *et al.* Experimental Demonstration of Adaptive Infrared Multispectral Imaging using Plasmonic Filter Array. *Sci. Rep.* **6**, 34876 (2016).

30. Sigrist, M. W. Mid-infrared laser-spectroscopic sensing of chemical species. *J. Adv. Res.* **6**, 529–533 (2015).

31. Wang, L. *et al.* Filterless and High-Speed InP Near-Infrared Photodetector With an Ultra-Small Full-Width at Half Maximum. *IEEE Trans. Electron Devices* **70**, 3145–3148 (2023).

32. Nadeem, A., Lyons, S., Kindopp, A., Jamieson, A. & Roxbury, D. Machine Learning-Assisted Near-Infrared Spectral Fingerprinting for Macrophage Phenotyping. *ACS Nano* **18**, 22874–22887 (2024).

33. Hasbi, N. H. *et al.* Pattern Recognition for Human Diseases Classification in Spectral Analysis. *Computation* **10**, (2022).

34. *Piezoelectric MEMS Resonators*. (Springer International Publishing, Cham, 2017). doi:10.1007/978-3-319-28688-4.

35. Hou, Y. *et al.* A review: aluminum nitride MEMS contour-mode resonator*. *J. Semicond.* **37**, 101001 (2016).

36. Segovia-Fernandez, J. & Piazza, G. Thermal Nonlinearities in Contour Mode AlN Resonators. *J. Microelectromechanical Syst.* **22**, 976–985 (2013).

37. Tazzoli, A., Rinaldi, M. & Piazza, G. Experimental Investigation of Thermally Induced Nonlinearities in Aluminum Nitride Contour-Mode MEMS Resonators. *IEEE Electron Device Lett.* **33**, 724–726 (2012).

38. Kim, H. J., Jung, S. I., Segovia-Fernandez, J. & Piazza, G. The impact of electrode materials on 1/f noise in piezoelectric AlN contour mode resonators. *AIP Advances* **8** (2018).

39. Venditti, A. *et al.* Plasmonically Enhanced Flexural-Mode AlScN Nanoplate Resonator as Uncooled and Ultrafast IR Detector with High Responsivity. *arXiv preprint arXiv:2506.21412* (2025).

40. Bahaa E. A. Saleh, Malvin Carl Teich, *Fundamentals of Photonics*, John Wiley & Sons. *NY, USA* (1991).

41. Bahreyni, B. & Shafai, C. Oscillator and frequency-shift measurement circuit topologies for micromachined resonant devices. *Sens. Actuators Phys.* **137**, 74–80 (2007).

42. Jauregui, R., Asua, E., Portilla, J. & Etxebarria, V. Reliable and integrated technique for determining resonant frequency in radio frequency resonators. Application to a high-precision resonant cavity-based displacement sensor. *Rev. Sci. Instrum.* **86**, 034709 (2015).

43. Cakmak, O., Ermek, E., Kilinc, N., Yaralioglu, G. G. & Urey, H. Precision density and viscosity measurement using two cantilevers with different widths. *Sens. Actuators Phys.* **232**, 141–147 (2015).

44. Gao, C. *et al.* A High-Performance Miniaturized Frequency Shift Detection System for QCM-Based Gravimetric Sensing. *Adv. Sens. Res.* **4**, 2400148 (2025).

45. Park, J.-Y. *et al.* A Miniaturized Quartz Crystal Microbalance (QCM) Measurement Instrument Based on a Phase-Locked Loop Circuit. *Electronics* **11**, (2022).

46. Yang, Y. T., Callegari, C., Feng, X. L., Ekinci, K. L. & Roukes, M. L. Zeptogram-Scale Nanomechanical Mass Sensing. *Nano Lett.* **6**, 583–586 (2006).

47. Hui, Y., Gomez-Diaz, J. S., Qian, Z., Alù, A. & Rinaldi, M. . *Nat. Commun.* **7**, 11249 (2016).

48. Wang, X. *et al.* Micromachined Tunable High-Q Infrared Absorbers Enabled by Free-Standing All-Metallic Metasurfaces. *ACS Photonics* **11**, 3870–3877 (2024).

49. Yao, Y. *et al.* Electrically Tunable Metasurface Perfect Absorbers for Ultrathin Mid-Infrared Optical Modulators. *Nano Lett.* **14**, 6526–6532 (2014).

50. Abdelraouf, O. A. M., Wang Z., Liu H., Dong Z., Qian W., Ye M., Wang X. R., Wang Q. J., Liu H., Recent Advances in Tunable Metasurfaces: Materials, Design, and Applications*, ACS Nano* ***16**, 13339–13369 (2022).*

51. Fu, W. *et al.* Raman spectroscopy and machine learning for early detection of gastric cancer and Helicobacter pylori with gastric juice. *Sci. Rep.* **15**, 36332 (2025).

52. Jha, R. K. Non-Dispersive Infrared Gas Sensing Technology: A Review. *IEEE Sens. J.* **22**, 6–15 (2022).

53. R. Boyd, Nonlinear Optics, Academic Press, 2020.

We would like to thank the Associate Editor and the Reviewers for their time and valuable comments which greatly helped us to improve the quality of our manuscript. In this document we answer in detail each of the comments and describe the corresponding changes made to the manuscript.

Legends

This document uses the following color codes:

• Black: original comments of the reviewer.
• Blue: response from the authors.
• Red: changes introduced in the manuscript.

Reviewer #1 (Remarks to the Author):
--

The authors have addressed most of my concerns and have convinced me that the paper can be published in Nature Communications, as it presents, for the first time, a reflectometric readout of an RF-MEMS IR detector. This is particularly useful for high-frequency piezoelectric resonators. The only remaining issue I have is the discussion of responsivity vs. NEP (signal vs. SNR) as the key figure of merit. I recommend publishing the manuscript. I would, however, recommend that this issue be addressed in a revised version.

We would like to thank the Reviewer for the insightful feedback and suggestions that have helped us increase the quality of our manuscript. We are particularly grateful for the Reviewer's positive assessment and truly appreciate their revised view regarding the suitability of our work for publication in *Nature Communications*.

Here are some concrete points:

- Responsivity in units [V/W] is not a measure of sensitivity without knowing the corresponding noise. On the one hand, the signal could be amplified or depend on a bias voltage, boosting the value without improving sensitivity. On the other hand, the noise could be reduced, e.g., through parallel measurements (possible if the noise is uncorrelated), thereby improving sensitivity while the responsivity remains constant. The sensitivity of an IR detector is given by the NEP (you are correct that D* does not apply for thermal detectors, but only as long as they are not operated at the temperature fluctuation noise limit).

We thank the Reviewer for this insightful comment. We fully agree that the Noise Equivalent Power (NEP) is a critical performance metric, as it ultimately defines the minimum detectable signal and the fundamental sensitivity of an IR detector. We acknowledge that our discussion in the introduction was mainly focused on responsivity, and we agree that a comprehensive performance assessment is incomplete without the inclusion of the NEP.

In our manuscript, we wish to reinforce that high responsivity remains a key engineering requirement for IR sensing in realistic applications. While the NEP defines the theoretical sensitivity limit, high responsivity is essential to overcome the electronic noise floor of interfacing electronics and enable operation near the detector's intrinsic NEP.

To address the Reviewer's comment, the updated version of the manuscript has been modified as follows:

- We have included NEP values for all IR sensing technologies discussed in the Introduction, including narrow-bandgap semiconductors, 2D materials, and bolometers.
- Throughout the revised manuscript, we have ensured that the sensors' IR responsivity metrics are consistently benchmarked against their corresponding NEP values to provide a balanced performance overview. This ensures a more rigorous and balanced representation of sensor performance, allowing for a direct assessment of sensitivity relative to the noise floor.
- We have modified the last part of the Introduction as follows:

  Ultimately, translating IR sensing technologies into scalable, cost-effective, and on-chip IR spectroscopy and sensing systems[1,2,16] requires transitioning from discrete, broadband devices to integrated, spectrally-selective detectors. A high responsivity is essential to overcome the noise floor of the interfacing electronics and enable operation near the detector's intrinsic NEP.

- On line 36, you mention that a high responsivity could lift the sensitivity into the BLIP regime. BLIP applies only to photodetectors, not to thermal detectors, because it usually implies reaching the shot noise limit (which is not possible with RT thermal detectors). And, to make my point about signal vs. SNR: every BLIP discussion in IR detector textbooks (e.g., Rogalski) is and has to be conducted by the NEP or D*, representing the SNR of the detector. Only the SNR can tell if a detector is operating in the BLIP regime; the responsivity alone can not.

We thank the Reviewer for this comment.

We agree that a high responsivity alone does not guarantee operation in the BLIP regime. To assess potential BLIP operation, the NEP should be evaluated together with the background noise to establish the true limit of detection. Additionally, we agree that the specific mention of the BLIP regime is not critical to the primary message of the introduction or our manuscript. Accordingly, we have removed this discussion (lines 36-38 in the Introduction) to make the opening paragraph clearer and more direct.

Regarding the term 'BLIP' (Background Limited Infrared Photodetector), we note that it has historically been associated with photon detectors. However, the underlying physical regime describes a scenario in which the background radiation noise floor exceeds the detector's intrinsic noise. This represents a fundamental limit shared by thermal detectors as is explicitly stated in the literature [1]. In the case of thermal sensors, this state is also referred to as being radiation-noise limited or operating at the thermal fluctuation limit. To ensure clarity, as mentioned above, we have removed any references to the 'BLIP' regime in the revised manuscript.

- On line 44 and subsequent lines, it is correctly mentioned that the absolute IR responsivity, in units of [Hz/W], of MEMS/NEMS resonators scales with the resonance frequency. But the absolute IR responsivity in [Hz/W] is not an indication of performance. It is fractional resonant frequency changes that matter, which are typically characterized by the fractional Allan deviation of an oscillator, in units of [1/W]. This is similar to the signal vs. SNR discussion.

We thank the Reviewer for this comment. As stated before, we agree that IR responsivity alone is not sufficient as a metric of performance and that the device's noise equivalent power needs to be evaluated to determine the detection limit. The expressions that describe frequency-shift responsivity and thermal NEP of RF MEMS-based IR sensors are given by (see Supplementary Information of the manuscript)

$$\mathrm{R_f}(\omega,\lambda) = \frac{\Delta \mathrm{f}(\omega,\lambda)}{\mathrm{P_{in}}(\lambda)} = \mathrm{f_0} \cdot \mathrm{TCF} \cdot \eta(\lambda) \cdot \chi(\omega) \cdot \mathrm{R_{th}} \quad [\mathrm{Hz/W}], \tag{1}$$

$$\mathrm{NEP_{thermal}} = \sqrt{4 \mathrm{k_B} \mathrm{T}^2 \mathrm{R_{th}^{-1}}} \quad [\mathrm{W}/\sqrt{\mathrm{Hz}}], \tag{2}$$

where $\mathrm{f_0}$ is the device's resonance frequency; TCF is the temperature coefficient of frequency of the RF MEMS resonator; $\eta(\lambda)$ is the wavelength dependent absorptance; $\chi(\omega) = \left(1 + \omega^2 \mathrm{C_{th}^2 R_{th}^2}\right)^{-\frac{1}{2}}$ accounts for the device time response versus the modulation frequency ω of the incoming IR beam; $\mathrm{R_{th}}$ is the resonator's thermal resistance; T is temperature; and $\mathrm{k_B}$ is the Boltzmann constant.

Equations (1) and (2) reveal that increasing the thermal resistance ($\mathrm{R_{th}}$) is critical for enhancing the performance of thermal IR sensors, as it simultaneously increases responsivity and decreases the fundamental thermal NEP. However, physical constraints prevent increasing thermal resistance beyond the limits imposed by the mechanical stability of the suspended membranes and the minimum thermal conductance of the electrical interconnects. We also concur with the Reviewer that the operation frequency is not a primary driver of the detector's fundamental NEP. We also note that in our proposed reflectometric MEMS-based IR sensors, responsivity and NEP do not depend on the operation frequency.

To account for the Reviewer's comment and our subsequent response, we have modified the sentence in line 44 to avoid mentioning the device operation frequency. It reads:

In these devices, IR responsivity and thermal NEP scale directly with and inversely with the square root of thermal resistance, respectively, whereas the device size determines speed and bandwidth.

Furthermore, we agree that the fractional resonant frequency change is the primary metric of interest. In our brief summary of RF MEMS for IR sensing, we have not explicitly employed the fractional Allan deviation as we are focusing on the fundamental limits of the MEMS detector itself rather than on specific implementations. By describing the intrinsic response of the resonator, our analysis remains valid regardless of whether the device is operated in a closed-loop oscillator configuration or in an open-loop passive setup. To clarify the implications of these two different readout strategies, we provide a brief definition of these two cases below [2]:

- In a *closed-loop configuration*, the RF MEMS is integrated into a feedback circuit that includes a sustaining RF amplifier to create a self-sustaining oscillator. This scenario has been well studied and applied in the literature [2-5]. In this operation mode, the fractional Allan deviation, $\sigma_y(\tau)$, serves as the standard metric for characterizing system-level frequency stability, as it accounts for both the resonator's intrinsic thermal-mechanical noise and the phase noise added by the electronics. The Allan deviation effectively

defines the frequency-domain noise floor, enabling the calculation of the system's minimum detectable power and resulting limit of detection.

- In an *open-loop configuration*, the RF MEMS operates as a passive resonant filter driven by an external source, such as a signal generator or a vector network analyzer. In this regime, the fractional Allan deviation is not typically employed as a performance metric, as the system does not generate a self-sustained frequency. Instead, the frequency stability is characterized by the precision with which the resonance peak can be resolved against the measured noise floor [2, 6-9]. The limit of detection in open-loop is dictated by the ability to track the center frequency in the presence of additive electronic noise and external drive instabilities rather than the oscillator-limited noise floor.

- On line 58, the authors mention that "the performance limits of RF MEMS-based IR sensors remain unquantified". I disagree with this statement. There have been many papers published in recent years, among them those from the St-Gelais group in Ottawa, Cleland and Roukes, etc, that clearly lay out the physical limits of resonant IR detectors. The fundamental limits of these detectors are temperature fluctuation noise and thermomechanical noise, which have both been clearly understood and experimentally demonstrated

We thank the Reviewer for this point and agree that the fundamental physical limits of RF MEMS-based IR detectors have been previously determined both theoretically and experimentally. Some relevant works, including those from the research groups mentioned by the Reviewer, are listed in Refs [3-14]. Our original sentence was intended to describe the system-level performance in the context of on-chip thermal IR sensing, rather than the intrinsic limits of an isolated detector.

To account for the Reviewer's comment and our subsequent response, we have:

- Modified the sentence in line 58 as follows:

As a result, the potential performance of RF MEMS-based IR sensors for chip-integrated IR spectroscopy has yet to be fully explored in practice.

- Added additional references that describe individual MEMS-based IR sensors, including their fundamental limits [10-14].

Reviewer #2 (Remarks to the Author):

While I find the responses satisfactory and recommend acceptance, I note that some of the key clarifications provided in the rebuttal would strengthen the paper if partially reflected in the main text.

We thank the Reviewer for their positive assessment. We agree that the clarifications provided in our previous response further strengthen the manuscript's results and conclusions. While the primary narrative is currently at the journal's length limit, we have integrated these detailed explanations into the Methods section (part of the main text) and the Supplementary Information. This approach ensures that key technical details from our rebuttal are permanently available to the reader. Specifically:

- The discussion regarding alternative spectral selective elements for on-chip IR spectroscopy has been included in Methods – Absorption profile. This corresponds to point 1 of the Reviewer's previous comment.
- The discussion regarding the challenges of using blackbody sources to characterize IR detectors was already included in the main paper and Methods – Responsivity.
- The discussion regarding the limits of conventional frequency-shift readouts for RF MEMS IR sensing has been added in Methods – Responsivity. This corresponds to point 3 of the Reviewer's previous comment.
- The discussion regarding the potential dependence of the device absorption profile with respect to the power of the incoming IR light has been added in the Supplementary Information – 4.6 Power-independent absorption profile. This corresponds to point 4 of the Reviewer's previous comment.

We also note that the complete history of the review process will be accessible to interested readers to provide further context on these points.

References

[1] Kruse, P. W. Uncooled thermal imaging: arrays, systems, and applications. Vol. 51 (SPIE press, 2001). Chapter 2.
[2] Bešić, H., Demir, A., Steurer, J., Luhmann, N. & Schmid, S., Schemes for Tracking Resonance Frequency for Micro- and Nanomechanical Resonators, *Physical Review Applied*, **20**, 024023 2023.
[3] L. Xu, J. Xi, F. Li, J. Pi, C. Li, K. Wang, X. Xiong, Y. Wang, H. Liu, Z. Zou, and C. Zhao, A Closed-Loop System for Resonant MEMS Sensors Subject to Blue-Sideband Excitation, *Journal of Microelectromechanical Systems* **31**, n4, pp. 690-699, 2022.
[4] Zhang, C., Louis-Seize, Z., Saleh, Y., Brazeau, M., Hodges, T., Turgeon-Roy, M., St-Gelais, R., Enhanced Bandwidth in Radiation Sensors Operating at the Fundamental Temperature Fluctuation Noise Limit, *Nano Letters* **25**, 14660-14667, 2025.
[5] Martini, P. *et al.* Uncooled thermal infrared detection near the fundamental limit using a silicon nitride nanomechanical resonator with a broadband absorber. *Communications Physics* **8**, 166 2025.
[6] Hui, Y., Gomez-Diaz, J. S., Qian, Z., Alu, A. & Rinaldi, M., Plasmonic piezoelectric nanomechanical resonator for spectrally selective infrared sensing. *Nature Communications* 7, 11249, 2016.
[7]Hui, Y., Kang, S., Qian, Z. & Rinaldi, M. Uncooled infrared detector based on an aluminum nitride piezoelectric fishnet metasurface. *Journal of Microelectromechanical Systems* **30**, 165-172, 2021.
[8]Tao, J. et al. Dual functionality metamaterial enables ultra-compact, highly sensitive uncooled infrared sensor. *Nanophotonics* **10**, 1337-1346, 2021.
[9]Gülseren, M. E. et al. Experimental Study of Spectrally-Selective MEMS/metasurface Infrared Detectors *IEEE Sensors Journal* **24**, 17313 – 17323, 2024.
[10] Z. Chang and R. St-Gelais, Demonstration of frequency stability limited by thermal fluctuation noise in silicon nitride nanomechanical resonators, *Applied Physics Letters* **122**, 193501, 2023.
[11] C. Zhang, E. K. Yalavarthi, M. Giroux, W. Cui, M. Stephan, A. Maleki, A. Weck, J. M. Menard, R. St-Gelais, High detectivity terahertz radiation sensing using frequency-noise-optimized nanomechanical resonators, *APL Photonics* **9**, 126105, 2024.
[12] N. Snell, C. Zhang, G. Mu, A. Bouchard, R. St-Gelais, Heat Transport in Silicon Nitride Drum Resonators and its Influence on Thermal Fluctuation-Induced Frequency Noise, *Physical Review Applied* 17, 044019, 2022.
[13] A. N. Cleland, Foundations of Nanomechanics: From Solid-State Theory to Device Application, Spriger, 2002.
[14] A. N. Cleland and M. L. Roukes, Fabrication of high frequency nanometer scale mechanical resonators from bulk Si crystals, *Applied Physics Letters* **69**, 2653-2655, 1996.
